# Acceptance and commitment therapy processes and their association with distress in cancer: a systematic review and meta-analysis

**DOI:** 10.1080/17437199.2023.2261518

**Published:** 2023-09-25

**Authors:** Sophie Fawson, Zoe Moon, Katherine Novogrudsky, Faye Moxham, Katie Forster, Insun Tribe, Rona Moss-Morris, Caroline Johnson, Lyndsay D. Hughes

**Affiliations:** a Psychology Department, King’s College London, London, UK; b NIHR Maudsley Biomedical Research Centre, London, UK; c School of Pharmacy, University College London, London, UK; d Imperial College Healthcare NHS Trust, London, UK

**Keywords:** Cancer, oncology, meta-analysis, distress, acceptance and commitment therapy, self-compassion

## Abstract

Around 42% of individuals with cancer experience distress. Acceptance and commitment therapy (ACT) can reduce distress, but effects are small, and mechanisms unclear. This review aimed to identify associations between ACT processes and distress in cancer. Search terms included cancer, ACT processes, self-compassion, and distress. Six online databases and grey literature were searched until March 2022. Of 6555 papers screened, 108 studies were included with 17,195 participants. Five meta-analyses of 77 studies were conducted. Random effects meta-analyses of correlations revealed higher scores on flexible processes (acceptance, present moment awareness, self-compassion) were associated with lower distress (*r*_pooled_ = -0.24, -0.39, -0.48, respectively); whilst higher scores on inflexible processes (experiential avoidance, cognitive fusion) were associated with higher distress (*r*_pooled_ = 0.58, 0.57, respectively). Meta-analyses displayed moderate-to-high heterogeneity with most studies assessed as low risk of bias. Meta-regressions revealed no significant moderators (stage, time since diagnosis, gender and age). This review provides a theoretically aligned evidence base for associations between ACT processes and distress in cancer, supporting elements of ACT theory and providing targeted directions for intervention development. Due to limited evidence, future research should focus on self-as-context, values and committed action and conduct mediation analysis in controlled trials of ACT processes on distress in cancer.

## 1. Introduction

Cancer poses a significant emotional, financial and social burden on individuals. Incidence is rising worldwide with 19.3 million new cases in 2020 and annual economic costs estimated at US$1.16 trillion (Sung et al., 2021; *World Cancer Report: Cancer Research for Cancer Prevention*, 2020; World Health Organization, 2021). There are many challenges associated with cancer diagnosis and treatment which can cause significant distress to patients, including increased medical appointments and unpleasant procedures, psychological and physical symptoms and fears around recurrence, spread and the future (Mathew et al., 2021; Niedzwiedz et al., 2019). Psychological or emotional distress, often characterised by symptoms of anxiety and depression, can be defined as an emotional state of suffering (Drapeau et al., 2012). Distress is up to twice as prevalent in cancer populations than in the general population, with estimates of 17-42% for anxiety symptoms and 4-24% for depressive symptoms, persisting throughout diagnosis, primary treatment, palliative treatment and long-term survivorship (Brandenbarg et al., 2019; Brunckhorst et al., 2021; Hashemi et al., 2020; Hinz et al., 2010; Krebber et al., 2014; Linden et al., 2012; Mitchell et al., 2013; Niedzwiedz et al., 2019; Walker et al., 2013).

High levels of distress in individuals with cancer can result in higher personal and healthcare costs and poorer health outcomes in terms of reduced quality of life, treatment non-adherence, poorer rates of recurrence and survival (DiMatteo & Haskard-Zolnierek, 2011; Fangand & Schnoll, 2002; Onitilo et al., 2006). Guidelines worldwide recommend that distress is routinely measured based on the premise that identifying, evaluating, and managing distress in patients with cancer is as important as physical health care (National Comprehensive Cancer Network, NCCN, Holland & Bultz, 2007; CSG4, National Institute for Clinical Excellence, 2004).

Research exploring Acceptance and commitment therapy (ACT) as an approach to managing distress in cancer has increased over the last 10 years. ACT is a third-wave cognitive behavioural approach aiming to increase psychological flexibility; defined as the ability to be open and aware in the present moment whilst engaging in meaningful, valued activity (Graham et al., 2016; Hayes et al., 2006). ACT is an approach that evolved from but differs from conventional Cognitive behavioural therapy (CBT). ACT focuses on our relationship with or responses to thoughts and emotions rather than on disrupting or changing unhelpful thoughts and emotions, which can exacerbate distress (Harris, 2019; Hayes et al., 2006; Ruiz, 2010). ACT is proposed to work through increasing psychological flexibility, which is the ability to fully connect with the present moment in order to engage behavioural patterns supporting movement towards valued ends (Hayes et al., 2006). ACT encompasses six core flexible processes or psychological skills (Bennett & Oliver, 2019; Graham et al., 2016; Harris, 2019; Hayes et al., 2013; Hayes et al., 2006; Hayes et al., 2011). *Acceptance* is the awareness and willingness to experience private thoughts, feelings and emotions without trying to change or control them. *Defusion* refers to changing the relationship with, or detaching from thoughts, images and memories in order to alter their function and reduce their literal quality; and developing the ability to recognise thoughts as thoughts and not fact. *Present moment awareness* is the non-judgemental contact with the internal and external world such that an individual can consciously connect with and engage in whatever is happening in the present moment. *Self-as-context* is the awareness of one’s own internal experience without attachment and the recognition of the self as the context from which thoughts, beliefs, emotions and memories can be observed. *Values* are qualities of ongoing action based on knowing what matters to an individual. Lastly, *committed action* refers to workable values driven goals and action. The six flexible processes have corresponding inflexible processes (collectively termed as psychological inflexibility) contributing to psychopathology and suffering (Hayes et al., 2006; Hayes et al., 2013). These are *experiential avoidance,* which refers to the attempts to change or avoid uncomfortable thoughts or emotions; *cognitive fusion*, which is seeing the thought as an absolute reflection of reality such that it dominates awareness and influences behaviour; *loss of contact with the present moment,* whereby individuals cannot connect with and engage in the present moment; *self-as-content*, which refers to being attached to our internal experience and stuck with stories we have about ourselves; *lack of awareness of one’s values* or remoteness from values; and *inaction*, which is unworkable action that is not guided by values (Hayes et al., 2006; Hayes et al., 2013). Self-compassion is implied throughout the model and, although not an explicit process, is a close concept and key feature of ACT in practice (Neff & Tirch, 2013). Self-compassion is the process of being kind and understanding towards oneself in difficult situations, whilst holding thoughts and feelings non-judgementally and mindfully, as though part of a wider human experience (Neff, 2003).

ACT may be a particularly useful approach for patients with cancer. ACT views distress and suffering as normal parts of the universal human experience and sees difficult thoughts and emotions as common and realistic responses to a cancer diagnosis. ACT seeks to encourage the development of psychological flexibility through processes such as valued living, enabling individuals to pursue meaningful activity even in the presence of difficulties such as distress and disruptions to daily living caused by treatment, medical appointments and recovery time whilst living with cancer (González-Fernández & Fernández-Rodríguez, 2019). ACT has also been shown to effectively support those with chronic pain and sleep difficulties, symptoms commonly associated with cancer treatment (Mosher et al., 2018; NG193, National Institute for Clinical Excellence, 2021) and although not the main aim of ACT, symptom or distress reduction is often found to be a secondary benefit (Graham et al., 2016). Whilst there are a number of techniques associated with ACT, it can be delivered in a protocolised format, and this is commonly undertaken in research trials to ensure fidelity. However, ACT is a process-oriented psychological intervention and experienced clinicians will conduct sessions in a process-focussed manner, basing their intervention on dynamic assessments of a client’s presentation and moving between ACT processes as indicated (Bennett & Oliver, 2019). Therefore, understanding the relevant impact and contribution of the processes can aid the formulation of this dynamic assessment.

Several reviews have sought to determine the effectiveness of ACT interventions in cancer with different outcomes, although the results were only narratively synthesised. In a systematic review, Mathew et al. (2021) found small positive effects in terms of anxiety and depression in 13 trials evaluating ACT for cancer survivors. Furthermore, a review of 19 trials of all cancer types and stages by González-Fernández and Fernández-Rodríguez (2019) reported significant improvements in emotional states (anxiety, depression, emotional distress) and quality of life following ACT intervention, including up to 12 months later. Similar conclusions have been reported in systematic reviews and meta-analyses of ACT-based interventions for other long term physical conditions with some large effects found for reductions in distress (Graham et al., 2016; Ngan et al., 2021).

Although these reviews provide promising evidence for the potential effectiveness of ACT interventions for reducing distress, the included trials are often small, low quality, and with limitations to study design (Fashler et al., 2018; González-Fernández & Fernández-Rodríguez, 2019; Hulbert-Williams, Storey, & Wilson, 2015; Salari et al., 2021). The impact and contribution of these reviews are therefore limited as meta-analyses could not be conducted due to variation in study designs (i.e., limited randomised controlled trials) and outcome measures, meaning pooled effect sizes are not reported. In addition, the included trials do not evaluate processes of change. ACT identifies likely mechanisms of action, however ideally, mediation analysis of ACT trials in cancer is needed to provide empirical support for the proposal of the key mechanisms of change through which interventions act on distress. A theoretical understanding of these processes of change in relation to context, is a vital part of the updated Medical Research Council (MRC) framework for developing complex interventions (Skivington et al., 2021) and may improve the efficacy of future interventions by building support for which of the ACT processes are amenable to change, and which are needed to change to improve a specific outcome. Identifying the change in processes which elicits a change in outcomes from the intervention will help identify the key mechanisms for those with cancer. Exploring the evidence-base for specific ACT processes may also improve accessibility and cost-effectiveness as there is potential for them to be used in briefer more targeted interventions for patients who do not necessarily need specialist interventions delivered by an experienced clinician (Dindo et al., 2017; Richards, 2012). Alternatively, processes could be flexibly incorporated into other theoretically consistent interventions. In the absence of published mediation studies of randomised controlled trials which would be the strongest level of evidence, associations between ACT processes and distress in observational studies in cancer can be explored and meta-analysed. This provides a first step in highlighting the ACT model in the context of cancer distress and the processes which may be key to target in future interventions.

To ensure the validity of a theory underlying an intervention, the theory needs to be tested and supported with a solid evidence base and the components of models should also be tested in studies, in different contexts, before interventions are conducted (Hayes et al., 2013; Levin et al., 2012). Data in the context of ACT in cancer is less well established than in mental health and pain (Dindo et al., 2017), so this review aims to provide the first step to a thorough understanding of ACT processes in this context. This will add to the theoretical evidence base, facilitating the development of effective, parsimonious and appropriate interventions that maximise the potential of therapy.

To our knowledge, this is the first review to consolidate evidence for the relationship between each ACT process, including the ACT-adjunct process of self-compassion, and distress in patients with cancer, providing a theoretical grounding of the mechanistic processes of ACT as applied to cancer. Previous reviews have investigated individual ACT constructs in cancer. A meta-analysis by Secinti et al. (2019) found acceptance had a small to moderate, significant negative association with distress (*r *= -0.31). In addition, a narrative systematic review on self-compassion in chronic physical illness (half of the samples with cancer patients), found moderate to large negative associations with anxiety and depression (Hughes et al., 2021). The current review addresses limitations to these previous reviews by including all ACT processes and self-compassion, reviewing grey literature to reduce publication bias and conducting robust meta-analysis. Conducting meta-analyses may also allow for moderators to be tested. Previous research is inconclusive for the clinical and demographic factors that may be associated with distress, however, these results give an indication of the moderators to test (Secinti et al., 2019). To understand if experiences are similar across cancer diagnoses (transdiagnostic), type of diagnosis would need to be tested as a moderator. However, individual research studies often recruit and combine a variety of cancer types in their analysis, with some consisting of very small samples of each cancer. Therefore, completing subgroup or moderator analysis based on cancer type may not be possible or appropriate. Moreover, as cancer samples are often split between early or advanced stage and extent of disease has been found to be associated with distress (Strong et al., 2007) it may be more appropriate to explore cancer stage as a moderator. In addition, exploring whether relationships between ACT processes and distress differ for age, time since diagnosis and gender, may be useful to help target interventions incorporating ACT processes. It has been found that women, younger age at diagnosis and those with a more recent diagnosis experience greater distress, although as previously suggested, results are often mixed (e.g., Carlson et al., 2019; Secinti et al., 2019).

Therefore, the primary objective of this review is to: identify the strength of associations between ACT processes (including the ACT adjunct process of self-compassion) and distress (e.g., depression, anxiety, emotional wellbeing) in patients with cancer using meta-analyses where possible and narrative review where there are insufficient studies for meta-analysis. The secondary objective is to: identify which ACT processes mediate distress outcomes in ACT interventions for patients with cancer. The review will also explore stage of cancer, time since diagnosis, gender and age as moderators of the relationship between ACT processes and distress.

## 2. Methods

This systematic review and meta-analysis have been conducted in line with PRISMA 2020 (Page et al., 2021b) and JARS-Quant for reporting meta-analysis guidelines (Appelbaum et al., 2018). The review is registered on PROSPERO, CRD42020166458 version 4.

### 2.1. Eligibility criteria

Studies were included if they conducted research with adults diagnosed with cancer, not at end of life, and reported associations between at least one ACT-related process and a distress outcome. Outcomes and processes must have been measured using validated measures that score the whole process (subscales of constructs of processes were not included, e.g., facets of present moment awareness measures; see supplementary materials, Table S1). Observational designs were eligible, as well as clinical trials that either analysed baseline data or reported mediation analysis of an ACT process on a distress outcome in a Randomised Controlled Trial (RCT). See Table 1 for further details on eligibility criteria.

**Table 1.** Inclusion and exclusion criteria for the studies in the review.

	Inclusion criteria	Exclusion criteria
Population	Adult patients (18+) with an adult diagnosis of cancer (any type/stage), treated with curative intent or palliative care (not end of life).	Patients under 18 years old, those without a diagnosis of cancer or adults who received a diagnosis as a child and/or those receiving end of life care (life expectancy <6 months).
Exposure/ intervention (predictor variables)	Studies presenting statistical tests of associations between validated measures of processes of ACT (see eligible processes outlined in Table S1) and the outcome.	Measures not validated. See Table S1 for further information regarding definitions of ACT processes included/excluded.
Outcome	Validated measures of distress (anxiety, depression, psychological/emotional distress, mood).	Measures of distress not validated. Studies that do not measure distress.
Study Design	Observational designs (i.e., cross sectional, prospective, cohort, baseline RCT) reporting bivariate relationships or multivariate models between the intervention variables and outcomes above. Additionally, RCTs for ACT based interventions if they explore ACT mediators on distress outcomes.	ACT RCTs that do not analyse mediators/processes of change, or do not analyse baseline data. Qualitative studies.
Other	Studies can use primary or secondary data, be conducted in any country, but must be published in English.	Studies not in English and where full texts cannot be accessed.

Note*: ACT = Acceptance and commitment therapy; RCT = Randomised Controlled Trial*

### 2.2. Information sources

Six electronic databases were searched: OVID (PsychInfo, MedLine & Embase), CINAHL, Web of Science and Cochrane library (CENTRAL); as well as five grey literature sources: SSRN, OpenGrey, WorldCat Dissertations and Theses, EThOS, Health Management Information Centre (HMIC) Ovid; with no date restrictions. The search was run between 28/02/2022 and 02/03/2022. The reference lists of included studies and other relevant reviews were hand searched.

### 2.3. Search strategy and selection process

The search strategy for all databases is available in supplementary materials (Table S2). Author SF managed records using EndNote version X9.3. The selection of eligible studies followed the PRISMA methodology (Page et al., 2021b). Duplicates were removed using the deduplication tool on EndNote and then by hand reviewing. Author SF independently screened all titles and abstracts using the PICOS criteria (stage one). KN and FM independently screened 100% between them. Studies deemed ineligible at this stage were removed, and the remainder moved to stage two screening. In stage two, full-text versions of all papers were retrieved and screened by SF using a predefined screening table in MS Excel. Two independent reviewers (KF and FM) also screened 79% of full-text papers. Cohen’s Kappa scores were calculated to determine interrater reliability and showed substantial agreement at stage one (k = 0.65–0.73) and almost perfect agreement at stage 2 (k = 0.71–0.84). Disagreements were discussed and resolved with the supervising corresponding author (LH).

### 2.4. Data items and collection process

A predefined data extraction table in MS Excel was piloted on three papers, and study location and ethnicity were added. Data extraction followed the PICOS criteria and included: study location, sample size, the proportion of males/females, mean age of the sample, ethnicity, diagnosis, time since diagnosis, treatment, study design, ACT process and measurement, outcome and measurement, means and standard deviations of the primary outcomes, type of analysis conducted and results (effect estimates and precision where reported). Data were grouped into each ACT process in the data extraction table (*note*: some studies tested more than one process). Author SF extracted the data, and two independent reviewers (IT and FM) cross-checked 70% of papers for extraction errors, including all meta-analysis data. Four authors were contacted to correct errors in published papers or to provide data where missing (i.e., from supplementary materials not publicly accessible), and two replied (see * on Table S5 and S6).

### 2.5. Risk of bias assessments

Risk of bias was assessed in all studies using a checklist adapted from ROBINS-E (Morgan et al., 2017) and a checklist used by Pasma et al. (2013) following guidelines on areas of bias (Dekkers et al., 2019; Sanderson et al., 2007). This assessment was developed as it is recommended that a risk of bias assessment checklist is suitable for the specific studies included in the review (Dekkers et al., 2019), in this case observational. No RCTs were included so an additional risk of bias tool was not needed. Author SF assessed each study using a checklist of up to 11 elements including the assessment of selection bias, response bias, attrition bias for longitudinal studies, bias due to missing data and analysis of confounders. Low, moderate or high risk and overall bias was stated (see Table S8). Author FM independently assessed 50% with moderate agreement (k = 0.55) and discrepancies were discussed until agreement. The GRADE tool (Guyatt et al., 2008) to assess bias across studies was used, providing an overall quality of evidence rating per process and outcome, considering the consistency of results, precision, publication and reporting bias. This was based on each meta-analysis. Outcomes for each process started at ‘low quality of evidence’ due to their observational nature and were downgraded if they scored ‘serious’ for any criteria. Quality could be upgraded if they had a large effect (Guyatt et al., 2008). Indirectness was not assessed as all papers were of the population of interest. Publication bias was assessed using funnel plots if there was a minimum of 10 studies (Page et al., 2021a).

### 2.6. Effect measures

Correlations and regression coefficients were the most reported measures of effect size. Three studies reported tests of mean difference, whilst another reported a Mann Whitney U test. No other effect measures were used. In narrative synthesis, effect estimates are presented with the direction of effect and summarised based on how many studies show estimates in the same direction. Due to the lack of studies reporting regression analyses and high heterogeneity between those that did, all regression data are narratively synthesised. No relevant RCTs were found reporting mediation or process analysis.

### 2.7. Synthesis methods

Strong correlations are generally found between distress measures in cancer implying a similar construct is being measured (Pandey et al., 2007; Rabkin et al., 2009). Therefore as in similar meta-analyses of distress outcomes (e.g., Winger et al., 2016), measures including anxiety, depression, distress, negative affect and emotional stress were combined where possible and average correlations calculated if they were comparable in terms of definition of measurement and where correlations with measures of individual ACT processes in each study were similar. Correlations were Fisher's Z-transformed, the mean calculated and then back transformed to an *r* correlation using R Studio code (R Core Team., 2020). ACT process measures were also synthesised where measurements were comparable in definition (e.g., values importance and values success were not considered comparable definitions of values). A meta-analysis was run for each process (or the overall process of psychological flexibility) and distress if at least *k *= 5 studies used the same type of correlations e.g., Pearson’s *r* (recommendations range from 2-8 as minimum; Jackson & Turner, 2017; Rhodes & Smith, 2006; Ryan, 2016). The random effects model considered between-study differences and sampling variation and was used with the Restricted Maximum Likelihood (REML) estimator for tau squared (variance between studies) due to the continuous outcome data (Harrer et al., 2021; Langan et al., 2019). The ‘metacor’ package on R Studio (Laliberte, 2019) used Fisher’s Z-transformation to estimate pooled correlations and reports 95% confidence intervals (CI; see code in supplementary materials S3). In line with Cohen (1988), *r *< 0.1 is interpreted as a trivial effect, *r *≥ 0.1 is interpreted as a small effect, *r *≥ 0.3 is a medium effect and *r* ≥ 0.5 is interpreted as a large effect. Heterogeneity was assessed using *I*^2^, which is the percentage of the variability in effect sizes (<40% low heterogeneity, 50-100% moderate to high heterogeneity; Deeks et al., 2022). To explore heterogeneity, as decided *a-priori*, moderator analysis was conducted with stage of diagnosis, rather than by cancer type due to the mixed cancer samples being included in many studies. Moderator analysis was conducted using the meta-regression function of metacor in R Studio and if the minimum number of studies was *k *= 10 (Borenstein et al., 2009). Exploratory post-hoc moderator analyses were conducted on age, gender and time since diagnosis where there were sufficient studies, as these factors have been found to be associated with distress and ACT processes in previous research (e.g., Carlson et al., 2019; Secinti et al., 2019). Where there was insufficient data to complete meta-analysis for individual processes, the data are narratively synthesised, reporting correlations and standardised beta coefficients (or unstandardised if standardised betas were not reported). Additional data for processes that were meta-analysed but where the data could not be reasonably incorporated, have been narratively synthesised and are available in supplementary materials to provide a complete summary of available data.

## 3. Results

### 3.1. Study selection

**Figure 1.**
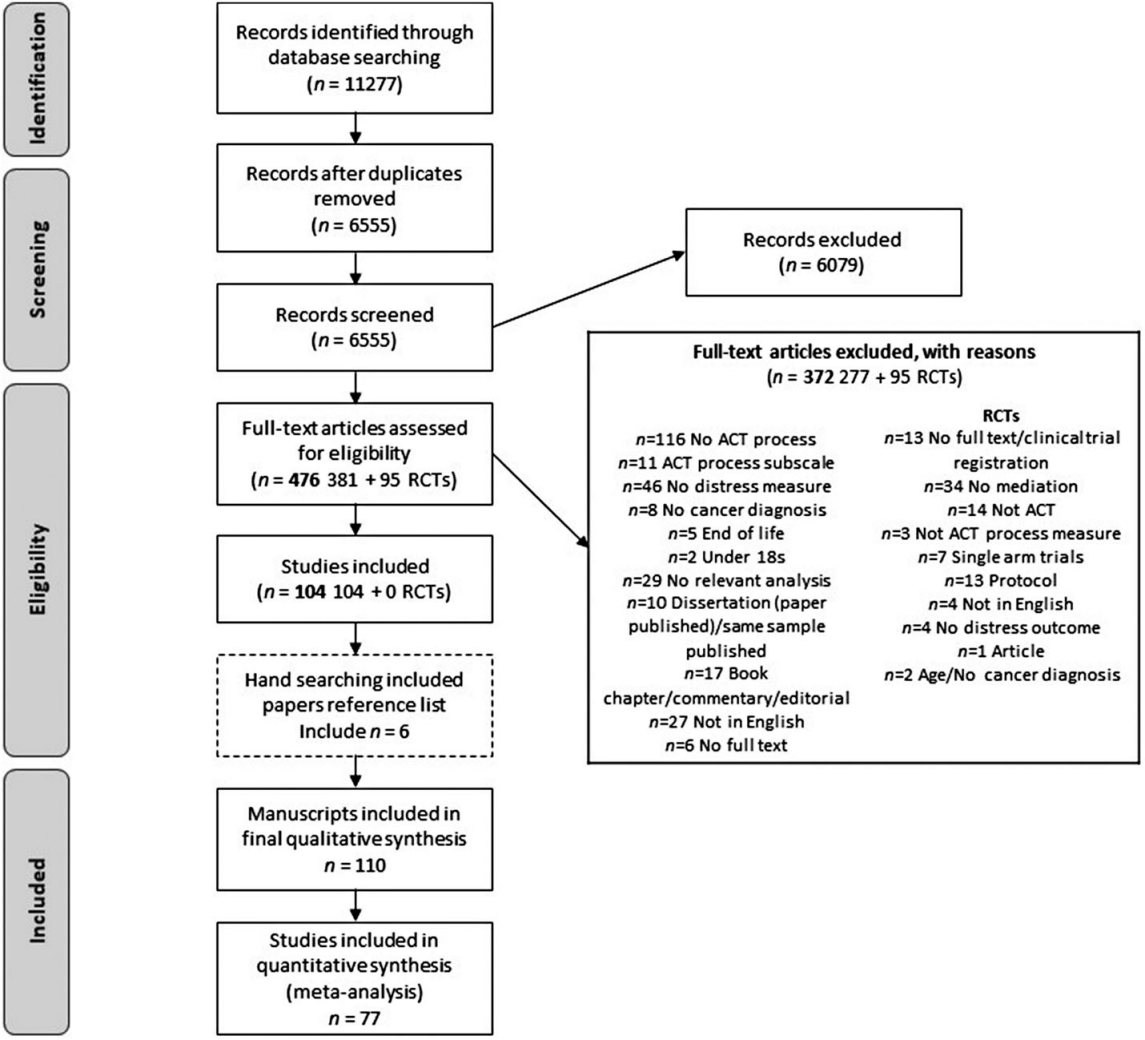
PRISMA flow diagram of included studies.

Figure 1 displays the search results and the number of papers screened at each stage. After duplicates were removed, 6555 were screened at stage one based on titles and abstracts, and 477 full texts were screened at stage two. One hundred and ten manuscripts (108 studies) were included in this systematic review, with 77 included in at least one meta-analysis. Most full texts excluded were due to studies not having distress as an outcome (*n *= 46), no ACT process (*n *= 116), not reporting relevant analyses (*n *= 29) or not being published in English (*n *= 27). The majority of RCTs excluded were due to not completing mediation analysis (*n *= 34) and, if they did, not consisting of an ACT intervention or being a single-arm trial (*n *= 21).

### 3.2. Study characteristics

Study characteristics and a reference list of all included studies can be found in supplementary materials (Table S4). Studies are listed in alphabetical and numerical order and are therefore referenced with this corresponding number throughout this review. In the 110 studies, sample sizes ranged from 14-922 and included a total of 17,195 participants (108 samples). The present review included 18 grey literature studies. The majority were carried out in the USA/Canada (*n *= 44), with 30 across Europe, 11 in China and eight in Australia/New Zealand. The remaining studies were conducted in Brazil, Jordan, Nigeria, Egypt, Japan, Taiwan, Singapore, Iran, Israel and Malaysia. The majority used either a breast cancer sample (*n *= 41) or a mixed cancer sample (*n *= 36). Seventy-one studies reported depression as an outcome, with 54 studies reporting anxiety, and 39 reporting distress. Some studies reported emotional/psychological wellbeing (*n *= 13), emotional stress/dysfunction (*n *= 12) and negative affect (*n *= 6) as outcomes.

### 3.3. Risk of bias within studies

The risk of bias scoring is available in supplementary materials (Table S8). The methodological quality of studies was generally good, with a low risk of bias overall score for 68% of studies. Almost all studies reported *a priori* outcomes and/or hypotheses to reduce bias in reported outcomes, used validated process and outcome measures and reported significance values, scoring consistently low for risk of bias. Twenty-five per cent of studies failed to report detailed recruitment processes and received ‘unclear’ for risk. These details are essential to allow replication and would give a clearer indication of potential selection bias in recruitment. Response rates were also unclear, meaning risk of bias could not be assessed (53%) or were scored as moderate/high (22%), indicating potential non-response bias and difficulties with assessing representation of samples. Strategies for dealing with missing data (58%) and *a priori* sample size calculations (41%) were often not reported. Studies often failed to assess and/or control for confounders leading to potential biases in the estimates reported.

### 3.4. Results of meta-analyses

**Figure 2.**
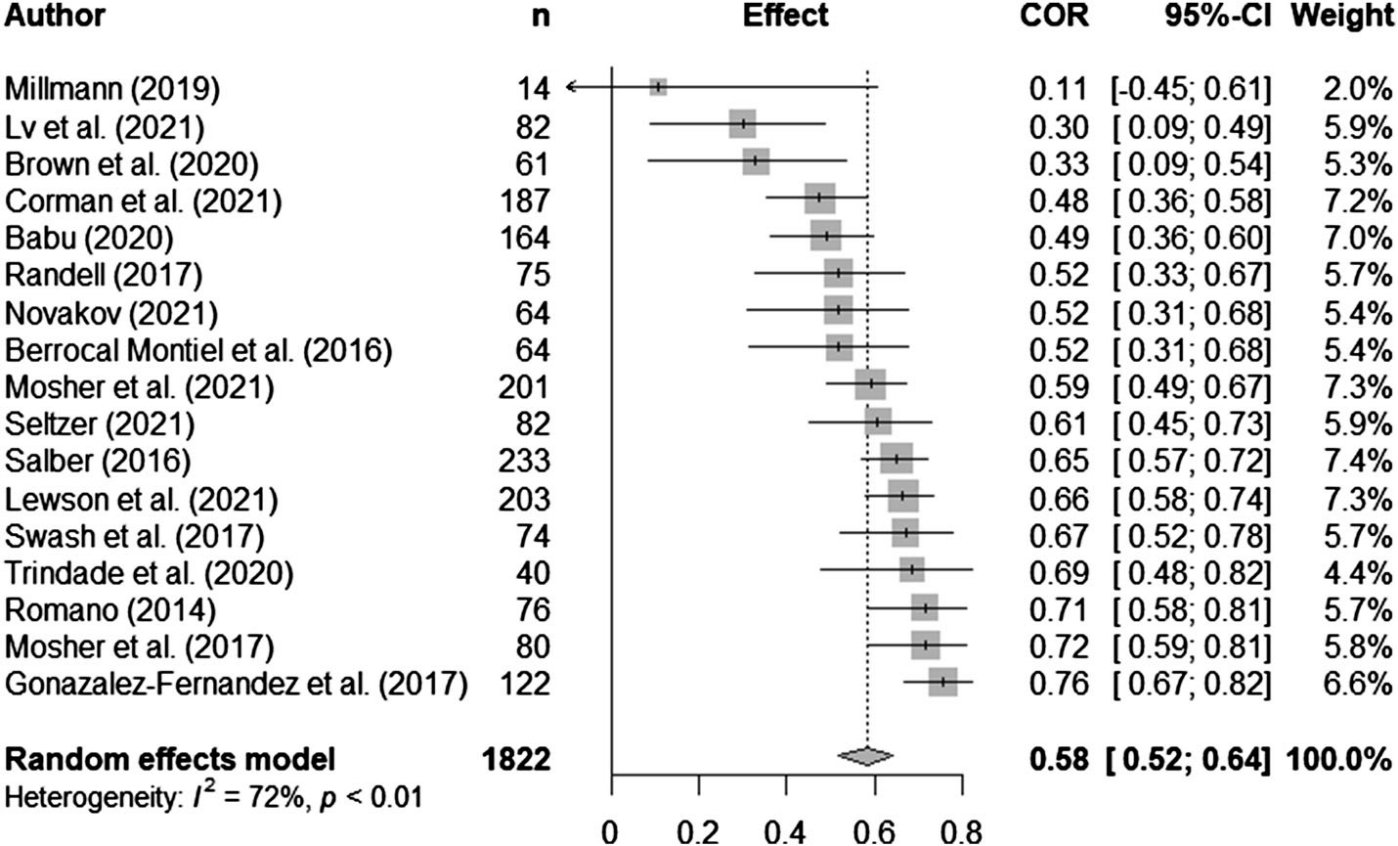
Meta-analysis for experiential avoidance and distress outcomes.

**Figure 3.**
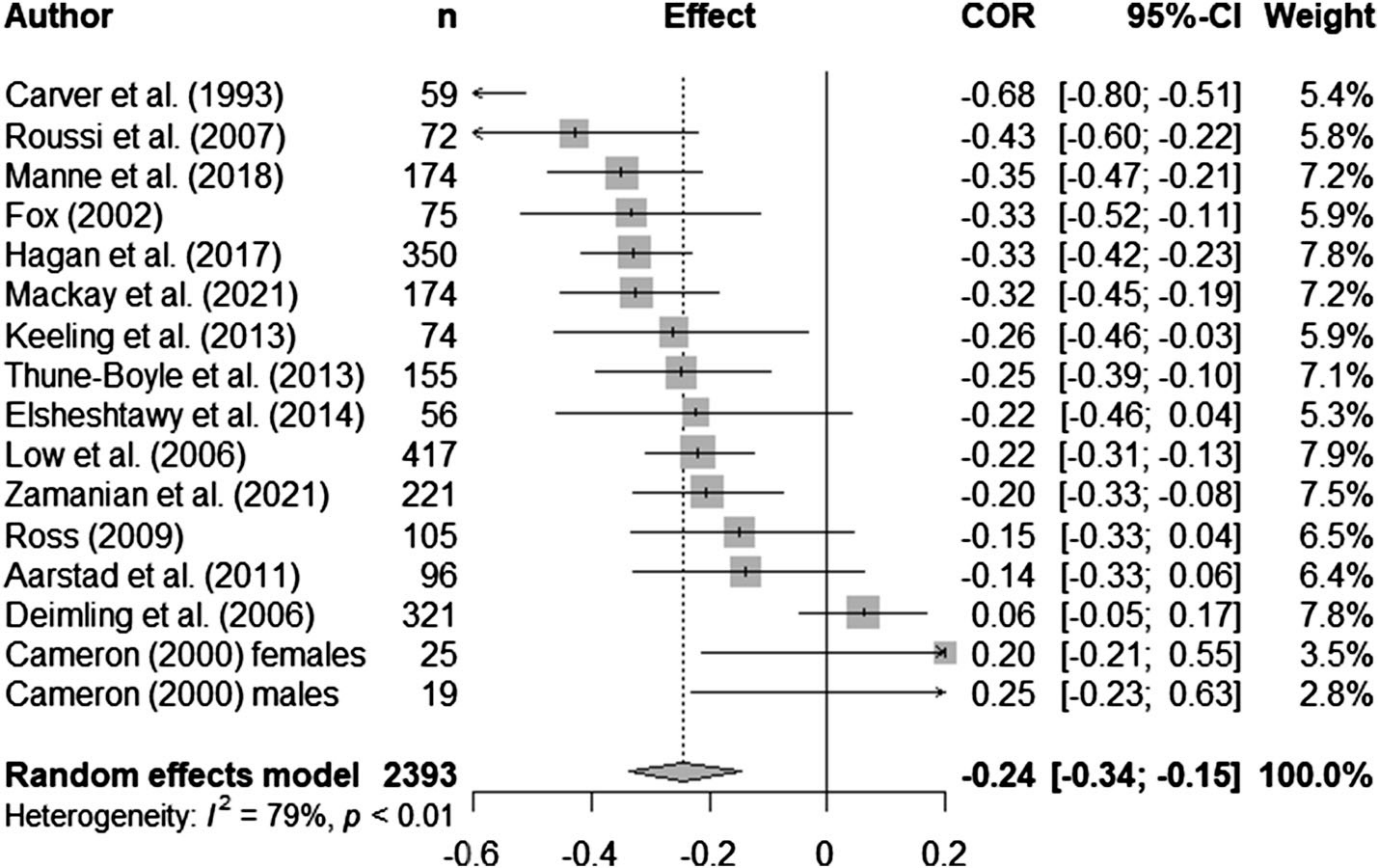
Meta-analysis for acceptance and distress outcomes.

**Figure 4.**
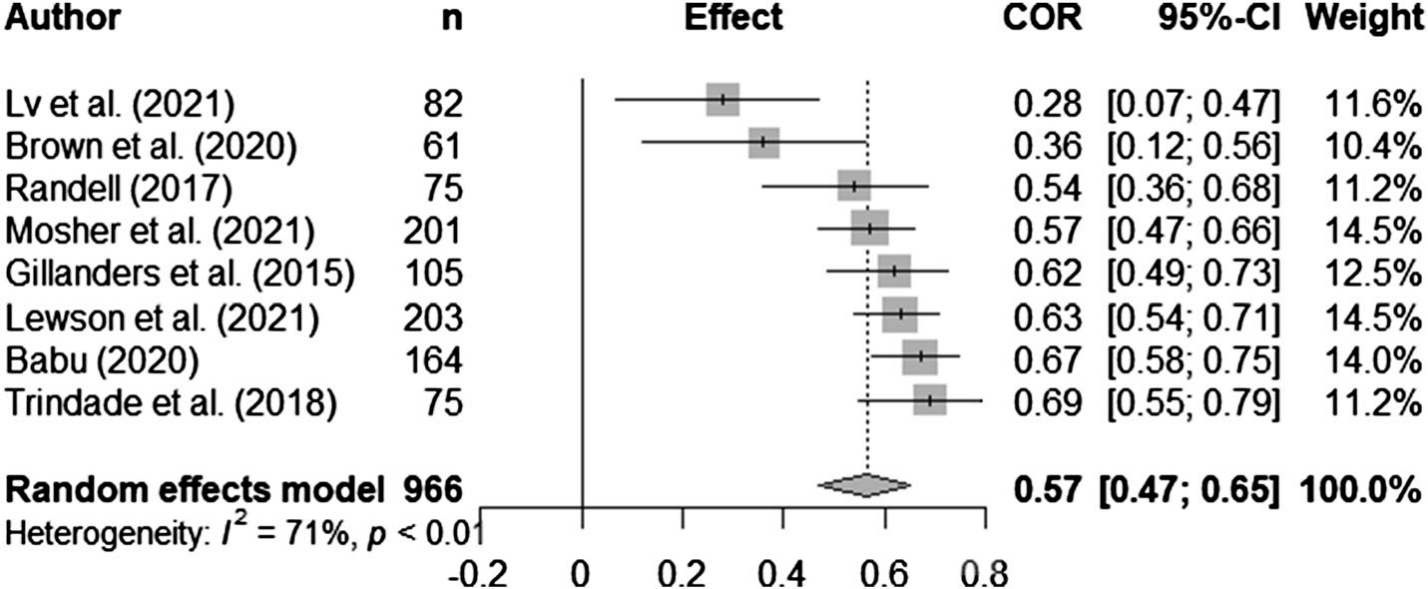
Meta-analysis for cognitive fusion and distress outcomes.

**Figure 5.**
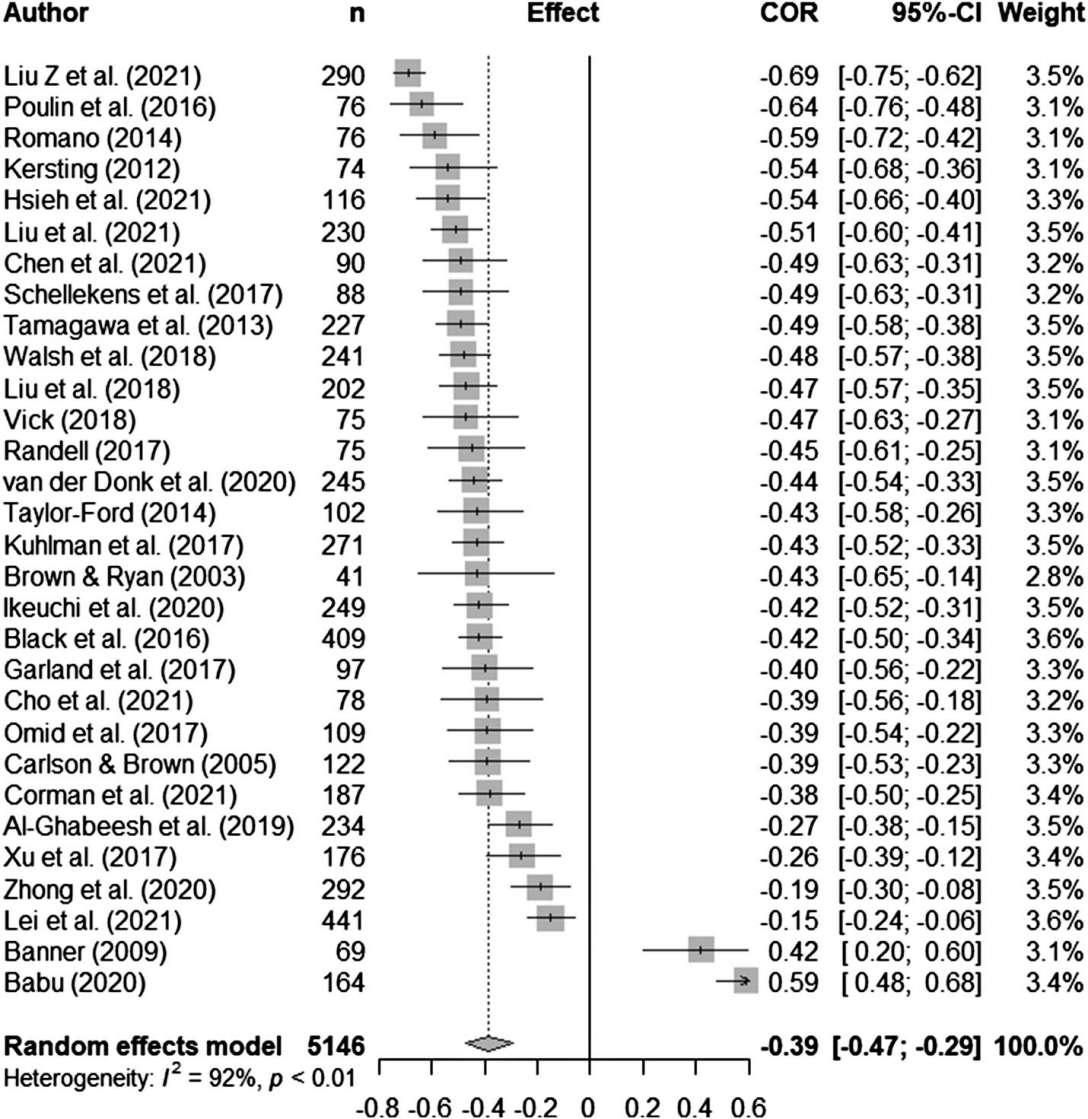
Meta-analysis for present moment awareness and distress outcomes.

**Figure 6.**
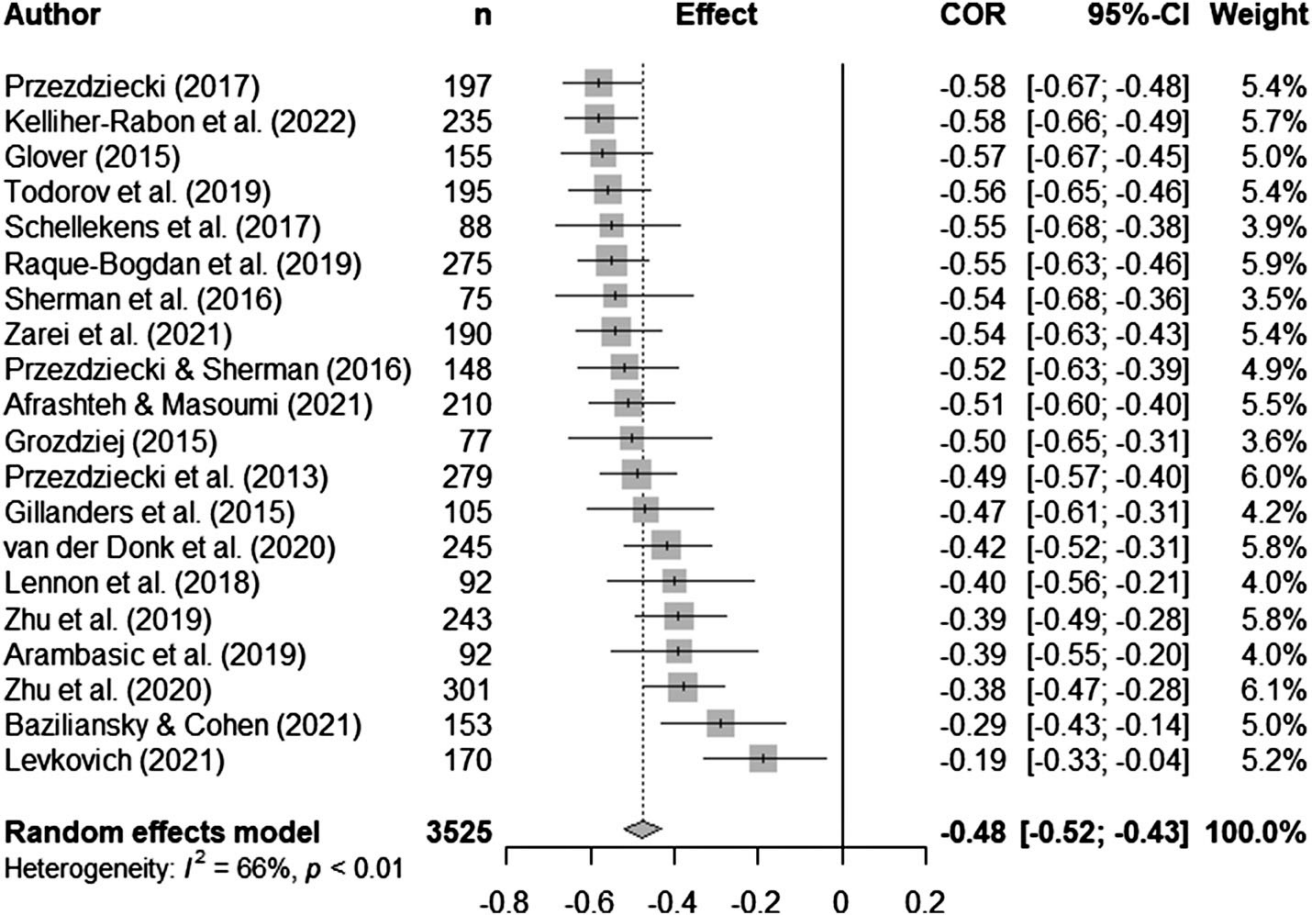
Meta-analysis for self-compassion and distress outcomes.

Syntheses are structured by ACT process. Five meta-analyses (pooled estimates are displayed in Table 2) were conducted between distress and experiential avoidance, acceptance, cognitive fusion, present moment awareness, and self-compassion (raw data are available in supplementary materials, Table S5 and Figures 2–6 display all forest plots). Narrative synthesis on data that could not be incorporated into the following meta-analyses are presented in supplementary materials S7 to provide a complete overview of available data including regression data controlling for covariates.

**Table 2.** Display of pooled estimates for random effects models.

ACT process and distress	*n*	*k*	*r_pooled_*	95% CI	I^2^
Experiential avoidance	1822	17	0.58	0.52, 0.64	72.0%
Acceptance	2393	16	-0.24	-0.34, -0.15	78.8%
Cognitive fusion	966	8	0.57	0.47, 0.65	70.5%
Present moment awareness	5146	30	-0.39	-0.47, -0.29	92.5%
Self-compassion	3525	20	-0.48	-0.52, -0.43	65.7%

Note: *n* = overall sample size; *k* = number of effect sizes included; *r*_pooled_ = pooled correlation; CI = confidence interval; I^2^ = test of heterogeneity.

#### 3.4.1. Experiential avoidance

Twenty-five studies explored experiential avoidance and distress outcomes: 18 cross-sectional and 7 longitudinal. Most studies were mixed cancer (*n *= 10) or breast cancer (*n *= 6), with five haematological, one thyroid, one prostate, one colorectal and one gynaecological sample. Sample sizes varied from 14-922. Seventeen studies were included in the meta-analysis. There was a significant pooled effect with a strong positive correlation between experiential avoidance and distress (*r_pooled _*= 0.58, 95% CI 0.52, 0.64). Heterogeneity was high (*I*^2 ^= 72%; see Figure 2 for forest plot).

#### 3.4.2. Acceptance

Thirty-three studies explored acceptance (measured by the COPE or brief COPE; Carver, 1997; Carver et al., 1989), self-acceptance, emotional acceptance and pain acceptance, in breast cancer (*n *= 14), mixed cancer samples (*n *= 10), gynaecological (*n *= 3), and one study each in head and neck, brain, gastrointestinal, blood, colorectal and melanoma. Sample sizes ranged from 14 to 460, with 27 cross-sectional (including one baseline RCT), and six longitudinal studies. There were sufficient data to run a random effects meta-analysis for acceptance and distress. The pooled correlation was significant, although with a small effect (*r_pooled_* = -0.24, 95% CI -0.34, -0.15, *k *= 16; see Figure 3 for forest plot). Only studies using the COPE measure were included in the meta-analysis. Heterogeneity was moderately high (*I*^2 ^= 78.8%). Five studies reported non-significant, small effects, with two in the opposite direction than expected, one a grey literature study (17,26).

#### 3.4.3. Cognitive fusion

Eight cross-sectional studies explored cognitive fusion with distress outcomes with samples ranging from 61 to 203. Six were mixed samples (8,15,32,52,62,75), one was a breast cancer sample (96), and one was thyroid cancer (57). There were sufficient data to run a random effects meta-analysis for cognitive fusion and distress. The pooled correlation was significant, with a large positive effect (*r_pooled_* = 0.57, 95% CI 0.47, 0.65, *k *= 8). Heterogeneity was high (*I*^2^ = 71%; see Figure 4 for forest plot).

#### 3.4.4. Present moment awareness

Thirty-four studies explored present moment awareness (using mindfulness measures) and distress in various cancer samples (mixed *n *= 12, breast *n *= 9, gastrointestinal *n *= 6, lung *n *= 4, blood *n *= 2 and prostate *n *= 1). Sample sizes varied from 41 to 441. Thirty-two were cross-sectional (including one baseline RCT), and two were longitudinal. There were sufficient data to run a random-effects meta-analysis using correlations for total mindfulness measure scores with distress outcomes. There was a significant medium pooled effect for present moment awareness and distress (*r_pooled_* = -0.39, 95% CI -0.47, -0.29, *k *= 30). Heterogeneity was very high (*I^2^* 92.5%; see Figure 5 for forest plot). Two grey literature studies report results in the opposite direction to the other studies (8,9).

#### 3.4.5. Self-compassion

Twenty-four studies tested self-compassion and distress outcomes in mostly breast cancer (*n *= 10) and mixed samples (*n *= 8), with two samples each for lung and colorectal cancer and one sample each for prostate and gastrointestinal cancers. Sample sizes varied from 58-301. Twenty-one studies were cross-sectional and three longitudinal. There were sufficient data from twenty studies to run a meta-analysis. The random effects meta-analyses showed there was a significant medium pooled effect with self-compassion inversely correlated with distress (*r_pooled _*= -0.48, 95% CI -0.52, -0.43, *k *= 20). There was moderate heterogeneity (*I*^2 ^= 65.7%; see Figure 6 for forest plot).

### 3.5. Moderator analysis

Heterogeneity was present in all five meta-analyses. Moderator analysis was conducted with stage of diagnosis. There were less than 10 studies for the cognitive fusion meta-analysis, so this was excluded from all further analysis. Stage of diagnosis did not significantly moderate any of the relationships between processes and distress (see Table 3). For exploratory moderator analysis, age, gender and time since diagnosis were analysed (there were less than 10 studies for both experiential avoidance and acceptance with the moderator ‘time since diagnosis’ so these were not included). There were no moderator effects on the relationship between any of the processes and distress (see Table 3).

**Table 3.** Moderator analysis.

Process and distress	Moderator	*k*	*n*	I^2^	R^2^	B	SE	95% CI
Experiential avoidance	Stage	13	1410	62.67%	0.00%	0.000	0.001	-0.002, 0.003
	Age	17	1822	68.85%	20.94%	0.011	0.011	-0.002, 0.021
	Gender	17	1822	69.80%	17.29%	0.002	0.002	-0.001, 0.010
Acceptance	Stage	13	2025	85.47%	0.00%	-0.001	0.002	-0.005, 0.004
	Age	16	2393	79.46%	6.71%	0.011	0.011	-0.004, 0.022
	Gender	15	2172	79.68%	13.16%	-0.003	0.002	-0.011, 0.001
Present moment awareness	Stage	28	4850	93.85%	0.00%	0.000	0.002	-0.004, 0.004
	Age	26	4162	92.87%	2.29%	-0.013	0.011	-0.034, 0.010
	Gender	30	5146	93.34%	0.00%	-0.000	0.002	-0.004, 0.004
	Time since diagnosis	14	1940	87.56%	2.46%	-0.065	0.060	-0.173, 0.044
Self-compassion	Stage	13	2394	73.93%	0.00%	0.001	0.004	-0.007, 0.009
	Age	17	3035	70.56%	0.00%	0.002	0.005	-0.010, 0.011
	Gender	20	3525	66.09%	0.00%	-0.001	0.001	-0.003, 0.001
	Time since diagnosis	10	1703	58.27%	16.01%	-0.023	0.021	-0.064, 0.020

Note: *n* = overall sample size; *k* = number of effect sizes included; I^2^ = residual heterogeneity; R^2^ = amount of heterogeneity accounted for; B = estimate; SE = standard error; CI = confidence interval; time since diagnosis was not run as a moderator for experiential avoidance or acceptance and distress due to there being less than 10 eligible studies; no moderator analysis was run for cognitive fusion due to less than 10 studies overall.

### 3.6. Narrative synthesis of remaining processes

Meta-analyses could not be conducted on self-as-context, committed action, values and overall psychological flexibility due to the limited number of studies for each process, so a narrative synthesis was completed (details of extracted information and data are available in Table S6).

#### 3.6.1. Self-as-context

One dissertation explored self-as-context in a mixed cancer sample of 164 (8). There was a significant negative correlation between self-as-context and depression (*r* = -0.22); however, a non-significant association was found for anxiety (*r* = 0.10).

#### 3.6.2. Committed action and values

One cross-sectional study (75) was conducted with 75 mixed cancer participants. There were significant moderate-strong negative correlations between the engaged living scale (measuring committed action and values) and distress outcomes (*r* = -0.50 to -0.56). In regression analyses, engaged living was significantly associated with lower depression (β = -0.33), however, it was not significantly associated with lower anxiety.

#### 3.6.3 Committed action

Two studies (8,97) explored committed action with distress outcomes. These cross-sectional studies included breast cancer and mixed cancer sample (*n *= 82 and 164). In one study (97), there were significant, moderate negative correlations for committed action and distress outcomes (*r* = -0.46 to -0.53). However, in the grey literature study (8), associations in the opposite direction were found with committed action significantly positively associated with distress outcomes (*r *= 0.32 to 0.36).

#### 3.6.4. Values

Five cross-sectional studies (8,22,52,62,63) and one longitudinal study (47) explored values in four mixed and two breast cancer samples, with samples from 32-203. A meta-analysis was not conducted as values measures were not comparable in definition. Value progress, success and importance was negatively correlated with anxiety, depression and distress (8,22,52,62,63; *r *= -0.43 to -0.16), whilst value obstruction was positively associated with anxiety and depression (52,62,63; *r *= 0.61 to 0.66). In line with this, those who were anxious or depressed reported worse discrepancies between value importance and value attainment in different domains, at different time points compared to those who were not anxious or depressed (47). Another study found similar conclusions for values success in different value domains being positively correlated with emotional wellbeing (22; *r *= 0.34 to 0.50). However, greater commitment to family values was negatively correlated with emotional wellbeing (*r *= -0.29), and values success in romantic relationships was negatively associated with emotional wellbeing in females (22; β = -0.26), whilst controlling for avoidance.

#### 3.6.5. Overall psychological flexibility

Two cross-sectional studies (60,84) measured overall psychological flexibility, with samples ranging from 144 to 286, both consisting of prostate cancer. There were strong, negative associations for psychological flexibility and distress (*r *= -0.69 and -0.67), and psychological flexibility was significantly associated with lower distress when controlling for age, self-esteem and stoicism (β = -0.41) and fear of recurrence (β = -0.56).

### 3.7. Risk of bias across studies

The GRADE assessment was used to assess bias across study outcomes in meta-analyses (Guyatt et al., 2008). Overall, the quality of evidence was very low for three processes and low for two processes (see Table S9 in supplementary materials). The meta-analyses for acceptance and present moment awareness scored serious or very serious in more than one domain. All meta-analyses scored serious or very serious for inconsistency as heterogeneity scores were high. However, data were relatively precise with four out of five meta-analyse scoring not serious for imprecision and none were serious for reporting bias. For a summary of the assessment of narrative syntheses see supplementary materials S9.

## 4. Discussion

This review has been the first to quantify the direction and strength of relationships using meta-analysis between ACT processes, including the ACT-adjunct process of self-compassion, and distress in patients with cancer. Empirical research in this area has increased over recent years, with 26 studies published between 2020 and 2022 alone, making this systematic review and meta-analysis particularly timely to guide the direction and quality of future research. This review provides a comprehensive overview by including a total of 110 manuscripts. Meta-analyses revealed significant associations between ACT processes and distress outcomes for people with cancer, whereby higher scores on flexible processes (present moment awareness, acceptance, and self-compassion) are associated with lower distress, and higher scores on inflexible processes (experiential avoidance, cognitive fusion) are associated with higher distress. This aligns with previous separate reviews on acceptance, self-compassion, and distress in cancer and other physical health populations (Hughes et al., 2021; Secinti et al., 2019). Narrative syntheses indicate that less investigated processes of values and overall psychological flexibility show promising moderate to strong associations with distress in the directions expected. However, only three studies explored self-as-context and committed action, with mixed results. This review provides empirical support for the theorised relationships between distress in patients with cancer and the flexible and inflexible processes depicted in the ACT model, with directions of association as suggested by the model.

Meta-analyses revealed large significant positive relationships between distress and experiential avoidance and distress and cognitive fusion indicating that greater inflexibility is associated with greater levels of distress. Self-compassion had a moderate negative pooled correlation with distress while present moment awareness had a slightly weaker, moderate negative pooled correlation with distress suggesting greater levels of these flexible processes are associated with lower levels of distress. The weakest relationship was observed between acceptance and distress. Data from the majority of cross-sectional regression analyses whilst controlling for covariates support these results, providing additional support for the relationships between these ACT processes and distress, over and above some demographic/clinical and/or psychological variables. However, these data are limited for cognitive fusion and only experiential avoidance had data from longitudinal studies to support this process predicting distress over time. Results were however across a variety of cancer samples. Similar relationships have been found in other physical health populations, such as inflammatory bowel disease, multiple sclerosis and diabetes (Hughes et al., 2021; Jedel et al., 2013; Pagnini et al., 2019; Trindade et al., 2016, 2018; Valvano et al., 2016). In line with the inflexible processes in ACT, fusing with or avoiding difficult emotions, thoughts or memories was associated with distress. It is understandable that people with cancer and other physical health conditions may have particularly difficult emotions and thoughts around future recurrence/relapse, mortality, ongoing treatment, and memories from past treatment; however, responding to these in an inflexible way may be problematic. Conversely, the flexible processes of being present and, especially being self-compassionate in facing difficulties, were associated with lower distress. The transdiagnostic evidence across different physical health conditions for these relationships, from previous research as well as this review, supports the universal processes approach of ACT which is designed to be a unified process-driven model to reduce suffering and improve wellbeing (Hayes, 2019). The unified, process-based model can help to promote effective treatment strategies across conditions that experience similar unpleasant sensations and uncertainty. This transdiagnostic approach may broaden the access and availability of treatment, potentially improving outcomes for patients with cancer.

The smallest relationship was found between acceptance and distress, with a weak negative pooled correlation observed. Although a weak relationship was found, this is in the direction expected with greater acceptance associated with lower levels of distress. Some cross-sectional regression data controlling for covariates supported this relationship, however there was a lack of longitudinal studies conducted. According to ACT, the willingness to experience difficult emotions and thoughts should be associated with better psychological outcomes (Hayes et al., 2006). The small effect found in this review may be due to measurements of acceptance being developed from different theoretical models and definitions, such that acceptance was not adequately operationally defined to ensure consistency across participants completing the measures (McAndrews et al., 2019). For example, the COPE inventory (Carver, 1997; Carver et al., 1989) was included in this review as a measure of acceptance as it has an active/process stance which ACT proposes, rather than acceptance as an end point or as resignation which is incongruent with the ACT model (Hayes et al., 2006; Hulbert-Williams & Storey, 2016). However, due to the variation of definitions of acceptance (McAndrews et al., 2019), the COPE may be interpreted by participants as coping with the diagnosis label itself or another stressful life event, rather than the ongoing process of living with cancer. It may therefore fail to capture the experiential, ACT-based process like the emotional acceptance scale (Politi et al., 2007). Whilst data were insufficient for meta-analysis using this subscale, the narrative synthesis supports this, with moderate relationships found with distress. The results are similar to a previous review (Secinti et al., 2019) which used an integrated model of acceptance, combining all coping measures, ACT measures and acceptance of illness measures, not necessarily all congruent with the ACT process model stance. The fact that small to moderate effects were found suggests neither method is adequately measuring the core ACT process of acceptance. Combining measures which are conceptually distinct from one another may also be unhelpful when deciding on definitions and models when developing interventions. Clear evidence for theories is needed to drive intervention development and specifically inclusion of processes of change. Similar to recommendations by Secinti et al. (2019), the current results suggest that the development of a measure of acceptance which encapsulates more of the cancer experience and in an ACT congruent way is needed, providing a clearer definition and conceptualisation for future research.

Although not a central tenet of the psychological flexibility model, self-compassion fits with the ACT approach in bringing awareness to suffering and distress as shared aspects of the human experience, to be acknowledged without self-criticism, a potentially pertinent process for those with cancer. Therefore, although self-compassion is interwoven throughout ACT in practice and training and seen as a potential mechanism of change, it is not adequately conceptualised within the model (Carvalho et al., 2022; Neff & Tirch, 2013). The lack of formal inclusion in the model poses the question of how this process is utilised in ACT therapy, how it is developed if there is no clear theoretical underpinning, and whether it depends on the therapists’ skill. It also gives rise to uncertainty regarding the actual core processes of ACT (Arch et al., 2022). Although self-compassion is an inherent value in ACT (Luoma & Platt, 2015), Neff and Tirch (2013) suggest explicit self-compassion exercises are needed to develop the process which can act as a mechanism of change for outcomes. The results of this review suggest that there is a moderate negative association between self-compassion and distress, such that patients with higher levels of self-compassion report less distress. This, coupled with the findings of Hughes et al. (2021) and evidence that self-compassion interventions reduce distress (Kılıç et al., 2021), suggests that a more formal description and conceptualisation of self-compassion should be incorporated into the ACT model to promote good intervention development and delivery.

Analyses revealed no significant moderators for any of the relationships between these ACT processes and distress. This suggests the relationships between the processes and distress did not differ for different stages of diagnosis, age, gender or time since diagnosis. Type of cancer was not tested as a moderator due to the mixed cancer diagnoses within samples. Time since diagnosis and breakdown of stages was often poorly reported so these results should be interpreted with caution and may need further research to confirm these relationships. However, previous research is generally mixed in finding clinical and demographic factors associated with these processes and outcomes such as distress, indicating that this may remain true transdiagnostically (Secinti et al., 2019). It may be that these factors are not key moderators and therefore the relationships between processes and distress do not differ throughout the cancer journey or for individuals of different ages, stages and gender. Therefore, interventions based on these processes may be suitable for a broad population of cancer patients, potentially making implementation easier.

Few studies investigated committed action, values, self-as-context and overall psychological flexibility, so results must be interpreted with caution. A narrative synthesis of the available data identified some associations with distress in the expected directions, with these flexible processes associated with lower distress in cross-sectional studies. It is surprising that there are so few studies providing an evidence-base for values in cancer as this is often reported as key content in ACT interventions for cancer patients (e.g., Mathew et al., 2021). However, these processes are consistently understudied in ACT literature (Arch et al., 2022). Values can be a difficult construct to measure as they are ongoing and dynamic in ACT (Barrett et al., 2019), and scales vary in their measurement with obstruction, progress and/or importance used and the process of values clarity rarely measured (McLoughlin & Roche, 2022). The importance of different values may understandably change over time and throughout the cancer journey, so there is potential to explore this process at different points (Lampic et al., 2002) and may be important in the development of tailored ACT interventions. Both values and self-as-context are further examples of processes that are either difficult to define or have variations in their definitions which can introduce uncertainty in how they are perceived and understood (Barrett et al., 2019; Zettle et al., 2018). It is, therefore, hard to determine the saliency of these constructs in the model for cancer.

The ACT theory and framework have been used to inform psychological interventions for distress in those with cancer. Still, the key mechanisms through which the intervention is hypothesised to work are often not identified in this population. Process-Based Therapy (PBT), a more recent intervention approach proposed by Hayes and Hofmann (2018), suggests evidence-based processes of change should be identified and used rather than a traditional protocolised approach (Hayes et al., 2020). They suggest this approach should be at an individual, personalised level, for a specific outcome (such as distress) and for appropriate contexts such as cancer, which could increase the efficacy and effectiveness of interventions. The current reviews' results support this approach as it provides evidence for key mechanisms associated with distress which could be developed as briefer, more targeted process-based interventions or be flexibly incorporated with other theoretically aligned interventions. In addition, where appropriate, theories themselves should be tested and refined or adapted in their application to a specific population considering the empirical evidence as it emerges (Levin et al., 2012). This review has highlighted key relationships between flexible and inflexible processes and distress in cancer providing evidence for theory development to inform more successful process-based interventions for those with cancer. However, identifying the lack of evidence for certain key processes depicted in the model, highlights a limitation to the model when applied to cancer and proposes the pathway for future research.

### 4.1. Implications of current findings

Understanding the key evidence-based mechanisms from the ACT model means intervention development can be guided by which variables should be targeted or emphasised. Successful psychological interventions targeting key mechanisms for managing distress in cancer have potential to reduce costs on multiple levels, including for individuals, medical systems and wider health networks (Chatterton et al., 2016). On an individual level, distress is linked to poor adherence to ongoing treatment and medical recommendations, increased recurrence and poorer survival rates, so addressing this outcome is imperative (DiMatteo & Haskard-Zolnierek, 2011; Fangand & Schnoll, 2002). In addition, identifying processes that may be predictors of distress could inform screening strategies to identify those at risk of developing distress, encouraging suitable early intervention (Hulbert-Williams & Storey, 2016). It could be particularly important to identify experiential avoidance and/or cognitive fusion at diagnosis as these processes had the strongest associations with distress across the different cancer samples.

### 4.2. Strengths and limitations

There are several limitations to this review which need to be considered. Firstly, most studies were cross-sectional, meaning only meta-analyses of correlations could be conducted, limiting the ability to make assumptions regarding causality. The longitudinal data available were very limited and provided mixed results. Second, the meta-analyses and GRADE assessment identified significant heterogeneity across studies. This could be due to variations in process and outcome measures used as well as limits to the number of studies and sample sizes included. Due to the nature of the GRADE assessment depicting observational studies as low-quality evidence, all meta-analyses were scored as low or very low-quality, implying that further data are likely to be substantially different from the estimated results (Guyatt et al., 2008). However, considering the number of studies included in the meta-analyses, the precision and low reporting bias, further data are likely to support the overall conclusions of the meta-analyses (particularly in the case of the directions of effects) in this review. Excluding patients facing end of life was one means by which heterogeneity was sought to be reduced. This review attempted to reduce publication bias by including grey literature, by using key terms for searching and conducting a wide search with several electronic databases. However, some relevant articles may not have been identified, and conference abstracts were not included. Some studies had non-significant results or contradictions to expected results and some of these were grey literature, studies with small sample sizes and/or those of low quality. It is important that more high-quality research is published to avoid potential publication bias. The review is also susceptible to language bias as only English-language papers were included. Several areas of study methodology indicated an unknown or high risk of bias. Despite these limitations, the current review is the first to consolidate data on all ACT processes and the ACT-adjunct process of self-compassion and their association with distress in cancer.

There are also wider limitations associated with this field of research. The AAQ (Bond et al., 2011; Hayes et al., 2004) was used in 21 studies to measure experiential avoidance, however, the measure has been widely criticised for its strong correlations with distress due to measurement overlap (Tyndall et al., 2019). This was demonstrated in this review; however, overall results were supported by data from the alternative measures. As there were insufficient studies, formal subgroup analysis could not be conducted, which is an important consideration for future research. Findings should therefore be interpreted with some caution and alternative measures of experiential avoidance used when measuring associations with distress. Most studies in this review were cross-sectional and it is therefore recommended that more longitudinal studies are conducted to explore which processes predict distress over time as well as identifying the stability of these relationships. A diagnosis of cancer may require continual self-management and adjustment to changes in treatment and status. Understanding how the key processes are relevant to an individual’s experience of living with cancer will help tailor future research and interventions (McCanney et al., 2018). Despite many trials of ACT interventions in cancer being conducted, RCTs did not complete mediation or process analysis meaning the strongest evidence for the effect of processes on distress could not be synthesised.

### 4.3. Future directions

Future research should look to conduct gold standard RCTs including mediation analysis to establish whether ACT-based treatment produces change in the corresponding processes such as reducing inflexibility or increasing flexible skills, which reduce distress. In addition, mediation analysis in longitudinal observational studies would provide further insight into the processes as mechanisms in relationships of independent variables and the outcome of distress. A key area of unclear bias in the included observational studies was the failure to report justification of sample size which is important to inform recruitment and determine the power and interpretation of data. Researchers publishing studies in line with guidelines such as STROBE (Cuschieri, 2019) would allow for more transparent reporting and a clearer assessment of risk of bias. Measures also need to be developed to capture the ACT process as defined. Further research on determining clearer definitions would aid stronger measurement development and consistency when responding to questionnaires. This would allow the model and potential mechanisms of change to be adequately tested to support intervention development and provide evidence of these processes translating into change (Arch et al., 2022). Additionally, work needs to ensure definitions are adequately translated and understood across different cultures, particularly in measurement development, as differences have been found (e.g., Trindade et al., 2021).

## 5. Conclusions

Overall, the present review is the first to consolidate the literature on ACT processes and distress in cancer and provides evidence from 110 manuscripts that have applied components of the ACT model to cancer. Most of the processes included in the meta-analyses had moderate to large associations with distress, supporting the use of the ACT model to understand distress in cancer. Across cancer diagnoses, the strongest associations were found for use of the inflexible processes of experiential avoidance and cognitive fusion and increased distress, whilst the use of more flexible processes, namely present moment awareness and self-compassion, were associated with lower distress. Our search failed to identify any RCTs which explored ACT processes as mediators of change in ACT interventions for distress in cancer and should be the focus for future studies. Further research needs to be conducted to identify relationships between distress and self-as-context, committed action, values and overall psychological flexibility. There was a paucity of longitudinal research conducted across all processes, which would allow the predictive ability of ACT processes on distress in cancer to be examined. Measures of processes also need to be developed based on clear conceptual definitions. Research developments in this area will help address and understand the maintenance and alleviation of the common experience of distress in those with cancer.

## Acknowledgements

The views expressed are those of the author and not necessarily those of the NHS, the NIHR or the Department of Health and Social Care.

## Supplementary Material

Supplemental Material
