# Supplementary material for: Acceptance and commitment therapy processes and their association with distress in cancer: a systematic review and meta-analysis
Source: Health Psychol Rev. 2023 Sep 25;18(3):456–77. doi: 10.1080/17437199.2023.2261518 (PMC11332408; doi:10.1080/17437199.2023.2261518)
Supplement: Supplemental Material [file RHPR_A_2261518_SM8095.pdf]

Supplementary materials contents:

Page 1: Table S1. *Measures eligible in the review*

Pages 2-3: Table S2. *Search strategy for all databases*

Page 4: S3. R code

Pages 5-33: Table S4. *Study characteristics of included studies and reference list*

Page 34-39: Table S5. *Data included in meta-analyses*

Pages 40-59: Table S6. *Data included in narrative synthesis*

Pages 60-61: S7. Narrative synthesis of additional data for meta-analysed processes

Pages 62-65: Table S8. *Risk of bias assessment*

Pages 66-69: Table S9. *GRADE assessment*

Table S1. *Measures eligible in the review*

Flexible processes<br>*Inflexible process (in italics)*	Measures eligible in the review
Acceptance<br>*Experiential avoidance*	Due to multiple measures for both, both are included and discussed separately<br>Including any process-based acceptance measure (emotions, self, pain, not resignation or acceptance as an end point)
Defusion<br>*Cognitive fusion*	Measures of cognitive fusion
Present moment awareness<br>*Loss of contact with the present*	Measures for mindfulness (total scores)
Self-as-context<br>*Self-as-content*	Measures of self-as-context
Values<br>*Lack of values clarity*	Measures of personal values/valued living
Committed action<br>*Inaction*	Measures of committed action<br>In addition, measures combining committed action and values were included
Self-compassion<br>*Self-judgement*	Self-compassion measures include variations of Neff (2003) measure (total scores of self-compassion or self-judgment)
Psychological flexibility<br>*Psychological inflexibility*	Multidimensional measures of the overarching process of psychological flexibility, incorporating elements of the 6 core processes.

Table S2. *Search strategy*

OVID	1. exp Neoplasms/, 2. cancer*.mp., 3. exp Metastasis/, 4. exp Oncology/, 5. oncology.mp., 6. tumo?r*.mp., 7. exp Distress/, 8. distress*.mp., 9. exp Psychological Stress/, 10. (psycho* adj stress*).mp., 11. (psycho* adj distress*).mp., 12. exp Anxiety/, 13. anxiet*.mp., 14. (anx* adj2 symptom*).mp., 15. exp "Depression (Emotion)"/, 16. depression.mp., 17. (depress* adj2 symptom*).mp. , 18. exp Emotional States/, 19. mood.mp., 20. (acceptance and commitment therapy).mp., 21. exp "Acceptance and Commitment Therapy"/, 22. acceptance.mp., 23. accept* adj5 thought*, 24. accept* adj5 feeling*, 25. accept* adj5 experience*, 26. accept* adj5 emotion*, 27. accept* adj5 cancer*, 28. accept* adj5 diagnos*, 29. accept* adj5 symptom*, 30. experiential acceptance, 31. psychological acceptance, 32. exp Experiential avoidance, 33. experiential avoidance.mp, 34. (psycho* adj flexibility).mp., 35. (psycho* adj inflexibility).mp., 36. exp Self-Compassion/, 37. self-compassion.mp., 38. self-kindness.mp., 39. (self adj compassion*).mp. , 40. (self adj kindness).mp., 41. exp Mindfulness/, 42. mindful*.mp., 43. being adj2 present, 44. present adj2 awareness, 45. present adj2 moment , 46. commit* adj2 action, 47. (cognitive adj fusion).mp. , 48. defusion.mp., 49. self-as-context.mp., 50. self adj2 context.mp., 51. contextualis* adj2 self, 52. self adj2 observer, 53. exp Personal values/, 54. personal adj value*.mp., 55. value* adj2 living, 56. value* adj2 action , 57. 1 or 2 or 3 or 4 or 5 or 6 , 58. 7 or 8 or 9 or 10 or 11 or 12 or 13 or 14 or 15 or 16 or 17 or 18 or 19, 59. 20 or 21 or 22 or 23 or 24 or 25 or 26 or 27 or 28 or 29 or 30 or 31 or 32 or 33 or 34 or 35 or 36 or 37 or 38 or 39 or 40 or 41 or 42 or 43 or 44 or 45 or 46 or 47 or 48 or 49 or 50 or 51 or 52 or 53 or 54 or 55 or 56, 60. 57 and 58 and 59.
CINAHL	1.(MH "Neoplasms+"), 2."cancer*" , 3.(MH "Oncology+"), 4."oncology" , 5.(MH "Neoplasm Metastasis+") , 6."tumor" , 7."tumour" , 8. (MH "Psycho-Oncology"), 9. "distress*", 10. (MH "Stress, Psychological+"), 11. "psych* distress*", 12. “psych* stress*”, 13. (MH "Anxiety+"), 14. "anxiet*", 15. "anxi* N2 symptom*", 16. "depression", 17. (MH "Depression+"), 18. "depress* N2 symptom*", 19. (MH "Affect"), 20. "mood", , 21. (MH "Acceptance and Commitment Therapy"), 22. "acceptance", 23. "accept* N5 thought*", 24. accept* N5 feeling*, 25. accept* N5 experience*, 26. accept* N5 emotion*, 27. accept* N5 cancer, 28. accept* N5 diagnos*, 29. accept* N5 symptom*, 30. experiential acceptance, 31. psychological acceptance, 32. experiential avoidance, 33. "psycho* flexibility", 34. "psycho* inflexibility", 35. "self-compassion", 36. "self kindness", 37. self N2 compassion*, 38. self N2 kindness, 39. (MH "Mindfulness"), 40. "mindful*", 41. being N2 present, 42. present N2 awareness, 43. present N2 moment, 44. “commit* N2 action” , 45. "cognitive fusion", 46. "defusion", 47. self-as-context, 48. self N2 context, 49. self N2 observer, 50. personal N2 value*.mp., 51. value* N2 living, 52. value* N2 action, 53. S1 OR S2 OR S3 OR S4 OR S5 OR S6 OR S7 OR S8, 54. S9 OR S10 OR S11 OR S12 OR S13 OR S14 OR S15 OR S16 OR S17 OR S18 OR S19 OR S20, 55. S21 OR S23 OR S24 OR S25 OR S26 OR S27 OR S28 OR S29 OR S30 OR 31 OR 32 OR 34, 56. S53 AND S54 AND S55
WoS	(TS=(neoplasm* OR cancer* OR metasta* OR oncology OR tumo$r*)<br>AND TS=(distress* OR "psych* near\2 stress" OR "psycho* near\2 distress*" OR anxious OR anxiet* OR "anx* near\2 symptoms" OR depress* OR "depress* near\2 symptoms" OR affect OR mood)<br>AND TS=("acceptance and commitment therapy" OR "acceptance" OR "accept* near\5 thought*" OR "accept* near\5 feeling*" OR "accept* near\5 experience" OR "accept* near\5 emotion*" OR<br>"accept* near\5 cancer*" OR "accept* near\5 diagnos*" OR<br>"experiential acceptance" OR "psychological acceptance" OR "experiential avoidance” OR

	"psycho* near\1 flexibility" OR "psycho* near\1 inflexibility" OR self-compassion OR self-kindness OR "self near\1 compass*" OR "self near\1 kindness" OR mindful* OR "being near\2 present" OR "present near\2 awareness" OR "present near\2 moment" OR "commit* near\2 action" OR "cognitive near\1 fusion" OR defusion OR self-as-context OR "self near\2 context" OR "contextualis* near\2 self" OR "self near\2 observer" OR "personal values" OR "value* near\2 living" OR "value* near\2 action"))
Cochrane	1. [Neoplasms] explode all trees, 2. [Psycho-oncology] explode all trees, 3. (cancer):ti,ab,kw, 4. (oncology):ti,ab,kw, 5. (tumo?r):ti,ab,kw, 6. [Neoplasm Metastasis] explode all trees, 7. #1 OR #2 OR #3 OR #4 OR #5 OR #6, 8. [Psychological Distress] explode , 9. [Stress, Psychological] explode all trees, 10. (distress*):ti,ab,kw, 11. psycho* next distress*, 12. psycho* next stress*, 13. [Anxiety] explode, 14. (anxiet*):ti,ab,kw, 15. anx* near/2 symptom*, 16. [Depression] explode, 17.(depression):ti,ab,kw, 18. depress* near/2 symptom*, 19. Affect] explode, 20. (mood):ti,ab,kw, 21. #8 OR #9 OR #10 OR #11 OR #12 OR #13 OR #14 OR #15 or #16 or #17 or #18 or #19 or #20, 22. Acceptance and Commitment Therapy] explode, 23. (acceptance):ti,ab,kw, 24. (accept* near/5 thought*):ti,ab,kw, 25. (accept* near/5 feeling*):ti,ab,kw, 26. (accept* near/5 experience*):ti,ab,kw, 27. (accept* near/5 emotion*):ti,ab,kw, 28. (accept* near/5 cancer*):ti,ab,kw, 29. (accept* near/5 diagnos*):ti,ab,kw, 30. (experiential next acceptance):ti,ab,kw, 31. (experiential next avoidance):ti,ab,kw, 32. (psychological next acceptance):ti,ab,kw, 33. (psychological next flexibility):ti,ab,kw, 34. psychological next inflexibility , 35. (self-compassion):ti,ab,kw, 36. (self-kindness):ti,ab,kw, 37. (self near/1 compass*):ti,ab,kw, 38. (self near/1 kindness):ti,ab,kw, 39. Mindfulness explode, 40. (mindful*):ti,ab,kw, 41. being next present , 42. present near/2 awareness, 43. present near/2 moment , 44. (commit* near/2 action):ti,ab,kw, 45. (cognitive next fusion):ti,ab,kw, 46. (defusion):ti,ab,kw, 47. (self-as-context):ti,ab,kw, 48. self near/2 context , 49. contexualis* near/2 self , 50. self near/2 observer, 51. personal next values, 52. value* near/2 living , 53. value* near/2 action , 54. #22 OR #23 OR #24 OR #25 OR #26 OR #27 OR #28 OR #29 OR #30 OR #31 OR #32 OR #33 OR #34 OR #35 OR #36 OR #37 OR #38 OR #39 OR #40 OR #41 OR #42 OR #43 OR #44 OR #45 OR #46 OR #47 OR #48 OR #49 OR #50 OR #51 OR #52 OR #53, 55. #7 AND #21 AND #54

S3. R code

```
Dataset1.cor <- metacor(cor,
                 n,
                 data = Dataset1,
                 studlab = Dataset1$Author,
                 sm = "ZCOR",
                 comb.fixed = FALSE,
                 comb.random = TRUE,
                 method.tau = "REML"
                 title = "Dataset1")
Dataset1.cor



m.gen.reg <- metareg(Dataset1.cor, ~moderator)
m.gen.reg
```

Table S4. *Study characteristics of included studies and reference list*

Study ID	Authors and country	*n*	Prop. females (%)	Mean age in years (SD)	Ethnicity/race	Sample	Detailed cancer sample diagnosis (%)	Mean time since diagnosis (SD)	Study design (and f/u period)
1	Aarstad, Lode, Larsen, Bru, and Aarstad (2011) Norway	96	22%	61 (11)	Not reported	Head & Neck	Lip (1%), oral cavity (29%), salivary glands (2%), pharynx (16%), larynx (42%), sinus (3%), unknown primary site (3%) Mixed stages	4 (2) years	CS
2	Afrashteh and Masoumi (2021) Iran	210	100%	38.97 (12.32)	Not reported	Breast	Breast cancer (100%)	Not reported	CS
3	Aguado Loi et al. (2013) USA	68	100%	55.4 (10.4)	Latinas: Colombian (30.9%), Puerto Rican (30.9%), Dominican (10.3%), Cuban (10.3%), Mexican (7.4%), Other (10.3%)	Breast	Breast (100%) Stages I-III	2.8 years (1.5)	CS
4	Al-Ghabeesh, Al-Kalaldah, Rayan, Al-Rifai, and Al-Halaiqa (2019) Jordan	234	100%	46.33 (11.51)	Jordanian (100%)	Breast	Breast (100%) Mixed stages	2.86 years (2.80)	CS
5	Aldaz, Hegarty, Conner, Perez, and Treharne (2019) New Zealand	31	61.30%	60.00 (14)	New Zealand, European/Pakeha (80.6%), Māori (6.5%), other European (12.9%)	Mixed	Breast (35.5%), rectum (19.4%), colon (9.7%), ovarian (9.7%), lung (6.5%), oesophageal (6.5%), prostate (6.5%), head and neck (3.2%), pancreas (3.2%) Mixed stages	Not reported	L (micro/7 days)
6	Arambasic, Sherman, and Elder (2019) Australia	92	100%	58.46 (8.77)	Not reported. Place of birth: Australia (80.5%), Other (19.5%)	Breast	Breast (100%) Mixed stages	82.14 months (19.34)	CS
7	Asuzu and Elumelu (2013) Nigeria	237	84.1%	49.91 (13.48)	Yoruba (32.9%), Igbo (44.4%), Hausas (6%), other (5.5%)	Mixed	Prostate (5.5%), breast (46.4%), cervical (25.7%), other (22.4%)	Not reported	CS
8	Babu (2020) USA	164	45.5%	44.21 (9.81)	Not reported	Mixed	Mixed Mixed stages 0-IV	Not reported	CS
9	Banner (2009) USA	69	100%	59.10 (13.46)	Not reported	Breast	Breast (100%) Mixed stages	7.77 months (3.11)	CS
10	Baziliansky and Cohen (2021) Israel	153	47.1%	64.5 (12.1)	Not reported	Colorectal	Colon (73.9%), rectal (26.1%) Mixed stages	13.7 months (4.1)	L (T1 baseline

Study ID	Authors and country	*n*	Prop. females (%)	Mean age in years (SD)	Ethnicity/race	Sample	Detailed cancer sample diagnosis (%)	Mean time since diagnosis (SD)	Study design (and f/u period)
									, T2 6 months)
11	Berrocal Montiel, Rivas Moya, Venditti, and Bernini (2016) Italy	64	100%	48.14 (9.36)	Not reported	Breast	Breast (100%) Mixed stages		L (T1 baseline , T2 6 months)
12	Black et al. (2016) USA	409 (358)	47.2%	Not reported	Hispanic (100%)	Colorect al	Colorectal (100%)	3.1 years (1.7)	CS
13	Brabbins (2016) UK	72	56.9%	Not reported	Not reported	Mixed	Breast (28%), prostate (26%), bowel (13%), lung (10%), other (23%) Mixed stages	Not reported	L (T1 baseline , T2 3 months)
14	K. W. Brown and Ryan (2003) USA	41	78%	55.31 (10.02)	Not reported	Mixed	Breast (78%), prostate (22%)	2.05 years (2.24)	CS (before RCT)
15	S. L. Brown, Roush, Marshall, Jones, and Key (2020) USA	61	44.3%	60.15 (11.71)	White/Caucasian (83.6%), American Indian or Alaska Native (1.6%), Asian or Asian American (3.3%), Black or African American (3.3%), Other (6.6%)	Mixed	Oral cavity and pharynx (6.6%), digestive system (26.2%), respiratory system (18.0%), skin (9.8%), genital system (9.8%), urinary system (3.3%), lymphoma (3.3%), myeloma (6.6%), brain & other nervous system (1.6%), leukaemia (3.3%), bones & joints (1.6%), other (6.6%)	Not reported	CS
16	Brunault et al. (2016) France	120	100%	56.4 (10.8)	Not reported	Breast	Breast (100%) Early stage	Not reported	CS
17	Cameron (2000) USA	44	57%	66 Males 69.1 (10.6); females 63 (12.3)	Male: White (89.5%) No response (10.5%) Female: White (60%), African American (20%), Asian American (4%), no response (16%)	Colorect al	Colorectal (100%) Mixed stages	21.6 months Males 22.4 (10.2) Females 21.2 (9.0)	L (baselin e, follow up 3 months)
18	Carlson and Brown (2005) Canada/USA	122	67.21%	49.55 (12.81)	Not reported	Mixed	Breast (51.6%), prostate (17.2%)	Not reported	CS
19	Carver et al. (1993) USA	59	100%	58.02 (10.83)	White (88%), Black (7%), Hispanic (5%)	Breast	Breast (100%) Early stages	Not reported	L (T0 1 day pre-surgery,

Study ID	Authors and country	*n*	Prop. females (%)	Mean age in years (SD)	Ethnicity/race	Sample	Detailed cancer sample diagnosis (%)	Mean time since diagnosis (SD)	Study design (and f/u period)
									T1 10 days post-surgery, T2 3 months, T3 6 months, T4 12 months)
20	Chen et al. (2021) Taiwan	90	54.4%	56.2 (10.5)	Not reported	Colorectal	Colon (58.9%), rectal (33.3%), colorectal (5.6%), anal (2.2%) Mixed stages	Not reported	CS
21	Cho, Kim, Durrani, Liao, and Milbury (2021) USA	78	50.65%	65 (10.44)	Non-Hispanic White (76%)	Lung	Non-small cell lung (100%) Stage IV	Not reported	CS
22	Ciarrochi, Fisher, and Lane (2011) Australia	107	50%	62 (median)	Australian born (49%), not stated (34%), born in England (8.4%)	Mixed	Breast (29%), prostate (14%), haematological (12%), lung (8%), colorectal (7%), not stated (7%) , skin (6.5%), pancreas (4%), head & neck (3%) ƚ	6 months	CS
23	Corman et al. (2021) France *same sample as Corman et al. (2022)	187	41.9%	52.07 (13.22)	Not reported	Blood	Acute leukaemia (35.7%), myelodysplastic syndrome (17%), myeloproliferative neoplasia (10.4%), non-Hodgkin lymphoma (12.1%), others e.g., Hodgkin lymphoma, chronic leukaemia (25.1%)	Not reported	CS
24	Corman et al. (2022) France *same sample as Corman et al. (2021)	187	41.9%	52.07 (13.22)	Not reported	Blood	Acute leukaemia (35.7%), myelodysplastic syndrome (17%), myeloproliferative neoplasia (10.4%), non-Hodgkin lymphoma (12.1%), others e.g., Hodgkin lymphoma, chronic leukaemia (25.1%)	Not reported	L (T0 before transplant, T1 5 months)
25	Costanzo, Lutgendorf, Rothrock, and Anderson (2006)	64	100%	62 (12.5)	Not reported	Gynae	On chemo: ovarian (72%), endometrium (14%), cervix (10%), fallopian (3%)	Not reported	CS

Study ID	Authors and country	*n*	Prop. females (%)	Mean age in years (SD)	Ethnicity/race	Sample	Detailed cancer sample diagnosis (%)	Mean time since diagnosis (SD)	Study design (and f/u period)
	USA						No chemo: endometrial (69%), cervical (28%), ovarian (3%)		
26	Deimling et al. (2006) * USA	321	59.2%	72.18 (7.7)	Black/African American (37.7%), White/Caucasian (62.3%)	Mixed	Breast (41.4%), prostate (28.7%), colorectal (29.9%)	10.4 years (5.5)	L (analysed data from initial interview)
27	Elsheshtawy, Abo-Elez, Ashour, Farouk, and El Zaafarany (2014) Egypt	56	100%	52 (13.3)	Not reported	Breast	Breast (100%) Early stages	Not reported	CS
28	Elumelu, Asuzu, and Akin-Odanye (2015) Nigeria	110	96.4%	47.04 (10.51)	Not reported	Breast	Breast (100%)	Not reported	CS
29	Fox (2002) USA	75	67%	55.45 (15.48)	Caucasian (85.3%), Asian American (5.3%), African American (3%), others (5.3%), not identified (1%)	Mixed	Breast (33.3%), gynaecological (13.3%), urogenital (13.3%), gastrointestinal (10.6%), skin and connective tissues (9.3%), head & neck (6.6%), lymphoma (4%), haematological (2.6%), brain (2.6%), lung (1.3%), unknown (2.6%)	30.58 weeks (35.83)	CS
30	Garcia et al. (2021) Brazil	183	53.55%	62.8 (12.7)	Not reported	Mixed	Breast (23.5%), prostate (16.39%), colorectal (18.03%), others (42.08%)	1 year 10 months (2.559)	CS
31	Garland et al. (2017) USA	97	55.6%	55.8 (14.3)	White (91.7%), Latino (3.1%), Black (2.1%), other (3.1%)	Mixed	Breast (26%), prostate (9%), colon (7%), lymphoma (7%), lung cancer (7%), melanoma (6%), ovarian (3%), others including neurological cancer, haematological cancer (35%) Mixed stages	Not reported	CS
32	Gillanders, Sinclair, MacLean, and Jardine (2015) UK	105	45%	Mean not reported	White British (93%), missing (7%)	Mixed	Urological (37%), breast (24%), haematological (23%), lung (6%), bowel (6%), gynae (5%), throat/neck (1%)	3.59 years (4.607)	CS
33	Glover (2015) UK	204	69.5%	57.18 (11.41)	British/English/Scottish (71.3%), White/Caucasian (21%), European (2.6%),	Mixed	Breast (24.5%), genitourinary (23.5%), gynaecological (18.0%), digestive	37.67 months (50.26)	CS

Study ID	Authors and country	*n*	Prop. females (%)	Mean age in years (SD)	Ethnicity/race	Sample	Detailed cancer sample diagnosis (%)	Mean time since diagnosis (SD)	Study design (and f/u period)
					American (1.0%), Asian (1.0%), Others/not disclosed (3.1%)		(12.5%), other (11.5%), head, neck & brain (10%)<br>*Those who felt personally responsible for their cancer n=155*		
34	González-Fernández et al. (2017)<br>Spain	122	100%	52.40 (7.26)	Not reported	Breast	Breast (100%)	1 month to 8 years	CS
35	Grozdziej (2015)<br>UK	77	100%	54 (9.7)	White (92.7%), Asian (1.8%), Black (1.8%), Mixed background (1.8%), Other (1.8%)	Breast	Breast (100%)	Not reported	CS
36	Hagan et al. (2017)<br>USA<br>[#] same sample as Nipp et al. (2016)	350	46%	64.86 (10.86)	White (92.3%)<br>African American (2.9%)<br>Asian (2.3%)<br>American Indian or Alaskan Native (1.1%)<br>Hispanic or Latino (2.6%)<br>Other (1.4%)	Mixed	Non-small cell lung cancer (44%), pancreatic (24.9%), small cell lung (8.6%), oesophageal (6.9%), other (15.7%)<br>Advanced stage	25 days (14.1)	CS
37	Ho, Fong, and Wan (2022)<br>China	127	58.3%	63.8 (8.9)	Not reported	Colorectal	Colorectal 100%<br>Mixed stages	Not reported	L (baseline T1, 2 months later T2, 8 months T3 but only cortisol at T3
38	Hsieh et al. (2021)<br>Taiwan	116	58.62%	54.85 (7.29)	Not reported	Lung	Non-small cell lung cancer (100%)<br>Mixed stages IIIB or IV	32.91 months (29.28)	CS
39	Hulbert-Williams and Storey (2016)<br>UK	129	55%	61.43 (16.8)	Not reported	Mixed	Breast (40.3%), colorectal (20.2%), prostate (19.4%), lung (18.6%), missing (1.5%)	6.8 months (3.1)	CS

Study ID	Authors and country	*n*	Prop. females (%)	Mean age in years (SD)	Ethnicity/race	Sample	Detailed cancer sample diagnosis (%)	Mean time since diagnosis (SD)	Study design (and f/u period)
40	Ikeuchi et al. (2020)<br>Japan	249	100%	59.5 years (12.44)	Not reported	Breast	Breast (100%)<br>Mixed stages I-III	Not reported	CS
41	Keeling, Bambrough, and Simpson (2013)<br>UK	74	53%	38.30 (10.67)	Not reported	Brain	Astrocytoma (58.2%), oligodendroglioma (31.3%), mixed glioma (4.5%), ependymoma (3%), other (3%)<br>Grades 1-2	27.69 months (19.79)	CS
42	Kelliher-Rabon, Sirois, Barton, and Hirsch (2022)<br>USA	235	64.3%	61.28 (27.63)	White (91.9%), Black/African American (1.7%), Hispanic or Latino/a (2.1%), American Indian or Alaskan native (0.90%), Asian (0.4%), other race (0.4%), multiracial (1.3%) and those declined (1.3%)	Mixed	Not reported	Not reported	CS
43	Kersting (2012)<br>USA	74 T1, 43 T2	75.7%	52.8 (12)	Caucasian (59.5%), African American (31.1%), Asian Pacific Islander (1.4%), Bi-racial (1.4%)	Mixed	Breast (45.9%), lung (9.5%), colon/rectal (6.8%)	6.2 months (4.7)	L
44	Kuba et al. (2019)<br>Germany	922	43%	64 (13.4)	Not reported	Blood	Haematological (100%)	8.9 years (4.5)	CS
45	Kuhlman et al. (2017)<br>USA	271	100%	56.23 (11.49)	Non-Hispanic white (71.6%), Asian (11.1%), Black (4.4%), Hispanic/Latina (3.7%), other (9.2%)	Breast	Breast (100%)	Not reported	CS
46	Lam, Lim, Kua, Griva, and Mahendran (2018)<br>Singapore	212	68%	49.26 (9.30)	Chinese (60%), Non-Chinese - Malay, Indian, Eurasian (40%)	Mixed	Breast (35%), others (65%) (nasopharyngeal, gynaecological, pancreatic, haematological, lung, gastrointestinal, brain, renal)	Not reported	CS
47	Lampic, Thurfjell, Bergh, Carlsson, and Sjödén (2002)<br>Sweden	32	100%	58 (9.6)	Not reported	Breast	Breast (100%)	Not reported	L (3 months, 1 year)
48	Larson et al. (2019)<br>USA	111	45.9%	58 (median)	Caucasian (97.3%), African American (0.9%), Asian American (0.9%), declined to respond (.9%)	Blood	Leukaemia (34.2%), lymphoma (30.6%), multiple myeloma (28.8%), other hematologic disease (6.3%)	Not reported	L (pre transplant and 1, 3, 6 months after)

Study ID	Authors and country	*n*	Prop. females (%)	Mean age in years (SD)	Ethnicity/race	Sample	Detailed cancer sample diagnosis (%)	Mean time since diagnosis (SD)	Study design (and f/u period)
49	Lei et al. (2021)<br>China	441	28.6%	60<br>(median)	Not reported	Lung	Lung cancer (100%)<br>Mixed stages I-IV	Not reported	CS
50	Lennon, Hevey, and Kinsella (2018) *<br>Ireland	92	0%	68.16<br>(9.66)	Irish (90%), Northern Irish (5%), British (3%), Sierra Leone (1%) ⱡ	Prostate	Prostate (100%)	40.63 months (38.43)	CS
51	Levkovich (2021)<br>Israel	170	100%	51.22<br>(12.15)	Jewish (81.1%), Arab (18.9%)	Breast	Breast cancer (100%)	Not reported	CS
52	Lewson et al. (2021)<br>USA	203	52.22%	63.16<br>(10.25)	Non-Hispanic white (75.86%), Black/African American (13.30%), Hispanic or Latino/a (3.94%), other (6.90%)	Mixed	Breast (25.12%), prostate (24.63%), gastrointestinal (25.12%), lung (25.12%)<br>Mixed stages I-II	3.50 years (2.98)	CS
53	Liu, Li, Zhang, Zhao, and Xu (2021)<br>China	230	100%	47.8 (9.1)	Not reported	Breast	Breast cancer (100%)<br>Mixed stages 0-III	Not reported	CS
54	Liu, Wang, Zhang, Wang, and Xu (2018)<br>China	202	100%	47.62<br>(8.90)	Not reported	Breast	Breast cancer (100%)<br>Mixed stages 0-III	Not reported	CS
55	Z. Liu et al. (2021)<br>China	290	30.3%	Not reported	Not reported	Liver	Hepatocellular carcinoma (100%)	Not reported	CS
56	Low, Stanton, Thompson, Kwan, and Ganz (2006)<br>USA	417	100%	58.1	White (87%)	Breast	Breast (100%)<br>Nonmetastatic	Not reported	CS (Baseline RCT)
57	Lv, Zhu, Wu, Yue, and Cui (2021)<br>China	82	43.9%	37.9<br>(10.3)	Not reported	Thyroid	Not reported	25.4 months (8.6)	CS
58	Mackay, Burdayron, and Korner (2021)<br>Canada	174	48.9%	59.2<br>(13.5)	Not reported	Melanoma	Melanoma (100%)<br>Mixed stages 0-IV	26.7 months (47.8)	CS at T3
59	Manne et al. (2018)<br>USA	174	100%	55.32<br>(10.28)	Caucasian (75.9%), non-Caucasian (23.6%), 1 missing	Gynae	Ovarian (65.5%)<br>Endometrial	3.90 months (1.90)	CS
60	McAteer and Gillanders (2019)<br>UK	286	0%	67 (7.81)	Not reported	Prostate	Prostate (100%)<br>Mixed stages	4.9 years (4.73)	CS

Study ID	Authors and country	*n*	Prop. females (%)	Mean age in years (SD)	Ethnicity/race	Sample	Detailed cancer sample diagnosis (%)	Mean time since diagnosis (SD)	Study design (and f/u period)
61	Millmann (2019)<br>USA	14	100%	62.79	Caucasian/White (93%), Native/Indian American (7%)	Gynae	Ovarian (86%), fallopian (14%<br>Stage III-IV	Not reported	CS
62	Mosher, Krueger, Secinti, and Johns (2021)<br>USA	201	49.25%	61.93 (11.93)	Non-Hispanic White (80.10%), Black (10.95%), Hispanic (2.99%), other (5.47%), missing (0.50%)	Mixed	Breast (24.88%), prostate (24.88%), lung (25.37%), gastrointestinal (24.88%)<br>Advanced stage	3.16 years (2.93)	CS
63	Mosher et al. (2017)<br>USA	80	100%	55.50 (11.26)	Non-Hispanic white (91.3%), other ethnicity – African American/Black, Hispanic and other (8.8%)	Breast	Breast (100%)<br>Stage IV	3.93 years (3.64)	CS
64	Nipp et al. (2016)<br>USA<br>[#] same sample as Hagan et al. (2017)	350	46%	64.86 (10.86)	White (92.3%)<br>African American (2.9%)<br>Asian (2.3%)<br>American Indian or Alaskan Native (1.1%)<br>Hispanic or Latino (2.6%)<br>Other (1.4%)	Mixed	Non-small cell lung cancer (44%), pancreatic (24.9%), small cell lung (8.6%), oesophageal (6.9%), other (15.7%)<br>Advanced stage	25 days (14.1)	CS
65	Novakov (2021)<br>Serbia	64	100%	58.36 (11.30)	Not reported	Breast	Not reported	Not reported	CS
66	Omid, Mohammadi, Jalaeikhoo, and Taghva (2017)<br>Iran	109	70.6%	Women 49.54 (8.7)<br>Men 50.37 (10.83)	Not reported	Mixed	Breast (36.7%), colon (25.7%), stomach (14.6%), lung (1.8%), liver (3.7%), leukaemia (8.3%), prostate (2.8%), vaginal (1.8%), ovarian (1.8%), testicular (0.9%), lymphoma (0.9%), bone marrow (0.9%)	2 years 5 months (2 years 4 months)	CS
67	Pinto-Gouveia, Duarte, Matos, and Fráguas (2014)<br>Portugal	63	82.5%	Male 55.45 (13.24)<br>Female 52.65 (10.01)	Not reported	Mixed	Breast (73.0%), lung (6.3%), prostate (4.8%), cervix (1.6%), stomach (3.2%), intestine (1.6%), others (9.5%)	Not reported	CS
68	Ploumen (2017)<br>Netherlands	108	69%		Not reported	Mixed	Breast (38%), other (62%)	Not reported	CS
69	Politi, Enright, and Weihs (2007)<br>USA	91 (79 com plet	100%	51.5 (10.3)	Caucasian (51.9%), African American (40.5%), Hispanic (3.8%), Asian American (2.5%), other (1.3%)	Breast	Breast (100%)<br>Early stages	13.9 months (4.9)	CS

Study ID	Authors and country	n	Prop. females (%)	Mean age in years (SD)	Ethnicity/race	Sample	Detailed cancer sample diagnosis (%)	Mean time since diagnosis (SD)	Study design (and f/u period)
		e data ?							
70	Poulin et al. (2016) Canada	76	76.3%	56.53 (9.37)	Caucasian (86.8%), Asian (2.6%), African (3.9%), other (3.9%)	Mixed	Breast and gastrointestinal (% not reported)	Not reported	CS
71	Priscilla et al. (2011) Malaysia	105	52%	40	Malay (60%), non-Malay (40%)	Blood	Haematological: non-Hodgkin lymphoma (24%), acute myelogenous leukaemia (23%), acute lymphoblastic leukaemia (14%), Hodgkin lymphoma (11%) and other haematological cancers (29%)	Not reported	CS
72	Przezdziecki (2017) Australia	206	100%	56.43 (9.73)	Country of birth: Australia (79.9%), New Zealand (3.9%), UK (11.3%), Europe (0.5%), Asia and Pacific (1%), America (1.5%)	Breast	Breast (100%)	67.18 months (63.96)	CS (before RCT)
73	Przezdziecki and Sherman (2016) Australia	152	100%	54.55 (9.79)	Country of birth: Australia (80.1%), UK/Europe (13.3%), other (6.6%)	Breast	Breast (100%)	Not reported	CS
74	Przezdziecki et al. (2013) Australia	279	100%	53.4 (9.40)	Country of birth Aus/NZ (81%), Britain/Ireland (11%), Asia (1%), Europe (1%), America (4%), Africa (2%)	Breast	Breast (100%)	Not reported	CS
75	Randell (2017) UK	75	92%	51.9 (12.0)	Not reported	Mixed	Breast (69.3%), haematological (8.0%), colorectal (5.3%), brain (4.0%), upper GI (2.7%), other (10.7%) includes lung, melanoma, gynaecological, thyroid, kidney and testicular	3.5 years (4.5)	CS
76	Raque-Bogdan, Lent, and Lamphere (2019) USA	275	100%	47 (11.12)	White, non-Latino (87%), African American (4%), American Indian (1%), Asian (3%), Multiracial (1%), Latino (4%)	Breast	Breast (100%)	4 years (4.22)	CS
77	Romano (2014) USA	76	92%	57 (10.22)	Caucasian (89.5%), Asian (6.6%), African American/Black (1.3%), Mixed (2%)	Mixed	Breast (66%), gynaecological (10.5%), blood, lymphatic, or bone marrow (7%), oral, head or neck (5%), colorectal (3%),	58.61 months (67.47)	CS

Study ID	Authors and country	*n*	Prop. females (%)	Mean age in years (SD)	Ethnicity/race	Sample	Detailed cancer sample diagnosis (%)	Mean time since diagnosis (SD)	Study design (and f/u period)
							kidney (3%), lung (1%), pancreatic (1%), unidentified (3%)		
78	Ross (2009)<br>USA	105	62%	56 (14.70)	Latinos: Mexican (70.5%), Central American (12.4%), South American (6.7%), Cuban (2.9%), Puerto Rican (4.8%), other (2.9%)	Mixed	Breast (36.2%), prostate (16.2%), colon/rectal (10.5%)<br>Mixed stages	Not reported	CS
79	Roussi, Krikeli, Hatzidimitriou, and Koutri (2007)<br>Greece	72	100%	54.13	Native Greeks (100%)	Breast	Breast (100%)	Not reported	L (pre-surgery, 2-3 days after surgery and 3 months later)
80	Salber (2016)<br>USA	233	100%	59.50 (10.68)	Race: White (89.7%), Black (9.0%), Other (1.3%)<br>Ethnicity: Hispanic (1.4%), non-Hispanic (98.6%)	Breast	Breast (100%)<br>Mixed stages	5.01 years (6.41)	CS
81	Saniah and Zainal (2010)<br>Malaysia	141	100%	50	Not reported	Breast	Breast (100%)	Not reported	CS
82	Schellekens et al. (2017)<br>Netherlands	88	33%	62.8 (8.2)	Not reported	Lung	Lung (100%)<br>Mixed stages	4.5 months (7.6)	CS
83	Seltzer (2021)<br>USA	82	0%	70.4	Black (69.5%), White (29.3%), Hispanic (12%)	Prostate	Not reported	52.8 months (57.5)	CS
84	Sevier-Guy, Ferreira, Somerville, and Gillanders (2021)<br>UK	144	0%	68.5 (7.2)	Not reported	Prostate	Not reported	6.0 years (4.13)	CS
85	Shapiro, McCue, Heyman, Dey, and Haller (2010)<br>USA	283	78%	54.6 (11.7)	White (79%), African American (19%), Hispanic (2%), Asian (1%)	Mixed	Breast (42%), lung (8%), colon (7%), lymphoma (6%), prostate (5%), leukaemia (5%), ovarian (4%), myeloma (4%), pancreatic (2%), other (17%)	Not reported	CS
86	Sherman, Woon, French, and Elder (2016)	75	100%	47.81 (8.86)	Country of birth: Australia (64.90%), New Zealand	Breast	Breast (100%)	Not reported	CS

Study ID	Authors and country	*n*	Prop. females (%)	Mean age in years (SD)	Ethnicity/race	Sample	Detailed cancer sample diagnosis (%)	Mean time since diagnosis (SD)	Study design (and f/u period)
	Australia				(4.10%), UK/Ireland (17.60%), Asia (6.66%), Europe (4.05%), Africa (2.70%)				
87	Siwik, Phillips, Zimmaro, Salmon, and Sephton (2021) USA	58	67.2%	Not reported	Caucasian (63.8%), Black/African American (17.2%), Hispanic or Latino (1.7%), Asian/Asian American (1.7%), other (1.7%), missing (11.9%)	Lung	Non-small cell lung cancer (100%) Mixed stages	Not reported	CS
88	Stanton, Danoff-Burg, and Huggins (2002) USA	70	100%	52.63 (11.94)	White (91%), African American (7%), Latina (1%)	Breast	Breast (100%) Early stages	Not reported	L
89	Stanton, Wiley, Krull, Crespi, and Weihs (2018) USA	460	100%	56.4 (12.6)	Non-Latina White (67.6%), Latina (19.3%)	Breast	Breast (100%) Early stages	2.1 months (0.81)	L
90	Swash, Bramwell, and Hulbert-Williams (2017) UK	91	53.2%	61 (12.4) extracted from diss	White (97.50%), Black Caribbean (1.30%), Chinese (1.30%)	Blood	Haematological	Not reported	CS
91	Tamagawa et al. (2013) Canada	272 (227)	100%	54.58 (10.32)	Not reported	Breast	Breast (100%) Mixed stages I-III	24.08 months (27.38)	CS
92	Taylor-Ford (2014) USA	102	52%	55.07 (11.24)	Non-Hispanic, White (69%), Black (3%), Hispanic (13%), Asian (12%), other (3%)	Colorect al	Colorectal (100%) Advanced stage	821 days (941)	CS
93	Thune-Boyle, Stygall, Keshtgar, Davidson, and Newman (2013) UK	155	100%	55.7 (13.5)	Caucasian (82.6%), Black (4.5%), Asian (3.9%), Oriental (1.9%), other (7.2%)	Breast	Breast (100%) Early stages	Not reported	CS
94	Todorov, Sherman, and Kilby (2019) Australia	195	100%	Mean not reported	Country of birth: Australia (68.8%), Other (67%)	Breast	Breast (100%) Mixed stages	Not reported	CS
95	Trevino et al. (2012) USA	53	66%	33.89 (5.70)	White (92.5%), African American (1.9%), Asian American (1.9%), Hispanic (3.8%)	Mixed	Breast (39.6%), brain tumour (13.2%), leukaemia/lymphoma (9.4%), colon (5.7%), soft tissue (3.8%) Advanced stages	3.72 years (3.05)	CS

Study ID	Authors and country	*n*	Prop. females (%)	Mean age in years (SD)	Ethnicity/race	Sample	Detailed cancer sample diagnosis (%)	Mean time since diagnosis (SD)	Study design (and f/u period)
96	Trindade, Marta-Simões, Ferreira, and Pinto-Gouveia (2018a)<br>Portugal	75	100%	57.60 (10.18)	Not reported	Breast	Breast (100%)<br>Mixed stages	Not reported	CS
97	Trindade, Marta-Simões, Ferreira, and Pinto-Gouveia (2018b)<br>Portugal	82	100%	50.21 (9.76)	Not reported	Breast	Breast (100%)	Not reported	CS
98	Trindade et al. (2020)<br>Portugal	40	100%	60 (10.13)	Not reported	Breast	Breast (100%)<br>Stages I-III	Not reported	L (T1 baseline, T2 6 months)
99	van der Donk et al. (2020)<br>Netherlands	245	24.9%	65.35 (12.01)	Not reported	Mixed	Urological (52.8%), lung (8.5%), colorectal (6.4%), haematological (6.8%), gynaecological (8.5%), bone & soft tissue (3.0%), other (2.1%), mixed (11.9%)	2.39 years (1.39)	CS
100	van Laarhoven et al. (2011)<br>Netherlands	151	53%	58 (13)	Not reported	Mixed	Breast (29%), prostate (12%), testis (7%), lung (10%), colon/rectum (11%), melanoma (7%), other (24%)	Not reported	CS
101	Vick (2018)<br>USA	75	100%	51.45 (12.34)	White, non-Hispanic (84%), African American (15%), Asian American (1%)	Breast	Breast (100%)<br>Mixed stages	6.7 months (median)	CS
102	Vickberg (2000)<br>USA	169	100%	59 (11.41)	African American (8%), White (74%), Hispanic (10%), Multi-ethnic or other (8%)	Breast	Breast (100%)<br>Mixed stages	3 years (1.42)	CS
103	Walsh et al. (2018)<br>North America and Ireland	241	0%	64.02 (7.76)	North American (46.90%), European (47.7%)	Prostate	Prostate (100%)<br>Mixed stages	Not reported	CS
104	W. Xu, Zhou, Fu, and Rodriguez (2017)<br>China	176	46%	Not reported	Not reported	Gastro	Gastrointestinal (100%, stomach, colon, oesophageal)<br>Mixed stages	Not reported	CS
105	X. Xu, Ou, Xie, Cheng, and Chen (2019)<br>China	156	46.8%	52.3	Not reported	Mixed	Cancer patients with chronic pain	Not reported	CS
106	Zamanian et al. (2021)<br>Iran	221	Not reported	47.14 (9.13)	Not reported	Breast	Not reported	18.31 weeks (15.05)	CS

Study ID	Authors and country	*n*	Prop. females (%)	Mean age in years (SD)	Ethnicity/race	Sample	Detailed cancer sample diagnosis (%)	Mean time since diagnosis (SD)	Study design (and f/u period)
107	Zarei, Musarezaie, and Ashouri (2021)<br>Iran	190	42.10%	Not reported	Not reported	Gastro	Gastrointestinal (100%)	Not reported	CS
108	Zhong, Goh, Li, Bao, and Xu (2020)<br>China	292	65%	52.2 (11.8)	Not reported	Gastro	Gastrointestinal: gastric, intestinal, oesophagus (100%)	Not reported	CS
109	Zhu et al. (2020)<br>China	301	60.40%	50.07 (13.09)	Chinese	Mixed	Breast (22.30%), lung (16.70%), gastric (10.50%), gynaecological (16.70%), colorectal (4.50%), pancreas (2.80%), liver (2.10%), lymphoma (4.9%), multiple malignant tumours (3.10%), other (16.40%)<br>Mixed stages	14.25 months (16.44)	CS
110	Zhu et al. (2019)<br>China	243	65.8%	50.78 (11.61)	Not reported	Mixed	Breast (28.4%), lung (15.1%), gastric (3.3%), gynaecological (22.4%), colorectal (5.9%), lymph (3.9%), others (14.5%), missing (6.6%)	Not reported	L (T1 diagnosis, T2 start and T3 end of treatment)

*Note: * author contacted; # = same sample indicated; n = sample size; SD = standard deviation; f/u: follow up; CS = cross sectional study; L = longitudinal study; T1, 2 etc: time 1, time 2…); RCT: randomised controlled trial*

Reference list of included studies

Aarstad, A. K., Lode, K., Larsen, J. P., Bru, E., & Aarstad, H. J. (2011). Choice of psychological coping in laryngectomized, head and neck squamous cell carcinoma patients versus multiple sclerosis patients. *European Archives of Oto-Rhino-Laryngology, 268*(6), 907-915. doi:https://dx.doi.org/10.1007/s00405-010-1417-6

Afrashteh, M. Y., & Masoumi, S. (2021). Psychological well-being and death anxiety among breast cancer survivors during the Covid-19 pandemic: the mediating role of self-compassion. *BMC Women's Health, 21*(1). doi:10.1186/s12905-021-01533-9

Aguado Loi, C. X., Baldwin, J. A., McDermott, R. J., McMillan, S., Martinez Tyson, D., Yampolskaya, S., & Vandeweerd, C. (2013). Risk factors associated with increased depressive symptoms among Latinas diagnosed with breast cancer within 5 years of survivorship. *Psycho-oncology, 22*(12), 2779-2788. doi:https://dx.doi.org/10.1002/pon.3357

Al-Ghabeesh, S. H., Al-Kalaldah, M., Rayan, A., Al-Rifai, A., & Al-Halaiqa, F. (2019). Psychological distress and quality of life among Jordanian women diagnosed with breast cancer: The role of trait mindfulness. *European journal of cancer care, 28*(5), e13082. doi:https://dx.doi.org/10.1111/ecc.13082

Aldaz, B. E., Hegarty, R. S., Conner, T. S., Perez, D., & Treharne, G. J. (2019). Is avoidance of illness uncertainty associated with distress during oncology treatment? A daily diary study. *Psychology & Health, 34*(4), 422-437. doi:http://dx.doi.org/10.1080/08870446.2018.1532511

Arambasic, J., Sherman, K. A., & Elder, E. (2019). Attachment styles, self-compassion, and psychological adjustment in long-term breast cancer survivors. *Psycho-oncology, 28*(5), 1134-1141. doi:http://dx.doi.org/10.1002/pon.5068

Asuzu, C. C., & Elumelu, T. N. (2013). Assessing cancer patients' quality of life and coping mechanisms in Radiotherapy Department of the University College Hospital, Ibadan. *Psycho-oncology, 22*(10), 2306-2312. doi:10.1002/pon.3290

Babu, P. (2020). *Relations between depression, anxiety, and health behaviors : the role of psychological flexibility.* University of Toledo, USA, Retrieved from https://www.proquest.com/dissertations-theses/relations-between-depression-anxiety-health/docview/2515773522/se-2?accountid=11862

Banner, A. T. (2009). *The effects of spirituality on anxiety and depression among breast cancer patients : the moderating effects of alexithymia and mindfulness.* (unpubished doctoral thesis). University of North Carolina at Greensboro, USA, Retrieved from http://libres.uncg.edu/ir/listing.aspx?id=2467 WorldCatDissertations database.

Baziliansky, S., & Cohen, M. (2021). Post-treatment psychological distress among colorectal cancer survivors: Relation to emotion regulation patterns and personal resources. *International journal of behavioral medicine, 28*, 591-601. doi:https://dx.doi.org/10.1007/s12529-020-09952-y

Berrocal Montiel, C., Rivas Moya, T., Venditti, F., & Bernini, O. (2016). On the contribution of psychological flexibility to predict adjustment to breast cancer. *Psicothema, 28*(3), 266-271. doi:https://dx.doi.org/10.7334/psicothema2015.271

Black, D. S., Li, M. J., Ihenacho, U., Nguyen, N. T., de Fatima Reyes, M., Milam, J., . . . Figueiredo, J. C. (2016). Shared health characteristics in Hispanic colorectal cancer patients and their primary social support person following primary diagnosis. *Psycho-oncology, 25*(9), 1028-1035. doi:https://dx.doi.org/10.1002/pon.3938

Brabbins, L. J. (2016). *Accepting the 'Big C': Exploring the acceptance - quality of Life relationship in a cancer population.* (unpublished Clinical Psychology doctoral thesis). University of Lincoln, UK,

Brown, K. W., & Ryan, R. M. (2003). The benefits of being present: mindfulness and its role in psychological well-being. *Journal of Personality & Social Psychology, 84*(4), 822-848. doi: 10.1037/0022-3514.84.4.822

Brown, S. L., Roush, J. F., Marshall, A. J., Jones, C., & Key, C. (2020). The Intervening Roles of Psychological Inflexibility and Functional Impairment in the Relation between Cancer-related Pain and Psychological Distress. *International journal of behavioral medicine, 27*(1), 100-107. doi:https://dx.doi.org/10.1007/s12529-019-09838-8

Brunault, P., Champagne, A. L., Huguet, G., Suzanne, I., Senon, J. L., Body, G., . . . Camus, V. (2016). Major depressive disorder, personality disorders, and coping strategies are independent risk factors for lower quality of life in non-metastatic breast cancer patients. *Psycho-oncology, 25*(5), 513-520. doi:https://dx.doi.org/10.1002/pon.3947

Cameron, C. L. (2000). Coping and adjustment to colorectal cancer. *Dissertation Abstracts International: Section B: The Sciences and Engineering, 60*(8-B), 4206.

Carlson, L. E., & Brown, K. W. (2005). Validation of the Mindful Attention Awareness Scale in a cancer population. *Journal of psychosomatic research, 58*(1), 29-33. doi:10.1016/j.jpsychores.2004.04.366

Carver, C. S., Pozo, C., Harris, S. D., Noriega, V., Scheier, M. F., Robinson, D. S., . . . Clark, K. C. (1993). How coping mediates the effect of optimism on distress: A study of women with early stage breast cancer. *Journal of Personality and Social Psychology, 65*(2), 375-390. doi:http://dx.doi.org/10.1037/0022-3514.65.2.375

Chen, L. T., Cheng, C. T., Huang, I. P., Chang, J. H., Chang, N. T., & Hsiao, F. H. (2021). The multiple mediating effects of cancer threat appraisal and quality of life on the association between mindfulness and depression for colorectal cancer survivors. *Psycho-oncology, 30*(6), 853-862. doi:https://dx.doi.org/10.1002/pon.5644

Cho, D., Kim, S., Durrani, S., Liao, Z., & Milbury, K. (2021). Associations Between Spirituality, Mindfulness, and Psychological Symptoms Among Advanced Lung Cancer Patients and Their

Spousal Caregivers. *Journal of pain and symptom management, 61*(5), 898-908. doi:http://dx.doi.org/10.1016/j.jpainsymman.2020.10.001

Ciarrochi, J., Fisher, D., & Lane, L. (2011). The link between value motives, value success, and well-being among people diagnosed with cancer. *Psycho-oncology, 20*(11), 1184-1192. doi:http://dx.doi.org/10.1002/pon.1832

Corman, M., Rubio, M.-T., Cabrespine, A., Brindel, I., Bay, J.-O., Peffault De La Tour, R., & Dambrun, M. (2021). Mental health and quality of life of patients undergoing hematopoietic stem cell transplantation (HSCT) prior to hospitalization: A cross-sectional complete state health study. *Health Psychology and Behavioral Medicine, 9*(1), 70-83. doi:https://dx.doi.org/10.1080/21642850.2021.1873140

Corman, M., Rubio, M. T., Cabrespine, A., Brindel, I., Bay, J. O., De la Tour, R. P., & Dambrun, M. (2022). Mindful Non-judging and Experiential Avoidance Before Allogeneic Hematopoietic Stem Cell Transplantation (HSCT) are Associated to Post-traumatic Stress Disorder (PTSD) Symptomatology 5 Months Later. *Mindfulness, 13*(1), 238-247. doi:10.1007/s12671-021-01788-1

Costanzo, E. S., Lutgendorf, S. K., Rothrock, N. E., & Anderson, B. (2006). Coping and quality of life among women extensively treated for gynecologic cancer. *Psycho-oncology, 15*(2), 132-142. doi:10.1002/pon.930

Deimling, G. T., Wagner, L. J., Bowman, K. F., Sterns, S., Kercher, K., & Kahana, B. (2006). Coping among older-adult, long-term cancer survivors. *Psycho-oncology, 15*(2), 143-159. doi:http://dx.doi.org/10.1002/pon.931

Elsheshtawy, E. A., Abo-Elez, W. F., Ashour, H. S., Farouk, O., & El Zaafarany, M. I. (2014). Coping strategies in egyptian ladies with breast cancer. *Breast Cancer, 8*, 97-102. doi:https://dx.doi.org/10.4137/BCBCR.S14755

Elumelu, T. N., Asuzu, C. C., & Akin-Odanye, E. O. (2015). Impact of active coping, religion and acceptance on quality of life of patients with breast cancer in the department of

radiotherapy, UCH, Ibadan. *BMJ supportive & palliative care, 5*(2), 175-180. doi:10.1136/bmjspcare-2012-000409

Fox, R. B. (2002). Religious identity, religious practices, and spiritual coping in adults undergoing treatment for cancer. *Dissertation Abstracts International: Section B: The Sciences and Engineering, 62*(10-B), 4783.

Garcia, A. C. M., Camargos Junior, J. B., Sarto, K. K., Silva Marcelo, C. A. D., Paiva, E. M. D. C., Nogueira, D. A., & Mills, J. (2021). Quality of life, self-compassion and mindfulness in cancer patients undergoing chemotherapy: A cross-sectional study. *European Journal of Oncology Nursing, 51*, 101924. doi:http://dx.doi.org/10.1016/j.ejon.2021.101924

Garland, E. L., Thielking, P., Thomas, E. A., Coombs, M., White, S., Lombardi, J., & Beck, A. (2017). Linking dispositional mindfulness and positive psychological processes in cancer survivorship: A multivariate path analytic test of the mindfulness-to-meaning theory. *Psycho-oncology, 26*(5), 686-692. doi:http://dx.doi.org/10.1002/pon.4065

Gillanders, D. T., Sinclair, A. K., MacLean, M., & Jardine, K. (2015). Illness cognitions, cognitive fusion, avoidance and self-compassion as predictors of distress and quality of life in a heterogeneous sample of adults, after cancer. *Journal of Contextual Behavioral Science, 4*(4), 300-311. doi:http://dx.doi.org/10.1016/j.jcbs.2015.07.003

Glover, L. (2015). *The Relationship Between Self-Blame, Self-Compassion and Psychological WellBeing for Individuals Living with Chronic Physical Health Conditions.* (unpublished Clinical Psychology doctoral thesis). The Universtiy of Hull, UK.,

González-Fernández, S., Fernández-Rodríguez, C., Mota-Alonso, M., García-Teijido, P., Pedrosa, I., & Pérez-Álvarez, M. (2017). Emotional state and psychological flexibility in breast cancer survivors. *European Journal of Oncology Nursing, 30*, 75-83. doi:10.1016/j.ejon.2017.08.006

Grozdziej, A. (2015). *The influence of treatment type and psychological factors on fear of recurrence, distress and health behaviours amongst breast cancer survivors* (unpublished Clinical Psychology doctoral thesis). University of Bath, UK.,

Hagan, T. L., Fishbein, J. N., Nipp, R. D., Jacobs, J. M., Traeger, L., Irwin, K. E., . . . et al. (2017). Coping in Patients With Incurable Lung and Gastrointestinal Cancers: a Validation Study of the Brief COPE. *Journal of pain and symptom management, 53*(1), 131-138. doi:10.1016/j.jpainsymman.2016.06.005

Ho, R. T., Fong, T. C., & Wan, A. H. (2022). Effects of self-compassion on diurnal cortisol pattern via positive affect in colorectal cancer survivors. *Mindfulness, 13*(1), 211-221. doi:https://dx.doi.org/10.1007/s12671-021-01786-3

Hsieh, C. C., Ho, C. C., Lin, Z. Z., Yu, C. J., Jow, G. M., Huang, F. Y., . . . Hsiao, F. H. (2021). Trait mindfulness and depressive symptoms in non-small cell lung cancer patients: the mediating roles of quality of life and meaning in life. *Psychology & Health, 36*(9), 1102-1114. doi:http://dx.doi.org/10.1080/08870446.2020.1825713

Hulbert-Williams, N. J., & Storey, L. (2016). Psychological flexibility correlates with patient-reported outcomes independent of clinical or sociodemographic characteristics. *Supportive care in cancer, 24*(6), 2513-2521. doi:https://dx.doi.org/10.1007/s00520-015-3050-9

Ikeuchi, K., Ishiguro, H., Nakamura, Y., Izawa, T., Shinkura, N., & Nin, K. (2020). The relation between mindfulness and the fatigue of women with breast cancer: Path analysis. *BioPsychoSocial Medicine, 14*. doi:http://dx.doi.org/10.1186/s13030-020-0175-y

Keeling, M., Bambrough, J., & Simpson, J. (2013). Depression, anxiety and positive affect in people diagnosed with low-grade tumours: The role of illness perceptions. *Psycho-oncology, 22*(6), 1421-1427. doi:http://dx.doi.org/10.1002/pon.3158

Kelliher-Rabon, J., Sirois, F. M., Barton, A. L., & Hirsch, J. K. (2022). Self-compassion and suicidal behavior: Indirect effects of depression, anxiety, and hopelessness across increasingly vulnerable samples. *Self and Identity, 21*(2), 223-243. doi:10.1080/15298868.2021.1884592

Kersting, K. E. (2012). *Dispositional mindfulness in people diagnosed with cancer : the relationship to depressive symptoms and well-being.* (MSc thesis). Virginia Commonwealth University, USA, Retrieved from http://scholarscompass.vcu.edu/etd/2665 WorldCatDissertations database.

Kuba, K., Weißflog, G., Götze, H., García-Torres, F., Mehnert, A., & Esser, P. (2019). The relationship between acceptance, fatigue, and subjective cognitive impairment in hematologic cancer survivors. *International Journal of Clinical and Health Psychology, 19*(2), 97-106. doi:http://dx.doi.org/10.1016/j.ijchp.2018.12.001

Kuhlman, K. R., Boyle, C. C., Irwin, M. R., Ganz, P. A., Crespi, C. M., Asher, A., . . . Bower, J. E. (2017). Childhood maltreatment, psychological resources, and depressive symptoms in women with breast cancer. *Child Abuse & Neglect, 72*, 360-369. doi:http://dx.doi.org/10.1016/j.chiabu.2017.08.025

Lam, K. F., Lim, H. A., Kua, E. H., Griva, K., & Mahendran, R. (2018). Mindfulness and cancer patients' emotional states: A latent profile analysis among newly diagnosed cancer patients. *Mindfulness, 9*(2), 521-533. doi:http://dx.doi.org/10.1007/s12671-017-0794-y

Lampic, C., Thurfjell, E., Bergh, J., Carlsson, M., & Sjödén, P.-O. (2002). "Life values before versus after a breast cancer diagnosis": Erratum. *Research in nursing & health, 25*(3), 242. doi:http://dx.doi.org/10.1002/nur.10038

Larson, A. G., Morris, K. J., Juckett, M. B., Coe, C. L., Broman, A. T., & Costanzo, E. S. (2019). Mindfulness, Experiential Avoidance, and Recovery From Hematopoietic Stem Cell Transplantation. *Annals of behavioral medicine, 53*(10), 886-895. doi:https://dx.doi.org/10.1093/abm/kay097

Lei, H., Tian, X., Jin, Y.-F., Tang, L., Chen, W.-Q., & Jimenez-Herrera, M. F. (2021). The chain mediating role of social support and stigma in the relationship between mindfulness and psychological distress among Chinese lung cancer patients. *Supportive care in cancer, 29*(11), 6761-6770. doi:https://dx.doi.org/10.1007/s00520-021-06278-7

Lennon, J., Hevey, D., & Kinsella, L. (2018). Gender role conflict, emotional approach coping, self-compassion, and distress in prostate cancer patients: A model of direct and moderating effects. *Psycho-oncology, 27*(8), 2009-2015. doi:http://dx.doi.org/10.1002/pon.4762

Levkovich, I. (2021). Coping strategies and their impact on emotional distress and fatigue among breast cancer survivors: A cross-sectional survey. *The Cancer Journal 27*(2), 83-89. doi:http://dx.doi.org/10.1097/PPO.0000000000000505

Lewson, A. B., Johns, S. A., Krueger, E., Chinh, K., Kidwell, K. M., & Mosher, C. E. (2021). Symptom experiences in post-treatment cancer survivors: associations with acceptance and commitment therapy constructs. *Supportive care in cancer, 29*(7), 3487-3495. doi:http://dx.doi.org/10.1007/s00520-020-05854-7

Liu, X., Li, J., Zhang, Q., Zhao, Y., & Xu, W. (2021). Being beneficial to self and caregiver: the role of dispositional mindfulness among breast cancer patients. *Supportive care in cancer, 29*(1), 239-246. doi:https://dx.doi.org/10.1007/s00520-020-05435-8

Liu, X., Wang, L., Zhang, Q., Wang, R., & Xu, W. (2018). Less Mindful, More Struggle and Growth: Mindfulness, Posttraumatic Stress Symptoms, and Posttraumatic Growth of Breast Cancer Survivors. *The Journal of Nervous and Mental Disease, 206*(8), 621-627. Retrieved from https://journals.lww.com/jonmd/Fulltext/2018/08000/Less_Mindful,_More_Struggle_and_Growth__.8.aspx

Liu, Z., Li, M., Jia, Y., Wang, S., Wang, C., & Chen, L. (2021). Relationship between Mindfulness and Psychological Distress in Patients with Hepatocellular Carcinoma: The Mediation Effect of Self-regulation. *American Journal of Health Behavior, 45*(6), 1041-1049. doi:https://dx.doi.org/10.5993/AJHB.45.6.8

Low, C. A., Stanton, A. L., Thompson, N., Kwan, L., & Ganz, P. A. (2006). Contextual life stress and coping strategies as predictors of adjustment to breast cancer survivorship. *Annals of behavioral medicine, 32*(3), 235-244. doi:10.1207/s15324796abm3203_10

Lv, J., Zhu, L., Wu, X., Yue, H., & Cui, X. (2021). Study on the correlation between postoperative mental flexibility, negative emotions, and quality of life in patients with thyroid cancer. *Gland surgery, 10*(8), 2471-2476. doi:https://dx.doi.org/10.21037/gs-21-424

Mackay, S., Burdayron, R., & Korner, A. (2021). Factor structure of the Brief COPE in patients with melanoma. *Canadian Journal of Behavioural Science / Revue canadienne des sciences du comportement, 53*(1), 78-83. doi:https://dx.doi.org/10.1037/cbs0000184

Manne, S. L., Kashy, D. A., Virtue, S., Criswell, K. R., Kissane, D. W., Ozga, M., . . . Rodriguez, L. (2018). Acceptance, social support, benefit-finding, and depression in women with gynecological cancer. *Quality of Life Research: An International Journal of Quality of Life Aspects of Treatment, Care & Rehabilitation, 27*(11), 2991-3002. doi:http://dx.doi.org/10.1007/s11136-018-1953-x

McAteer, G., & Gillanders, D. (2019). Investigating the role of psychological flexibility, masculine self-esteem and stoicism as predictors of psychological distress and quality of life in men living with prostate cancer. *European journal of cancer care, 28*(4), e13097. doi:https://dx.doi.org/10.1111/ecc.13097

Millmann, M. M. (2019). *Psychological distress, pain, physical impairment, and acceptance among women with ovarian cancer.* (M.S. Thesis). Missouri State University, USA, Retrieved from https://purl.missouristate.edu/library/etd/Millmann.Megan-2019-SP.pdf

Mosher, C. E., Krueger, E., Secinti, E., & Johns, S. A. (2021). Symptom experiences in advanced cancer: Relationships to acceptance and commitment therapy constructs. *Psycho-oncology, 30*, 1485-1491. doi:https://dx.doi.org/10.1002/pon.5712

Mosher, C. E., Tometich, D. B., Hirsh, A., Rand, K. L., Johns, S. A., Matthias, M. S., . . . Miller, K. (2017). Symptom experiences in metastatic breast cancer patients: relationships to activity engagement, value-based living, and psychological inflexibility. *Psycho-oncology, 26*(11), 1944-1951. doi:http://dx.doi.org/10.1002/pon.4283

Neff, K. D. (2003). The Development and Validation of a Scale to Measure Self-Compassion. *Self and Identity, 2*(3), 223-250. doi:10.1080/15298860309027

Nipp, R. D., El-Jawahri, A., Fishbein, J. N., Eusebio, J., Stagl, J. M., Gallagher, E. R., . . . et al. (2016). The relationship between coping strategies, quality of life, and mood in patients with incurable cancer. *Cancer, 122*(13), 2110-2116. doi:10.1002/cncr.30025

Novakov, I. (2021). Emotional state, fatigue, functional status and quality of life in breast cancer: Exploring the moderating role of psychological inflexibility. *Psychology, health & medicine, 26*(7), 877-886. doi:https://dx.doi.org/10.1080/13548506.2020.1842896

Omid, A., Mohammadi, A. S., Jalaeikhoo, H., & Taghva, A. (2017). Dispositional mindfulness, psychological distress, and posttraumatic growth in cancer patients. *Journal of Loss and Trauma, 22*(8), 681-688. doi:http://dx.doi.org/10.1080/15325024.2017.1384783

Pinto-Gouveia, J., Duarte, C., Matos, M., & Fráguas, S. (2014). The protective role of self-compassion in relation to psychopathology symptoms and quality of life in chronic and in cancer patients. *Clinical psychology & psychotherapy, 21*(4), 311-323. doi:http://dx.doi.org/10.1002/cpp.1838

Ploumen, I. M. G. (2017). *Dispositional mindfulness and its possible function as buffer for distress that is caused by cancer diagnosis and treatment.* (Thesis). Tilburg University, The Netherlands, Retrieved from http://arno.uvt.nl/show.cgi?fid=143208

Politi, M. C., Enright, T. M., & Weihs, K. L. (2007). The effects of age and emotional acceptance on distress among breast cancer patients. *Supportive care in cancer, 15*(1), 73-79. doi:10.1007/s00520-006-0098-6

Poulin, P., Romanow, H., Rahbari, N., Small, R., Smyth, C., Hatchard, T., . . . Wilson, K. G. (2016). The relationship between mindfulness, pain intensity, pain catastrophizing, depression, and quality of life among cancer survivors living with chronic neuropathic pain. *Supportive care in cancer, 24*(10), 4167-4175. doi:10.1007/s00520-016-3243-x

Priscilla, D., Hamidin, A., Azhar, M., Noorjan, K., Salmiah, M., & Bahariah, K. (2011). Coping styles in patients with haematological cancer in a Malaysian hospital. *East Asian Archives of Psychiatry, 21*(2), 44-51.

Przezdziecki, A. (2017). *My changed body: breast cancer, body image, distress and self-compassion.* (Ph. D. thesis). Macquarie University, Australia, WorldCatDissertations database.

Przezdziecki, A., & Sherman, K. A. (2016). Modifying affective and cognitive responses regarding body image difficulties in breast cancer survivors using a self-compassion-based writing intervention. *Mindfulness, 7*(5), 1142-1155. doi:http://dx.doi.org/10.1007/s12671-016-0557-1

Przezdziecki, A., Sherman, K. A., Baillie, A., Taylor, A., Foley, E., & Stalgis-Bilinski, K. (2013). My changed body: Breast cancer, body image, distress and self-compassion. *Psycho-oncology, 22*(8), 1872-1879. doi:http://dx.doi.org/10.1002/pon.3230

Randell, K. (2017). *Applying Acceptance-Based Therapies to Help People Live Well after Cancer Treatment.* (Doctorate in Clinical Psychology unpiblished clinical doctoral thesis). University of Edinburgh, UK,

Raque-Bogdan, T. L., Lent, R. W., & Lamphere, B. (2019). Test of a social cognitive model of well-being among breast cancer survivors. *Journal of health psychology, 24*(5), 661-670. doi:10.1177/1359105316681431

Romano, E. M. (2014). Exploring the mechanisms of mindfulness: Experiential avoidance and health outcomes among cancer survivors. *Dissertation Abstracts International: Section B: The Sciences and Engineering, 74*(12-B(E)), No Pagination Specified.

Ross, P. J. (2009). Dispositional optimism, fatalism, and quality of life in Latino cancer patients. *Dissertation Abstracts International: Section B: The Sciences and Engineering, 70*(3-B), 1978.

Roussi, P., Krikeli, V., Hatzidimitriou, C., & Koutri, I. (2007). Patterns of coping, flexibility in coping and psychological distress in women diagnosed with breast cancer. *Cognitive Therapy and Research, 31*(1), 97-109. doi:http://dx.doi.org/10.1007/s10608-006-9110-1

Salber, K. E. (2016). Experiential avoidance and distress in breast cancer patients. *Dissertation Abstracts International: Section B: The Sciences and Engineering, 76*(12-B(E)), No Pagination Specified.

Saniah, A., & Zainal, N. (2010). Anxiety, depression and coping strategies in breast cancer patients on chemotherapy. *Malaysian Journal of Psychiatry, 19*(2).

Schellekens, M. P., Karremans, J. C., van der Drift, M. A., Molema, J., van den Hurk, D. G., Prins, J. B., & Speckens, A. E. (2017). Are mindfulness and self-compassion related to psychological distress and communication in couples facing lung cancer? A dyadic approach. *Mindfulness, 8*(2), 325-336. doi:http://dx.doi.org/10.1007/s12671-016-0602-0

Seltzer, J. D. (2021). *Fear of cancer recurrence: The roles of psychological flexibility and social support as possible protective factors.* (82). Kean University, USA, Retrieved from https://www.proquest.com/dissertations-theses/fear-cancer-recurrence-roles-psychological/docview/2444867669/se-2?accountid=11862

Sevier-Guy, L. J., Ferreira, N., Somerville, C., & Gillanders, D. (2021). Psychological flexibility and fear of recurrence in prostate cancer. *European journal of cancer care, 30*(6), e13483. doi:https://dx.doi.org/10.1111/ecc.13483

Shapiro, J. P., McCue, K., Heyman, E. N., Dey, T., & Haller, H. S. (2010). Coping-related variables associated with individual differences in adjustment to cancer. *Journal of psychosocial oncology, 28*(1), 1-22. doi:https://dx.doi.org/10.1080/07347330903438883

Sherman, K., Woon, S., French, J., & Elder, E. (2016). Body image and psychological distress in nipple-sparing mastectomy: The roles of self-compassion and appearance investment. *Psycho-oncology, 26*(3), 337-345. doi:http://dx.doi.org/10.1002/pon.4138

Siwik, C. J., Phillips, K., Zimmaro, L., Salmon, P., & Sephton, S. E. (2021). Depressive symptoms among patients with lung cancer: Elucidating the roles of shame, guilt, and self-compassion. *Journal of health psychology*. doi:10.1177/1359105320988331

Stanton, A. L., Danoff-Burg, S., & Huggins, M. E. (2002). The first year after breast cancer diagnosis: Hope and coping strategies as predictors of adjustment. *Psycho-oncology, 11*(2), 93-102. doi:http://dx.doi.org/10.1002/pon.574

Stanton, A. L., Wiley, J. F., Krull, J. L., Crespi, C. M., & Weihs, K. L. (2018). Cancer-related coping processes as predictors of depressive symptoms, trajectories, and episodes. *Journal of consulting and clinical psychology, 86*(10), 820-830. doi:http://dx.doi.org/10.1037/ccp0000328

Swash, B., Bramwell, R., & Hulbert-Williams, N. J. (2017). Unmet psychosocial supportive care needs and psychological distress in haematological cancer survivors: The moderating role of psychological flexibility. *Journal of Contextual Behavioral Science, 6*(2), 187-194. doi:http://dx.doi.org/10.1016/j.jcbs.2017.02.005

Tamagawa, R., Giese-Davis, J., Speca, M., Doll, R., Stephen, J., & Carlson, L. E. (2013). Trait mindfulness, repression, suppression, and self-reported mood and stress symptoms among women with breast cancer. *Journal of Clinical Psychology, 69*(3), 264-277. doi:http://dx.doi.org/10.1002/jclp.21939

Taylor-Ford, M. (2014). *Mindfulness among patients with advanced colorectal cancer.* (unpublished Ph D Thesis). University of Southern California, USA, Retrieved from http://cdm15799.contentdm.oclc.org/cdm/ref/collection/p15799coll3/id/518027

Thune-Boyle, I. C., Stygall, J., Keshtgar, M. R., Davidson, T. I., & Newman, S. P. (2013). Religious/spiritual coping resources and their relationship with adjustment in patients newly diagnosed with breast cancer in the UK. *Psycho-oncology, 22*(3), 646-658. doi:http://dx.doi.org/10.1002/pon.3048

Todorov, N., Sherman, K. A., & Kilby, C. J. (2019). Self-compassion and hope in the context of body image disturbance and distress in breast cancer survivors. *Psycho-oncology, 28*(10), 2025-2032. doi:http://dx.doi.org/10.1002/pon.5187

Trevino, K. M., Maciejewski, P. K., Fasciano, K., Greer, J., Partridge, A., Kacel, E. L., . . . Prigerson, H. G. (2012). Coping and psychological distress in young adults with advanced cancer. *The Journal of Supportive Oncology, 10*(3), 124-130. doi:https://dx.doi.org/10.1016/j.suponc.2011.08.005

Trindade, I. A., Marta-Simões, J., Ferreira, C., & Pinto-Gouveia, J. (2018a). Chronic illness-related cognitive fusion explains the impact of body dissatisfaction and shame on depression symptoms in breast cancer patients. *Clinical psychology & psychotherapy*, No Pagination Specified. doi:http://dx.doi.org/10.1002/cpp.2323

Trindade, I. A., Marta-Simões, J., Ferreira, C., & Pinto-Gouveia, J. (2018b). Developments on committed action: Validity of the CAQ-8 and analysis of committed action's role in depressive symptomatology in breast cancer patients and healthy individuals. *Clinical psychology & psychotherapy, 25*(1), e42-e50. doi:http://dx.doi.org/10.1002/cpp.2125

Trindade, I. A., Marta-Simões, J., Mendes, A. L., Borrego, M., Ponte, A., Carvalho, C., . . . Pinto-Gouveia, J. (2020). A longitudinal preliminary analysis on the role of experiential avoidance in breast cancer patients' reported health outcomes. *Psycho Oncology, 08*, 812-814. doi:https://dx.doi.org/10.1002/pon.5330

van der Donk, L. J., Fleer, J., Tovote, A., Ranchor, A. V., Smink, A., Mul, V. E., . . . Schroevers, M. J. (2020). The role of mindfulness and self-compassion in depressive symptoms and affect: A comparison between cancer patients and healthy controls. *Mindfulness, 11*, 883-894. doi:http://dx.doi.org/10.1007/s12671-019-01298-1

van Laarhoven, H., Schilderman, J., Bleijenberg, G., Donders, R., Vissers, C. C., Verhagen, C. A., & Prins, J. (2011). Coping, quality of life, depression, and hopelessness in cancer patients in a curative and palliative, end-of-life care setting. *Cancer nursing, 34*(4), 302-314. doi:10.1097/NCC.0b013e3181f9a040

Vick, R. M. (2018). Meaning making and dispositional mindfulness among women with breast cancer. *Dissertation Abstracts International: Section B: The Sciences and Engineering, 79*(9-B(E)), No Pagination Specified.

Vickberg, S. M. J. (2000). The possibility of breast cancer recurrence: Coping with perceived threats. *Dissertation Abstracts International: Section B: The Sciences and Engineering, 61*(4-B), 2227.

Walsh, D. M., Morrison, T. G., Conway, R. J., Rogers, E., Sullivan, F. J., & Groarke, A. (2018). A model to predict psychological- and health-related adjustment in men with prostate cancer: The role of post traumatic growth, physical post traumatic growth, resilience and mindfulness. *Frontiers in Psychology 9*(ArtID 136). doi:http://dx.doi.org/10.3389/fpsyg.2018.00136

Xu, W., Zhou, Y., Fu, Z., & Rodriguez, M. (2017). Relationships between dispositional mindfulness, self-acceptance, perceived stress, and psychological symptoms in advanced gastrointestinal cancer patients. *Psycho-oncology, 26*(12), 2157-2161. doi:https://dx.doi.org/10.1002/pon.4437

Xu, X., Ou, M., Xie, C., Cheng, Q., & Chen, Y. (2019). Pain Acceptance and Its Associated Factors among Cancer Patients in Mainland China: A Cross-Sectional Study. *Pain Research & Management, 2019*, 7. doi:https://dx.doi.org/10.1155/2019/9458683

Zamanian, H., Amini-Tehrani, M., Jalali, Z., Daryaafzoon, M., Ala, S., Tabrizian, S., & Foroozanfar, S. (2021). Perceived social support, coping strategies, anxiety and depression among women with breast cancer: Evaluation of a mediation model. *European Journal of Oncology Nursing, 50*, 101892. doi:http://dx.doi.org/10.1016/j.ejon.2020.101892

Zarei, K., Musarezaie, A., & Ashouri, E. (2021). The Relationship between Self-Compassion and the Experience of Memorial Symptoms in Patients with Gastrointestinal Cancer. *Iranian Journal of Nursing and Midwifery Research, 26*(4), 289-294. doi:10.4103/ijnmr.IJNMR_284_20

Zhong, M., Goh, P. H., Li, D., Bao, J., & Xu, W. (2020). Dispositional mindfulness as a moderator between perceived stress and psychological symptoms in Chinese digestive tract cancer patients. *Journal of health psychology, 25*(6), 810-818. doi:https://dx.doi.org/10.1177/1359105317736576

Zhu, L., Wang, J., Liu, S., Xie, H., Hu, Y., Yao, J., . . . Fleer, J. (2020). Self-compassion and symptoms of depression and anxiety in Chinese cancer patients: The mediating role of illness perceptions. *Mindfulness, 11*(10), 2386-2396. doi:https://dx.doi.org/10.1007/s12671-020-01455-x

Zhu, L., Yao, J., Wang, J., Wu, L., Gao, Y., Xie, J., . . . Schroevers, M. J. (2019). The predictive role of self-compassion in cancer patients' symptoms of depression, anxiety, and fatigue: A longitudinal study. *Psycho-oncology, 28*(9), 1918-1925. doi:http://dx.doi.org/10.1002/pon.5174

Table S5. *Data included in meta-analyses*

Study ID	Author (year)	*n*	Sample	Advanced stage of cancer (%)	Process Measure	Outcome Measure	Correlations	Average effect size (correlation)
Experiential avoidance								
61	Millmann (2019)	14	Gynae	18	AAQII7	POMS	0.11	0.11
57	Lv et al. (2021)	82	Thyroid	NA	AAQII7	SAS anx	0.29*	0.30
						SDS dep	0.31**	
15	S. L. Brown et al. (2020)	61	Mixed	NA	PIP avoidance scale	DT	0.33*	0.33
8	Babu (2020)	164	Mixed	0.6	BEAQ15	DASS anx	0.47**	0.49
						DASS dep	0.51**	
65	Novakov (2021)	64	Breast	0	AAQII 8 items	DASS21 anx	0.49**	0.52
						DASS21 dep	0.57**	
						DASS21 stress	0.49**	
62	Mosher et al. (2021)	201	Mixed	100	AAQII7	PROMIS anx	0.56**	0.59
						PROMIS dep	0.62**	
23	Corman et al. (2021)	187	Blood	NA	AFQ	HADS anx	0.57***	0.48
						HADS dep	0.37***	
11	Berrocal Montiel et al. (2016)	64	Breast	39	AAQII7	HADS anx	0.61***	0.52
						HADS dep	0.55***	
						PANAS	0.38*	
83	Seltzer (2021)	82	Prostate	NA	AAQII7	HADS anx	0.66**	0.61
						HADS dep	0.55**	
52	Lewson et al. (2021)	203	Mixed	0	AAQII7	PROMIS anx	0.66**	0.67
						PROMIS dep	0.67**	
75	Randell (2017)	75	Mixed	0	AAQII7	HADS anx	0.68**	0.52
						HADS dep	0.45**	
					BEAQ15	HADS anx	0.48**	
						HADS dep	0.43**	
80	Salber (2016)	233	Breast	17.6	AAQII7	HADS anx	0.72**	0.65
						HADS dep	0.65**	
						DT	0.57**	
90	Swash et al. (2017)	74	Blood	0	AAQII7	HADS anx	0.73**	0.67
						HADS dep	0.60**	
63	Mosher et al. (2017)	80	Breast	100	AAQII7	PROMIS anx	0.75**	0.72
						PROMIS dep	0.68**	

34	González-Fernández et al. (2017)	122	Breast	0	AAQII7	HADS anx<br>HADS dep<br>BSI/GSI	0.77**<br>0.79**<br>0.70**	0.76
77	Romano (2014)	76	Mixed	14	AAQII10	HADS anx<br>HADS dep	0.77**<br>0.65**	0.72
98	Trindade et al. (2020)	40	Breast	0	AAQ7	DASS21 dep<br>DASS21 stress	0.71***<br>0.66***	0.69
Acceptance								
19	Carver et al. (1993)	59	Breast	0	COPE 3 items	POMS distress presurgery	-0.68**	-0.68
79	Roussi et al. (2007)	72	Breast	NA	bCOPE Greek	POMS distress presurgery	-0.43***	-0.43
29	Fox (2002)	75	Mixed	NA	COPE 3 items	POMS distress<br>IES	-0.42***<br>-0.24*	-0.33
41	Keeling et al. (2013)	74	Brain	0	bCOPE	HADS anx<br>HADS dep	-0.31(no *p* value)<br>-0.21(no *p* value)	-0.26
58	Mackay et al. (2021)	174	Skin	2.9	bCOPE	PHQ4 anx<br>PHQ4 dep	-0.29**<br>-0.36**	-0.33
36	Hagan et al. (2017)	350	Mixed	100	bCOPE	HADS anx<br>HADS dep	-0.29***<br>-0.37***	-0.33
59	Manne et al. (2018)	174	Gynae	21.3	COPE	BDI dep	-0.35**	-0.35
93	Thune-Boyle et al. (2013)	155	Breast	0	COPE	HADS anx<br>HADS dep	-0.25**<br>-0.25**	-0.25
56	Low et al. (2006)	417	Breast	0	COPE	CES dep<br>IES	-0.25*<br>-0.19*	-0.22
27	Elsheshtawy et al. (2014)	56	Breast	0	bCOPE	HADS anx<br>HADS dep	-0.19 ns<br>-0.26 ns	-0.22
106	Zamanian et al. (2021)	221	Breast	NA	bCOPE	DASS21 anx<br>DASS21 dep	-0.18**<br>-0.23**	-0.21
17	Cameron (2000)	25	Colorectal females	0	COPE	POMS distress	0.20 ns	0.20
17	Cameron (2000)	19	Colorectal males	21.2	COPE	POMS distress	0.25 ns	0.25
78	Ross (2009)	105	Mixed	36	bCOPE	CES dep	-0.15 ns	-0.15
1	Aarstad et al. (2011)	96	Head & neck	18	COPE	BDI dep	-0.14 ns	-0.14

26	Deimling et al. (2006)	321	Mixed	0	COPE 3 items	POMS anx	0.05 ns	0.06
						CES dep	0.07 ns	
Cognitive fusion								
57	Lv et al. (2021)	82	Thyroid	-	CFQ9 Chinese	SAS anx	0.30*	0.28
						SDS dep	0.25*	
15	S. L. Brown et al. (2020)	61	Mixed	-	PIPS subscale	DT	0.36**	0.36
62	Mosher et al. (2021)	201	Mixed	-	CFQ7	PROMIS anx	0.57**	0.57
						PROMIS dep	0.57**	
52	Lewson et al. (2021)	203	Mixed	-	CFQ7	PROMIS anx	0.64**	0.63
						PROMIS dep	0.62**	
75	Randell (2017)	75	Mixed	-	CFQ7	HADS anx	0.66**	0.54
						HADS dep	0.40**	
8	Babu (2020)	164	Mixed	-	CFQ7	DASS anx	0.67**	0.67
						DASS dep	0.66**	
32	Gillanders et al. (2015)	105	Mixed	-	CFQ7	HADS anx	0.72**	0.62
						HADS dep	0.50**	
96	Trindade et al. (2018a)	75	Breast	-	CFQ CI	DASS21 dep	0.69***	0.69
Present moment awareness								
55	Z. Liu et al. (2021)	290	Liver	0	FFMQ	HADS distress	-0.69**	-0.69
70	Poulin et al. (2016)	76	Mixed	0	FFMQ	PHQ9 dep	-0.64***	-0.64
77	Romano (2014)	76	Mixed	14	MAAS	HADS anx	-0.64**	-0.59
						HADS dep	-0.54**	
43	Kersting (2012)	74	Mixed	9.5	MAAS	CES dep	-0.54***	-0.54
38	Hsieh et al. (2021)	116	Lung	56	FFMQ	BDI dep	-0.54***	-0.54
75	Randell (2017)	75	Mixed	0	MAAS	HADS anx	-0.53**	-0.45
						HADS dep	-0.37**	
53	X. Liu et al. (2021)	230	Breast	0	MAAS	GAD7	-0.51**	-0.51
						PHQ9	-0.53**	
						PTSS	-0.50**	
20	Chen et al. (2021)	90	Colorectal	0	FFMQ	BDI dep	-0.49***	-0.49
99	van der Donk et al. (2020)	245	Mixed	0	FFMQ	CES dep	-0.47**	-0.44
						PANAS	-0.40**	
103	Walsh et al. (2018)	241	Prostate	0	Freiburg	HADS anx	-0.46**	-0.48
						HADS dep	-0.50**	
92	Taylor-Ford (2014)	102	Colorectal	100	MAAS	HADS anx	-0.45**	-0.43
						CES dep	-0.33**	

						EORTC	-0.49**	
23	Corman et al. (2021)	187	Blood	NA	FFMQ	HADS anx	-0.45***	-0.38
						HADS dep	-0.31***	
91	Tamagawa et al. (2013)	227	Breast	1.4	MAAS	POMS distress	-0.49**	-0.49
82	Schellekens et al. (2017)	88	Lung	24	FFMQ24	HADS distress	-0.49**	-0.49
14	K. W. Brown and Ryan (2003)	41	Mixed	0	MAAS	POMS distress	-0.43**	-0.43
45	Kuhlman et al. (2017)	271	Breast	0	MAAS	CES dep	-0.43**	-0.43
40	Ikeuchi et al. (2020)	249	Breast	0	MAAS	HADS anx	-0.41**	-0.42
						HADS dep	-0.43**	
21	Cho et al. (2021)	78	Lung	100	MAAS	CES dep	-0.40***	-0.39
						IES	-0.38**	
4	Al-Ghabeesh et al. (2019)	234	Breast	5.1	MAAS	HADS anx	-0.29**	-0.27
						HADS dep	-0.25**	
31	Garland et al. (2017)	97	Mixed	49	FFMQ	DASS21 distress	-0.40***	-0.40
66	Omid et al. (2017)	109	Mixed	NA	FFMQ	DASS42 distress	-0.39**	-0.39
104	W. Xu et al. (2017)	176	Gastro	41.5	MAAS	GHQ distress	-0.26**	-0.26
108	Zhong et al. (2020)	292	Gastro	29	MAAS	GHQ distress	-0.19***	-0.19
9	Banner (2009)	69	Breast	0	FFMQ	TAQ anx	0.42**	0.42
						CESD short dep	0.41**	
8	Babu (2020)	164	Mixed	0.6	FFMQ	DASS anx	0.60**	0.59
						DASS dep	0.57**	
12	Black et al. (2016)	409	Colorectal	0	MAAS	DASS stress	-0.42***	-0.42
49	Lei et al. (2021)	441	Lung	74.8	FFMQ total	DT	-0.15**	-0.15
54	Liu et al. (2018)	202	Breast	0	MAAS	PTSS	-0.47**	-0.47
101	Vick (2018)	75	Breast	41	FFMQ	DT	-0.47**	-0.47
18	Carlson and Brown (2005)	122	Mixed	11	MAAS	POMS	-0.39 (no *p* value)	-0.39
Self-compassion								
33	Glover (2015)	155	Mixed	NA	SCS 26	HADS anx	-0.61**	-0.57
						HADS dep	-0.52**	
76	Raque-Bogdan et al. (2019)	275	Breast	0	SCS 26	PANAS NA	-0.55**	-0.55
82	Schellekens et al. (2017)	88	Lung	24	SCS SF Dutch	HADS distress	-0.55**	-0.55
107	Zarei et al. (2021)	190	Gastro	NA	SCS 26	Psych MSAS distress	-0.54***	-0.54
86	Sherman et al. (2016)	75	Breast	NA	SCS SF	DASS21 anx	-0.53**	-0.54
						DASS21 dep	-0.60**	
						DASS21 stress	-0.47**	

2	Afrashteh and Masoumi (2021)	210	Breast	0	SCS 26	BAI anx	-0.53**	-0.51
						BDI dep	-0.48**	
42	Kelliher-Rabon et al. (2022)	235	Mixed	NA	SCS SF	MHP anx	-0.53**	-0.58
						MHP dep	-0.63**	
72	Przezdziecki (2017)	197	Breast	0	SCS 26	DASS21 anx	-0.52**	-0.58
						DASS21 dep	-0.58**	
						DASS21 stress	-0.63**	
						IES	-0.55**	
						PANAS NA	-0.60**	
35	Grozdziej (2015)	77	Breast	0	SCS SF	PHQ+GAD distress	-0.50***	-0.50
32	Gillanders et al. (2015)	105	Mixed	NA	SCS 26	HADS anx	-0.50**	-0.47
						HADS dep	-0.44**	
99	van der Donk et al. (2020)	245	Mixed	0	SCS 24 Dutch	CES dep	-0.47**	-0.42
						PANAS NA	-0.37**	
94	Todorov et al. (2019)	195	Breast	4.1	SCS SF	DASS21 anx	-0.43***	-0.56
						DASS21 dep	-0.56***	
						DASS21 stress	-0.66***	
73	Przezdziecki and Sherman (2016)	148	Breast	0	SCS 26	DASS21 anx	-0.41**	-0.52
						DASS21 dep	-0.61**	
						DASS21 stress	-0.53**	
110	Zhu et al. (2019)	243	Mixed	11.8	SCS SF T1	STAI6 T1 anx	-0.40**	-0.39
						PHQ9 T1 dep	-0.38**	
50	Lennon et al. (2018)	92	Prostate	NA	SCS 26	DASS21 distress	-0.40**	-0.40
6	Arambasic et al. (2019)	92	Breast	7.3	SCS 26	DASS21 stress	-0.39*	-0.39
74	Przezdziecki et al. (2013)	279	Breast	NA	SCS 26	DASS21 anx	-0.39**	-0.49
						DASS21 dep	-0.57**	
						DASS21 stress	-0.51**	
109	Zhu et al. (2020)	301	Mixed	31.6	SCS SF	STAI anx	-0.39**	-0.38
						PHQ dep	-0.37**	
10	Baziliansky and Cohen (2021)	153		0	SCS SF	BSI distress	-0.29***	-0.29
51	Levkovich (2021)	170	Breast	0	SCS SF	BSI anx	-0.20*	-0.19
						BSI dep	-0.17*	
						BSI distress (inc somatisation)	-0.20*	

NA: Not Available; AAQ: Acceptance and Action Questionnaire; HADS: Hospital Anxiety and Depression Scale; PROMIS: Patient Reported Outcomes Measurement Information System; DASS: Distress, Anxiety and Stress Scales; SAS: Self-rating Anxiety Scale; AFQ: Avoidance and Fusion Questionnaire; BEAQ: Brief Experiential Avoidance Questionnaire; SDS: Self-rating Depression

Scale; COPE: Coping Orientation to Problems Experienced Inventory; bCOPE: Brief COPE; POMS: Profile of Mood States; PHQ: Patient Health Questionnaire; BDI: Beck Depression Inventory; CES-D: Centre for Epidemiologic Studies Depression; CFQ: Cognitive Fusion Questionnaire; CFQ CI; Cognitive Fusion Questionnaire Chronic Illness; MAAS: Mindful Attention Awareness Scale; FFMQ: Five Facets of Mindfulness Scale; TAQ: Trimodal Anxiety Questionnaire; Freiburg: Freiburg Mindfulness Inventory; GAD: Generalised Anxiety Disorder Scale; GHQ: General Health Questionnaire; SCS: Self-compassion Scale (SF – Short Form); STAI: State Trait Anxiety Scale; BAI: Beck Anxiety Inventory; BSI: Brief Symptom Inventory; MHP: Multidimensional Health Profile; MSAS: Memorial Symptom Assessment Scale

Table S6. *Data included in narrative synthesis*

Study ID	Author (year)	*n*	Sample	Process Measure	Outcome Measure	Correlation	Other results i.e., regression
Experiential avoidance							
75	Randell (2017)	75	Mixed	AAQII7<br>BEAQ15	HADS distress<br>HADS	0.63**<br>0.50**	Experiential avoidance was not significantly associated with anxiety whilst controlling for fear of recurrence, valued living, mindfulness and cognitive fusion<br>β = 0.01 ns<br>Experiential avoidance was not significantly associated with depression whilst controlling for fear of recurrence, valued living mindfulness and cognitive fusion<br>β = 0.12 ns
34	González-Fernández et al. (2017)	122	Breast	AAQII7	HADS	-	Experiential avoidance was significantly associated with anxiety whilst controlling for insomnia<br>β = 0.71***<br>Experiential avoidance was significantly associated with depression whilst controlling for fatigue, social impairment and environmental reward<br>β = 0.33***
					BSI/GSI		Experiential avoidance was significantly associated with distress whilst controlling for fatigue and insomnia<br>β = 0.53***
11	Berrocal Montiel et al. (2016)	64	Breast	AAQII7	HADS	-	Experiential avoidance at T1 significantly predicted anxiety at T2 (6m), whilst controlling for T1 anxiety and months since diagnosis<br>β = 0.64***<br>Experiential avoidance at T1 significantly predicted depression at T2 (6m), whilst controlling for T1 depression and living with partner<br>β = 0.37*

					PANAS		Experiential avoidance significantly predicted an increase in negative affect at T2 (6m) whilst controlling for T1 negative affect<br>β = 0.46***
77	Romano (2014)	76	Mixed	AAQII10	HADS	-	Direct effect of experiential avoidance on anxiety in mediation analysis<br>β = 0.60***<br>Direct effect of experiential avoidance on depression in mediation analysis<br>β = 0.50***
48	Larson et al. (2019)	111	Blood	BEAQ15	IDAS somatic anxiety	-	Experiential avoidance did not significantly predict anxiety whilst controlling for transplant graft type, age, sex and time since diagnosis<br>β = 0.03 ns<br>Experiential avoidance was not significantly associated with depression whilst controlling for transplant graft type, age, sex and time since diagnosis<br>β = 0.11 ns
13	Brabbins (2016)	72	Mixed	AAQII7 greater acceptance	HADS anx	-0.80**	Reverse scored AAQ significantly predicted lower anxiety at T2, whilst controlling for illness perceptions, disengagement and self-blame<br>β = -0.57*
					HADS dep	-0.60**	Reverse scored AAQ did not significantly predict reduced depression at T2 whilst controlling for illness perceptions and distraction<br>β = -0.19 ns
					FACT-B EWB	0.73**	Reverse scored AAQ was not significantly associated with greater EWB at T2 controlling for illness perceptions<br>β = 0.15 ns
44	Kuba et al. (2019)	922	Blood	AAQII7 greater acceptance	GAD7<br>PHQ9	-0.68**<br>-0.65**	-
39	Hulbert-Williams and Storey (2016)	129	Mixed	AAQII7 greater acceptance	HADS	-0.60**	Reverse scored AAQ was significantly associated with lower anxiety whilst controlling for age, time since diagnosis and treatment intent<br>β = -0.31***

						-0.47**	Reverse scored AAQ was significantly associated with a reduction in depression whilst controlling for age, time since diagnosis and treatment intent<br>β = -0.27***
					PANAS	-0.64***	Reverse scored AAQ was associated with reduction in negative affect whilst controlling for age, time since diagnosis and treatment intent<br>β = -0.58***
23	Corman et al. (2021)	187	Blood	AFQ	HADS		Experiential avoidance was significantly associated with an increase in anxiety<br>β = 0.29***<br>Experiential avoidance was not significantly associated with increased depression<br>β = 0.12 ns<br>Both controlling for optimism and mindfulness
				AAQII10 greater acceptance		-0.58***	Reverse scored AAQ was significantly associated with lower anxiety<br>β = -0.21*
						-0.44***	Reverse scored AAQ was not significantly associated with lower depression<br>β = -0.17 ns<br>Both controlling for optimism and mindfulness
98	Trindade et al. (2020)	40	Breast	AAQII 7	DASS21	-	Experiential avoidance at T1 significantly predicted depression at T2, whilst controlling for T1 depression, stress and experiential avoidance at T2<br>β = 0.45*<br>Experiential avoidance at T1 significantly predicted stress at T2, whilst controlling for T1 depression, stress and experiential avoidance at T2<br>β = 0.37*
24	Corman et al. (2022)	187	Blood	AFQ	PTSS T2	0.45***<br>Control for T1 anx/dep:<br>0.25*<br>-0.29**	Experiential avoidance was not significantly associated with a greater risk of developing PTSD symptoms at 5 months<br>OR = 1.67 *p* = 0.32

				AAQII10 greater acceptance		Control for T1 anx/dep:<br>-0.02ns	
10	Baziliansky and Cohen (2021)	153	Colorectal	AAQ9	BSI	Distress at T2: 0.03 ns	Experiential avoidance was not significantly associated with distress at T1<br>β = 0.16 ns<br>Experiential avoidance was not significantly associated with distress at T2 (6m)<br>β = 0.11 ns<br>Whilst controlling for education, marital status, chemotherapy, suppression, cognitive reappraisal, self-compassion and personal resilience
5	Aldaz et al. (2019)	31	Mixed	Experiential avoidance related to illness uncertainty (daily measure)	DT	-	Daily experiential avoidance significantly predicted distress<br>B = 1.28***
22	Ciarrochi et al. (2011)	107	Mixed	AAQII10	DT	-	Males: avoidance was significantly associated with higher distress<br>β = 0.58**<br>Females: avoidance was significantly associated with higher distress<br>β = 0.64**
					FACT-B EWB		Males: avoidance was significantly associated with lower emotional wellbeing whilst controlling for success at health value<br>β = -0.69**<br>Females: avoidance was significantly associated with lower emotional wellbeing whilst controlling for success at romantic relationships<br>β = -0.73**

Acceptance							
105	X. Xu et al. (2019)	156	Mixed	CPAQ pain	HADSanx<br>HADSdep<br>HADS distress	-0.52*<br>-0.61*<br>-0.63*	-
25	Costanzo et al. (2006)	64	Gynae	COPE	POMSanx<br>POMSdep	-0.19 ns<br>-0.16 ns<br>Both control for treatment intensity	-
					FACT EWB	0.12 ns<br>control for treatment intensity	
85	Shapiro et al. (2010)	283	Mixed	bCOPE	HADSanx	-	Acceptance was significantly associated with lower anxiety, controlling for; ethnic group, recurrence, disengagement, venting, instrumental support, self-blame, planning, humour, cognitive functioning, insomnia, and social receptivity<br>β = -0.80***
					HADSdep		Acceptance was significantly associated with lower depression, controlling for; behavioural disengagement, venting, self-blame, humour, religion, physical function, cognitive function, social function, appetite loss, benefit finding and hope<br>β = -0.33*
					QLQ EWB		Acceptance was significantly associated with increased emotional wellbeing, controlling for; ethnic group, self-distraction, behavioural disengagement, venting, self-blame, emotional processing, emotion expression, role functioning, cognitive function, social functioning, nausea/vomiting and insomnia<br>β = 2.28*
64	Nipp et al. (2016)	350	Mixed	COPE median	HADSanx	-	Acceptance was significantly associated with lower anxiety, controlling for; age, sex, marital status and cancer type<br>β = -0.34*
					HADSdep		

							Acceptance was significantly associated with lower depression, controlling for; age, sex, marital status and cancer type<br>β = -0.37*
93	Thune-Boyle et al. (2013)	155	Breast	COPE	HADS	-	Acceptance was significantly associated with reduced anxiety, controlling for; age, employment, feeling punished by god, optimism, denial, instrumental support, planning, self-blame, self-distraction and venting<br>β = -0.23**
95	Trevino et al. (2012)	53	Mixed	bCOPE coping factor	McGillanx	-	There was a non-significant association for acceptance and lower anxiety controlling for depression, grief, proactive, distancing, negative expression, support seeking and respite seeking<br>β = -0.16 ns
					McGilldep		Acceptance was not significantly associated with an increase in depression whilst controlling for anxiety, grief, proactive coping, distancing coping, negative expression coping, support seeking and respite seeking<br>β = 0.17 ns
26	Deimling et al. (2006)	321	Mixed	COPE 3 items	POMSanx	-	There was a non-significant association for acceptance and lower anxiety controlling for race, gender, age, optimism, type of cancer, stage, years, treatment, health conditions scale, functional difficulty, coping planning, venting, denial and seeking social support<br>B = -0.01 ns
					POMSdep		There was a non-significant association for acceptance and lower depression controlling for race, gender, age, optimism, type of cancer, stage, years, treatment, health conditions scale, functional difficulties, coping (planning, venting, denial, seeking social support)<br>β = -0.05 ns
81	Saniah and Zainal (2010)	141	Breast	bCOPE	HADS	-	There was no difference in acceptance levels between anxious cases and non-anxious cases - Mann Whitney U Test: 0.59, *p* = 0.55
59	Manne et al. (2018)	174	Gynae	Emotional acceptance	BDI	-0.39*	-

100	van Laarhoven et al. (2011)	92	Mixed curative	COPE dutch	BDI	-0.36*	Acceptance was significantly associated with lower depression whilst controlling for age<br>β = -0.34**
			Mixed palliative			-0.48*	Acceptance was significantly associated with lower depression whilst controlling for giving up<br>β = -0.36**
			Mixed curative		QLQ Emotional functioning	0.25*	Acceptance was significantly associated with an increase in emotional wellbeing<br>β = 0.24*
			Mixed palliative			0.39*<br>All controlling for age, sex, partner, education, employment	
3	Aguado Loi et al. (2013)	68	Breast	bCOPE	PHQ9	-	Acceptance was significantly associated with lower depression<br>β = -0.32**<br>Acceptance was significantly associated with greater depression controlling for age, depression history, challenge, positive reframing, self-blame, body image, family and peer support<br>β = 0.24*
89	Stanton et al. (2018)	460	Breast	COPE	CES	-	Acceptance was not significantly associated with depression controlling for age, ethnicity, marital status, income, employment, SES, cancer stage, assessment interval at which treatments ended, number of comorbidities, recruitment site, treatment, and hormone therapy<br>Intercept = -2.19***, linear slope ns
71	Priscilla et al. (2011)	105	Blood	bCOPE	Mini MDD	-	There were no significant between-group differences between those who were not depressed M = 52.4 and those who were depressed M = 54.9; z = -0.4; *p* = 0.3
19	Carver et al. (1993)	59	Breast	COPE 3 items	POMS	-0.47* (post-surgery)<br>-0.29* (3m)	For all time points acceptance was associated with a decrease in distress whilst controlling for various factors

						-0.43** (6m)<br>-0.27* (12m)	(coping, chemotherapy, preoperative distress) Pre-surgery: β = -0.27 sig<br>Post-surgery: β = -0.33 sig<br>6m: β = -0.23 ns
69	Politi et al. (2007)	91	Breast	Emotional acceptance	POMS	-0.46*	Acceptance was significantly associated with lower distress controlling for age<br>β = -0.49***
104	W. Xu et al. (2017)	176	gastro	Self-acceptance	GHQ distress	-0.39*	-
79	Roussi et al. (2007)	72	Breast	Cope short	POMS post-surgery	-0.38***	-
88	Stanton et al. (2002)	70	Breast	COPE	POMS	0.03 (3m)<br>-0.30* (12m)<br>Both controlling for age and baseline	-
29	Fox (2002)	75	Mixed	COPE 3	POMS	-	Acceptance was significantly associated with lower distress, controlling for; religious identity, problem focused coping, social support seeking, denial, emotional approach coping, age, children, weeks since treatment initiated, perceived chance of cure and treatment side effects<br>β = -0.25**
					IES	-	Acceptance was significantly associated with lower distress, controlling for; religious identity, problem focused coping, social support seeking, denial, emotional approach coping<br>β = -0.28*
61	Millmann (2019)	11	Ovarian	CPAQ pain	POMS	-0.34 ns	-
7	Asuzu and Elumelu (2013)	237	Mixed	bCOPE	FACT EWB	0.07 ns	-
102	Vickberg (2000)	169	Breast	bCOPE	MHI	0.07 ns	-

16	Brunault et al. (2016)	120	Breast	bCOPE	QLQ Emotional Functioning	-	Acceptance was significantly associated with increase in emotional quality of life, controlling for; age, tumour stage, pain severity, existence of major depressive disorder, personality disorder, use of hormone therapy and self-blame<br>B = 3.93***
28	Elumelu et al. (2015)	110	Breast	bCOPE	FACT EWB	-	There was no significant difference between those who reported ‘not at all or a little bit’ of acceptance coping and those who reported ‘somewhat to very much’<br>MD = -0.39 (95% CI -2.77, -1.99) *p* = 0.75
Cognitive fusion							
32	Gillanders et al. (2015)	105	Mixed	CFQ7	HADS	-	Cognitive fusion was significantly associated with anxiety whilst controlling for cognitive distress, cognitive avoidance, emotional distress, avoidance coping and self-compassion<br>β = 0.54***<br>Cognitive fusion was not significantly associated with depression whilst controlling for cognitive distress, cognitive avoidance, emotional distress, avoidance coping and self-compassion<br>β = 0.05 ns
75	Randell (2017)	75	Mixed	CFQ7	HADS distress	0.59**	
					HADS		Cognitive fusion was significantly associated with anxiety whilst controlling for fear of recurrence, valued living, mindfulness and experiential avoidance (BEAQ)<br>β = 0.41***<br>Cognitive fusion was not significantly associated with depression whilst controlling for fear of recurrence, valued living, mindfulness and experiential avoidance (BEAQ)<br>β = 0.11 ns
Present moment awareness (PMA)							
46	Lam et al. (2018)	212	Mixed	FFMQ	HADSanx	-	The low PMA profile was significantly associated with higher anxiety compared to those who demonstrated the

					HADSdep		high PMA profile whilst controlling for sociodemographic and medical variables<br>β = 3.28**<br>The low PMA profile was significantly associated with higher depression compared to those who demonstrated the high PMA profile whilst controlling for sociodemographic and medical variables<br>β = 4.06***
77	Romano (2014)	76	Mixed	MAAS	HADSanx	-	PMA was associated with lower anxiety<br>β = -0.27** direct effect in mediation model
					HADSdep		PMA was significantly associated with reduced dep<br>β = -0.24* direct effect in mediation model
4	Al-Ghabeesh et al. (2019)	234	Breast	MAAS	HADSanx	-	PMA was significantly associated with lower anxiety controlling for income and social support<br>β = -0.24*
					HADSdep		PMA was significantly associated with reduced depression controlling for social support<br>β = -0.20*
75	Randell (2017)	75	Mixed	MAAS	HADS distress	-0.50**	-
					HADSanx		PMA was not significantly associated with decreased anxiety controlling for experiential avoidance, psychological flexibility, cognitive fusion and valued living<br>β = -0.18 ns
					HADSdep		PMA was not significantly associated with decreased depression controlling for experiential avoidance, psychological flexibility, cognitive fusion and valued living<br>β = -0.06 ns
23	Corman et al. (2021)	187	Blood	FFMQ	HADSanx	-	PMA was not significantly associated with anxiety controlling for experiential avoidance, optimism and acceptance<br>β = -0.12 *p*=0.09
					HADSdep		PMA was not significantly associated with lower depression whilst controlling for experiential avoidance, optimism and acceptance<br>β = -0.06 *p* = 0.46

9	Banner (2009)	69	Breast	FFMQ	TAQanx	-	PMA was significantly associated with increased anxiety whilst controlling for spirituality and the spirituality x PMA interaction<br>β = 0.4**
					CES-D short dep		PMA was significantly associated with an increase in depression controlling for spirituality and the spirituality x PMA interaction<br>β = 0.03**
21	Cho et al. (2021)	78	Lung	MAAS	CES-Dep	-	PMA was significantly associated with lower depressive symptoms controlling for faith (mediation)<br>B = -3.69***
					IES		PMA was significantly associated with lower distress controlling for meaning/peace<br>B = -4.09*<br>And controlling for faith<br>B = -7.03***
43	Kersting (2012)	74	Mixed	MAAS	CES-Dep	-	PMA was significantly associated with lower depression whilst controlling for gender and time since diagnosis<br>B = -0.57**<br>However PMA did not significantly predict depression at 3 months controlling for depression T1, gender and time since diagnosis<br>B = -0.23 ns
20	Chen et al. (2021)	90	Colorectal	FFMQ total	BDI	-	PMA was significantly associated with lower depression whilst controlling for age, history of psychological illness, cancer threat appraisal, symptoms and functions<br>β = -0.14**
38	Hsieh et al. (2021)	116	Lung	FFMQ total	BDI-II	-	PMA was significantly associated with lower depression controlling for previous depressive illness, presence of meaning, search for meaning, global health status, quality of life (functioning, symptom distress), lung cancer specific symptom distress<br>B = -0.08*
108	Zhong et al. (2020)	292	GI	MAAS	GHQ total	-0.20***	PMA was significantly associated with lower distress controlling for perceived stress, clinical stage, age and gender

							β = -0.11*
49	Lei et al. (2021)	441	Lung	FFMQ total	DT	-	PMA had a direct negative effect on psychological distress (mediation)<br>β = -0.11**
24	Corman et al. (2022)	187	Blood	FFMQ T1	PTSS T2	-0.16 ns (controlling for anxiety and dep 0.04 ns)	-
55	Z. Liu et al. (2021)	290	Liver	FFMQ total	HADS	-	PMA had a direct negative effect on distress controlling for age, income, child class (mediation)<br>β = -0.69***
68	Ploumen (2017)	108	Mixed	TFMQ	IES	-	PMA was significantly associated with lower distress controlling for gender, age, treatment phase and type of cancer<br>B = -0.58***
					DT		PMA was significantly associated with distress controlling for gender, age, treatment phase and type of cancer<br>B = -0.38***
91	Tamagawa et al. (2013)	272	Breast	MAAS	POMS	-	PMA was significantly associated with lower distress controlling for age, repression and suppression traits<br>β = -0.40**
70	Poulin et al. (2016)	76	Mixed	FFMQ	SF12	0.64***	-
30	Garcia et al. (2021)	183	Mixed	MAAS	FACT EWB	0.34***	-
Self-as-context							
8	Babu (2020)	164	Mixed	SACS	DASS21anx<br>DASS21dep	-0.10 ns<br>-0.22**	-
Committed action							
97	Trindade et al. (2018b)	82	Breast	CAQ8	DASS21anx<br>DASS21dep<br>DASS21 stress	-0.48***<br>-0.53***<br>-0.46***	-
8	Babu (2020)	164	Mixed	CAQ8	DASS21anx<br>DASS21dep	0.36**<br>0.32**	-

Values							
52	Lewson et al. (2021)	203	Mixed	VQ progress<br>VQ obstruction	PROMIS anx	-0.36**<br>0.63**	-
				VQ progress<br>VQ obstruction	PROMIS dep	-0.42**<br>0.61**	
63	Mosher et al. (2017)	80	Breast	VQ progress<br>VQ obstruction	PROMIS anx	-0.36**<br>0.61**	-
				VQ progress<br>VQ obstruction	PROMIS dep	-0.41**<br>0.56**	
62	Mosher et al. (2021)	201	Mixed	VQ progress<br>VQ obstruction	PROMIS anx	-0.34**<br>0.61**	-
				VQ progress<br>VQ obstruction	PROMIS dep	-0.39**<br>0.66**	
8	Babu (2020)	164	Mixed	Values importance	DASS21 anx	-0.14ns	-
					DASS21 dep	-0.21**	
47	Lampic et al. (2002)	32	Breast	Life Value	HADS anx	-	At 3 months, anxiety cases (C; M = -134, SD = 43) reported higher attainment-importance discrepancy scores for harmony values than for non-anxious cases (NC; M = -44, SD = 62); *t* = 4.00**<br>At 1 year, anxiety cases reported significantly higher attainment-importance discrepancy scores for harmony values and positive relation values<br>Positive relations: NC M = -24, SD = 75; C = -135, SD = 149; *t* = 2.79*<br>Harmony: NC M = -34, SD = 43; C = -127, SD = 142; *t* = 2.75*
					HADS dep		

							At 3 months depression cases reported higher attainment-importance discrepancy scores for all life values except comfort<br>Positive relations: NC M = -49, SD = 91; C M = -218, SD = 202; *t* = 3.13*<br>Involvement: NC M = -47, SD = 87; C M = -215, SD = 175; *t* = 3.40*<br>Responsibility: NC M = 20, SD = 53; C M = -31, SD = 46; *t* = 2.05*<br>Harmony: NC M = -53, SD = 62; C M = -158, SD = 38; *t* = 3.63***<br>Health: NC M = -131, SD = 150; C M = -340, SD = 152; *t* = 2.87**<br>Spirituality: NC M = 1, SD = 45; C M = -107, SD = 136; *t* = 3.48**<br>At 1 year, depression cases reported higher attainment-importance discrepancy scores for all values except comfort and responsibility<br>Positive relations: NC M = -33, SD = 86; C M = -240; SD = 129; *t* = 4.23***<br>Involvement: NC M = -48, SD = 109; C M = -225, SD = 189; *t* = 2.77**<br>Harmony: NC M = -41, SD = 60; C M = -225, SD = 145; *t* = 4.66***<br>Health: NC M = -119, SD = 142; C M = -288, SD = 193; *t* = 2.13*<br>Spirituality: NC M = -9, SD = 59; C M = -125, SD = 185; *t* = 2.63*
22	Ciarrochi et al. (2011)	107	Mixed	PVQ<br>Value success:	DT		-
				Family		-0.34*	
				Leisure		-0.43*	
				Health		-0.26**	
				Spirituality		-0.16 ns	
				Greater			

				commitment to:<br>Family values		 <br>-0.31**	
				PVQ Value success:<br>Family<br>Leisure<br>Health<br>Spirituality<br>Greater commitment to:<br>Family values	FACT EWB	 <br><br>0.50*<br>0.45*<br>0.34*<br>0.37**<br><br><br><br><br>-0.29**	Males: success in health was associated with greater emotional wellbeing whilst controlling for avoidance<br>β = 0.31**<br><br>Females: success in romantic relationships was associated with lower emotional wellbeing whilst controlling for avoidance<br>β = -0.26**
Committed action and values							
75	Randell (2017)	75	Mixed	ELS	HADS anx<br>HADS dep<br>HADS distress	-0.50**<br>-0.50**<br>-0.56**	Engaged living was not significantly associated with anxiety<br>β = -0.16 ns<br>Engaged living was significantly associated with lower depression<br>β = -0.33*
Self-compassion							
110	Zhu et al. (2019)	243	Mixed	SCS SF and subscales	STAI6	Positive self-compassion<br>-0.21*<br>Negative self-compassion<br>-0.34*<br>(Higher scores, lower negative self-compassion)	Overall self-compassion significantly predicted anxiety at T2 whilst controlling for T1 symptoms and education and gender but was non-significant for T3 controlling for education<br>β = T2 -0.23**, T3 -0.16 ns<br>Positive self-compassion significantly predicted anxiety at T2 whilst controlling for T1 symptoms, negative self-compassion, education and gender<br>β = -0.21**<br>Negative self-compassion did not significantly predict anxiety at T2 or T3

							β = T2 -0.12, T3 -0.08<br>Positive self-compassion significantly predicted anxiety at T3 whilst controlling for negative self-compassion and T1 symptoms<br>β = -0.18*
					PHQ9	Positive self-compassion<br>-0.12 ns<br>Negative self-compassion<br>-0.40** (higher scores, lower negative self-compassion)	Self-compassion did not significantly predict lower depression at T2 whilst controlling for T1 symptoms<br>β =-0.13 ns<br>Positive self-compassion did not significantly predict depression at T2 whilst controlling for education and T1 symptoms and negative self-compassion<br>β = -0.09 ns<br>Positive self-compassion significantly predicted depression at T3 whilst controlling for gender, T1 symptoms and negative self-compassion<br>β =-0.17*<br>Negative self-compassion did not significantly predict depression at T2 or T3 whilst controlling for positive self-compassion and T1 symptoms<br>β = T2 -0.13, T3 0.07
109	Zhu et al. (2020)	301	Mixed	SCS SF and subscales	STAI6	Positive self-compassion<br>-0.14*<br>Negative self-compassion<br>-0.41*<br>(Higher scores, lower negative self-compassion)	Self-compassion was significantly associated with lower anxiety whilst controlling for education and cancer recurrence<br>-0.17**
					PHQ9	Positive self-compassion<br>-0.06 ns<br>Negative self-compassion<br>-0.44*	Self-compassion total was significantly associated with lower depression, controlling for; education and cancer recurrence<br>-0.19**

						(higher scores, lower negative self-compassion)	
37	Ho et al. (2022)	127	Colorectal	SCS	HADSanx	T1: Positive self-compassion 0.13 ns Negative self-compassion 0.61*, T2: Positive self-compassion 0.02 ns, Negative self-compassion 0.45*	-
					HADSdep	T1: Positive self-compassion 0.05 ns, Negative self-compassion 0.52* T2: Positive self-compassion -0.11 ns Negative self-compassion 0.42*	
					PANAS	T1: Positive self-compassion 0.07 ns Negative self-compassion 0.65* T2: Positive self-compassion -0.03 ns Negative self-compassion 0.48*	
67	Pinto-Gouveia et al. (2014)	63	Mixed	SCS and subscales	DASS42anx	Positive self-compassion -0.10 ns	-
					DASS42dep	Positive self-compassion	

					DASS42 stress<br><br>WHO-QoL-BREF psych QoL	-0.59*<br>Positive self-compassion<br>-0.58*<br>0.51 **	Positive self-compassion was significantly associated with lower depression<br>β =-0.54***<br>Positive self-compassion was significantly associated with lower stress<br>β = -0.53 ***<br>Self-compassion significantly increased emotional wellbeing<br>β = 0.40**
33	Glover (2015)	155 (those who felt personally responsible for cancer)	Mixed	SCS	HADSanx	-	Self-compassion was significantly associated with lower anxiety whilst controlling for gender, age, site, time since diagnosis, recurrence, recruitment source and personal responsibility<br>B = -3.89***
					HADSdep		Self-compassion was significantly associated with lower depression whilst controlling for gender, age, site, time since diagnosis, recurrence, recruitment source and personal responsibility<br>B = -3.02***
94	Todorov et al. (2019)	195	Breast	SCS SF	DASS21anx<br><br>DASS21dep<br><br>DASS21 stress	-	Self-compassion was significantly associated with lower anxiety whilst controlling for marriage status, country of birth, chemotherapy status, hormone therapy status, targeted treatment status, reconstruction status, education, employment, age and time since diagnosis<br>β = -0.44**<br>Self-compassion was significantly associated with lower depression whilst controlling for covariates as above<br>β = -0.59**<br>Self-compassion was significantly associated with lower emotional stress whilst controlling for covariates as above<br>β = -0.70**
32	Gillanders et al. (2015)	105	Mixed	SCS	HADSanx		Self-compassion was not significantly associated with lower anxiety when controlling for cognitive distress, cognitive avoidance, emotional distress, avoidance coping and cognitive fusion<br>β = 0.01 ns

					HADSdep		Self-compassion was not significantly associated with lower depression when controlling for cognitive distress, cognitive avoidance, emotional distress, avoidance coping and cognitive fusion<br>β = -0.17 ns
99	van der Donk et al. (2020)	245	Mixed	SCS 24 (Dutch) subscales	CES-d	Positive self-compassion<br>-0.16*<br>Negative self-compassion<br>0.42**	-
					PANAS	Positive self-compassion<br>-0.11 ns<br>Negative self-compassion<br>0.47**	
87	Siwik et al. (2021)	58	Lung	SCS SF	CES-d	-	Self-compassion was significantly associated with lower depression<br>β = -0.64***
51	Levkovich (2021)	170	Breast	SCS SF	BSI	-	Self-compassion was significantly associated with lower distress whilst controlling for employment status, time since chemo, subjective stress and emotional control<br>β = -0.12*
10	Baziliansky and Cohen (2021)	153	Colorectal	SCS SF	BSI	-	Self-compassion significantly predicted an increase in distress at T2 controlling for education, marital status, chemo, suppression, cognitive reappraisal, experiential avoidance and personal resilience<br>β = 0.32 **
76	Raque-Bogdan et al. (2019)	275	Breast	SCS 26	FACT EWB	0.56**	-
30	Garcia et al. (2021)	183	Mixed	SCS 26 item	Emotional wellbeing	0.36*	-
Psychological flexibility							

60	McAteer and Gillanders (2019)	286	Prostate	CompACT	DASS21	-0.69**	Psychological flexibility was significantly associated with reduced distress controlling for age, self-esteem and stoicism<br>β = -0.41***
84	Sevier-Guy et al. (2021)	144	Prostate	CompACT	DASS21	-0.67**	Psychological flexibility was significantly associated with lower distress controlling for fear of recurrence<br>β = -0.56***

Notes: BEAQ: Brief Experiential Avoidance Questionnaire; HADS: Hospital Anxiety and Depression Scale; AAQ: Acceptance and Action Questionnaire; IDAS: Inventory of Depression and Anxiety Symptoms; GAD: Generalised Anxiety Disorder Scale; AFQ: Avoidance and Fusion Questionnaire; PHQ: Patient Health Questionnaire; DASS: Distress, Anxiety and Stress Scales; BSI: Brief Symptom Inventory; DT: Distress Thermometer; PTSS: Post Traumatic Symptoms Scale; PIPS: Psychological Inflexibility in Pain Scale; POMS: Profile of Mood States; PANAS: Positive and Negative Affect Schedule; FACT-B EWB: Functional Assessment of Cancer Therapy Emotional Wellbeing subscale; CPAQ: Chronic Pain Acceptance Questionnaire; COPE: Coping Orientation to Problems Experienced Inventory; bCOPE: Brief COPE; McGill: McGill Quality of Life Questionnaire; BDI: Beck Depression Inventory; CES-D: Centre for Epidemiologic Studies Depression; Mini MDD: Mini International Neuropsychiatric Interview for Major Depressive Disorder; GHQ: General Health Questionnaire; IES: Impact of Events Scale; MHI: Mental Health Inventory; CFQ: Cognitive Fusion Questionnaire; FFMQ: Five Facets of Mindfulness Scale; MAAS: Mindful Attention Awareness Scale; TAQ: Trimodal Anxiety Questionnaire; EORTC QLQ: European Organisation for Research and Treatment of Cancer Quality of Life Core Questionnaire; SF12: Short Form Health Survey; SACS: Self-as-context Scale; CAQ: Committed Action Questionnaire; VQ: Valuing Questionnaire; PROMIS: Patient Reported Outcomes Measurement Information System; PVQ: Personal Values Questionnaire; ELS: Engaged Living Scale; SCS: Self-compassion Scale (SF – Short Form); STAI: State Trait Anxiety Scale; WHO QoL: World Health Organisation Quality of Life Scale; CompACT: Comprehensive Assessment of Acceptance and Commitment Therapy processes.

S7. Narrative synthesis of additional data for meta-analysed processes

Experiential avoidance

Further data supported meta-analysis results, with greater experiential avoidance significantly associated with greater distress in cross-sectional regression analysis (22,23,34,39,77) and longitudinal regression analysis (5,11,13,98) whilst controlling for covariates. However, some studies found non-significant findings in cross-sectional regression analysis for experiential avoidance and depression (23,75) and distress (10). Furthermore, non-significant associations were found in longitudinal data for experiential avoidance and anxiety (48), depression (13,48) and distress (10,24). Experiential avoidance had a significant negative association with emotional wellbeing (13,22) although in this small sample it did not significantly predict emotional wellbeing at 6 months (13).

Acceptance

Data support the meta-analysis results with acceptance (pain, self and emotional) associated with lower anxiety, depression and distress in correlations (59,69,104,105). Data also supports meta-analysis results from cross-sectional regression analysis (3,29,64,69,85,93,100) and two longitudinal studies (19,88); which controlled for covariates. Data for emotional wellbeing were mixed, with three studies finding significant positive associations in cross-sectional regression analysis (16,85,100), whilst three studies found non-significant positive correlations (7,25,102). A variety of studies reported non-significant findings (25,26,61,89,95), whilst one study reported acceptance was significantly associated with an increase in depression whilst controlling for covariates (3). Three studies reported non-significant differences in between group analysis (see supplementary materials; 28,71,81).

Cognitive fusion

Studies supported the meta-analysis results with cognitive fusion associated with greater anxiety in cross-sectional regression analyses whilst controlling for covariates, but non-significant for depression (32,75).

Present moment awareness

Studies supported meta-analysis findings with present moment awareness significantly associated with lower anxiety, depression and distress in cross-sectional regression analysis whilst controlling for covariates (4,20,21,38,43,46,49,55,68,77,91,108). However, in two cross-sectional studies, present moment awareness was not significantly associated with lower anxiety or depression whilst controlling for other ACT processes (23,75) and was found to be positively associated in a small sample study (9). Longitudinal data also did not support meta-analyses, as present moment awareness was not significantly associated with lower depression or post-traumatic stress symptoms at follow ups whilst controlling for covariates (24,43). Present moment awareness was significantly positively correlated with emotional wellbeing (30,70).

Self-compassion

Twelve studies had additional data that supported the meta-analysis results with self-compassion significantly associated with lower anxiety, depression, distress, negative affect and emotional stress in cross-sectional regression analysis (33,51,67,87,94,109) and longitudinal regression analysis (110) whilst controlling for covariates. Three studies found significant positive associations with emotional wellbeing across correlations (30,67,76) and cross-sectional regression analysis (67). However, there were a few non-significant findings for anxiety and depression in cross-sectional regression analysis (32), and in regression analysis at start (T2) and end (T3) of treatment (110), both whilst controlling for covariates. One study found self-compassion predicted an increase in distress at 6 months (10).

Table S8. *Risk of bias assessment*

Study ID	Study design	i	ii	iii	iv (L only)	v	vi	vii	viii	ix	x	xi	xii	Overall
1	CS	L	U	U	n/a	L	L	U	U	M	L	n/a	L	M
2	CS	L	M	M	n/a	L	L	U	L	M	L	n/a	L	M
3	CS	L	M	U	n/a	L	L	L	H	L	M	n/a	L	M
4	CS	L	U	L	n/a	L	L	U	L	L	M	n/a	L	L
5	L (micro)	L	L	U	L	L	L	L	L	M	n/a	M	L	M
6	CS	L	M	H	n/a	L	L	L	U	M	U	n/a	L	H
7	CS	L	L	U	n/a	L	L	U	U	M	n/a	n/a	L	M
8	CS	L	L	U	n/a	L	L	L	L	L	L	n/a	L	L
9	CS	L	L	H	n/a	L	L	U	L	L	L	n/a	L	L
10	L	L	L	L	L	M	L	U	L	L	L	M	L	L
11	L	L	L	L	L	L	L	U	L	L	L	L	L	L
12	CS	L	L	U	n/a	L	L	H	U	L	n/a	n/a	L	L
13	L	L	L	U	L	L	L	L	L	L	n/a	L	L	L
14	CS	L	L	U	n/a	L	L	U	U	L	n/a	n/a	L	L
15	CS	L	U	U	n/a	L	L	U	M	H	n/a	n/a	L	M
16	CS	L	L	M	n/a	L	L	L	L	L	L	n/a	L	L
17	L	L	L	H	L	L	L	U	H	L	L	L	L	H
18	CS	L	M	L	n/a	L	L	L	U	L	n/a	n/a	L	L
19	L	L	U	L	U	L	L	U	U	L	L	L	L	L
20	CS	L	U	L	n/a	L	L	U	L	L	U	n/a	L	L
21	CS	L	L	M	n/a	L	L	L	U	M	L	n/a	L	L
22	CS	L	U	L	n/a	L	L	L	L	L	L	n/a	L	L
23	CS	L	L	U	n/a	L	L	H	L	L	L	n/a	L	L
24	L	L	L	U	M	L	L	U	L	M	L	L	L	L
25	CS	L	U	U	n/a	L	L	U	L	M	L	n/a	L	L
26	CS	L	L	H	n/a	L	L	U	L	L	L	n/a	L	L
27	CS	L	L	U	n/a	L	L	U	U	M	n/a	n/a	L	L
28	CS	L	U	U	n/a	L	L	U	H	H	n/a	n/a	L	H
29	CS	L	L	H	n/a	L	L	U	L	L	L	n/a	L	L
30	CS	L	L	L	n/a	L	L	U	L	L	L	n/a	L	L

Study ID	Study design	i	ii	iii	iv (L only)	v	vi	vii	viii	ix	x	xi	xii	Overall
31	CS	L	U	U	n/a	L	L	L	U	M	n/a	n/a	L	L
32	CS	L	U	H	n/a	L	L	L	L	L	U	n/a	L	L
33	CS	L	L	H	n/a	L	L	L	L	L	M	n/a	L	L
34	CS	L	L	U	n/a	L	L	H	L	M	M	n/a	L	M
35	CS	L	L	U	n/a	L	L	H	L	L	L	n/a	L	L
36	CS	L	L	M	n/a	L	L	U	U	M	n/a	n/a	L	M
37	L	L	L	U	L	L	L	L	L	L	U	L	L	L
38	CS	L	U	U	n/a	L	L	U	L	L	U	n/a	L	L
39	CS	L	L	M	n/a	L	L	H	L	L	L	n/a	L	L
40	CS	L	L	L	n/a	L	L	L	U	M	n/a	n/a	L	L
41	CS	L	L	H	n/a	L	L	U	H	L	L	n/a	M	M
42	CS	L	L	U	n/a	L	L	U	U	M	M	n/a	L	M
43	L	L	U	U	L	L	L	L	L	L	L	L	L	L
44	CS	L	L	M	n/a	L	L	M	L	L	U	n/a	L	L
45	CS	L	L	U	n/a	L	L	U	U	M	M	n/a	L	M
46	CS	L	L	H	n/a	L	L	L	U	M	M	n/a	L	H
47	L	L	L	L	U	M	L	U	U	L	L	H	L	M
48	L	L	U	U	L	L	L	U	L	M	M	H	L	H
49	CS	L	L	L	n/a	L	L	U	L	U	n/a	n/a	L	L
50	CS	L	L	U	n/a	L	L	M	U	M	M	n/a	L	M
51	CS	L	L	L	n/a	L	L	U	U	L	L	n/a	L	L
52	CS	L	L	U	n/a	L	L	U	L	U	n/a	n/a	L	L
53	CS	L	U	U	n/a	L	L	L	U	M	n/a	n/a	L	L
54	CS	L	U	U	n/a	L	L	L	U	M	n/a	n/a	L	L
55	CS	L	M	L	n/a	L	L	U	U	L	L	n/a	L	L
56	L	L	L	U	L	L	L	U	L	L	L	L	L	L
57	CS	L	L	L	n/a	L	L	U	U	U	n/a	n/a	L	L
58	CS	L	L	U	n/a	L	L	U	M	M	n/a	n/a	L	M
59	CS	L	L	H	n/a	L	L	U	U	L	L	n/a	L	L
60	CS	L	L	U	n/a	L	L	L	L	L	L	n/a	L	L
61	CS	L	U	U	n/a	L	L	U	H	H	High	n/a	L	H

Study ID	Study design	i	ii	iii	iv (L only)	v	vi	vii	viii	ix	x	xi	xii	Overall
62	CS	L	L	U	n/a	L	L	L	L	L	L	n/a	L	L
63	CS	L	L	L	n/a	L	L	U	U	M	n/a	n/a	L	L
64	CS	L	L	U	n/a	L	L	U	U	M	L	n/a	L	L
65	CS	L	U	U	n/a	L	L	U	U	M	n/a	n/a	L	M
66	CS	L	U	L	n/a	L	L	U	L	H	n/a	n/a	L	M
67	CS	L	L	U	n/a	L	L	U	U	L	L	n/a	L	L
68	CS	L	U	L	n/a	L	L	U	L	M	L	n/a	L	L
69	CS	L	L	U	n/a	L	L	L	U	L	L	n/a	L	L
70	CS	L	L	H	n/a	L	L	M	L	M	L	n/a	L	M
71	CS	L	L	L	n/a	L	L	U	U	M	L	n/a	L	L
72	CS (RCT)	L	L	U	n/a	L	L	U	L	M	n/a	n/a	L	L
73	CS (RCT)	L	L	U	n/a	L	L	U	L	M	n/a	n/a	L	L
74	CS	L	L	H	n/a	L	L	U	U	L	M	n/a	L	M
75	CS	L	L	U	n/a	L	L	L	L	L	M	n/a	L	L
76	CS	L	L	U	n/a	L	L	L	U	M	n/a	n/a	L	L
77	CS	L	L	U	n/a	L	L	L	L	M	n/a	n/a	L	L
78	CS	L	L	M	n/a	L	L	U	U	L	L	n/a	L	L
79	L	L	U	U	L	L	L	U	U	M	L	L	L	M
80	CS	L	L	U	n/a	L	L	U	L	L	M	n/a	L	L
81	CS	L	M	U	n/a	L	L	U	L	M	n/a	n/a	L	M
82	CS	L	L	L	n/a	L	L	U	L	L	L	n/a	L	L
83	CS	L	M	U	n/a	L	L	U	U	L	L	n/a	L	L
84	CS	L	L	U	n/a	L	L	L	L	L	n/a	n/a	L	L
85	CS	H	M	L	n/a	L	L	U	L	H	n/a	n/a	L	H
86	CS	L	L	M	n/a	L	L	L	L	L	M	n/a	L	L
87	CS	L	U	U	n/a	L	L	M	L	M	n/a	n/a	L	M
88	L	L	U	L	L	L	L	U	H	L	L	L	L	L
89	L	L	U	U	M	L	L	L	L	L	L	L	L	L
90	CS	L	L	U	n/a	L	L	L	L	M	n/a	n/a	L	L
91	CS	L	L	U	n/a	L	L	U	L	L	L	n/a	L	L
92	CS	L	L	L	n/a	L	L	L	U	L	L	n/a	L	L

Study ID	Study design	i	ii	iii	iv (L only)	v	vi	vii	viii	ix	x	xi	xii	Overall
93	CS	L	L	M	n/a	L	L	U	L	L	L	n/a	L	L
94	CS	L	U	H	n/a	L	L	H	L	L	M	n/a	L	H
95	CS	L	L	U	n/a	L	L	U	H	L	L	n/a	L	L
96	CS	L	L	U	n/a	L	L	U	U	M	n/a	n/a	L	L
97	CS	L	L	U	n/a	L	L	U	U	L	L	n/a	L	L
98	L	L	U	U	U	L	L	U	U	L	L	L	L	U
99	CS (CC)	L	L	H	n/a	L	L	U	L	M	n/a	n/a	L	M
100	CS	L	L	L	n/a	L	L	L	L	L	L	n/a	L	L
101	CS	L	U	L	n/a	L	L	L	L	L	L	n/a	L	L
102	CS	L	L	H	n/a	L	L	L	U	L	M	n/a	L	M
103	CS	L	L	U	n/a	L	L	L	U	M	n/a	n/a	L	L
104	CS	L	U	L	n/a	L	L	L	U	M	n/a	n/a	L	L
105	CS	L	L	L	n/a	L	L	H	L	M	n/a	n/a	L	L
106	CS	L	L	U	n/a	L	L	M	U	L	M	n/a	L	M
107	CS	L	M	U	n/a	L	L	U	U	M	L	n/a	L	M
108	CS	L	U	U	n/a	L	L	U	L	M	M	n/a	L	M
109	CS	L	L	L	n/a	L	L	U	U	L	M	n/a	L	L
110	L	L	L	L	M	L	L	U	U	L	L	L	L	L

Note: CS: cross sectional; L: longitudinal; C (RCT); cross sectional/baseline analysis of an RCT; CS (CC): cohort study; i: bias in selection of reported outcomes; ii: selection bias; iii: response bias; iv: attrition bias (longitudinal only); v: valid predictor measure; vi: valid outcome measure; vii: bias due to missing data; viii: sample size a priori; ix: appropriate analysis used to control for key confounding variables; x: were confounders measured validly and reliably; xi: follow up time controlled; xii: significance levels reported; L: low risk of bias; M: moderate risk of bias; H: high risk of bias; U: unclear risk of bias; n/a: not applicable

Tables S9. *GRADE quality assessment*

Process and distress as outcome	No. of studies	Inconsistency	Imprecision	Publication bias (see funnel plots)	Reporting bias	Quality of the evidence (GRADE)
Experiential avoidance	17	**Serious** *Heterogeneity is 72%*	**Not serious** *Narrow CI width*	**Not serious** *Relative symmetry*	**Moderate** *12 low, 3 moderate, 1 high and 1 unclear*	**Low** *Downgraded 1 for inconsistency; upgraded for large effect*
Acceptance	16	**Serious** *Heterogeneity is 79%*	**Serious** *Moderate CI width crosses small/ medium effect*	**Serious** *Substantial asymmetry*	**Moderate** *8 low, 6 moderate and 2 high*	**Very low** *Downgraded 1 for inconsistency, imprecision and publication bias*
Cognitive fusion	8	**Serious** *Heterogeneity is 71%*	**Not serious** *Narrow CI width*	**Not assessed**	**Not serious** *7 low and 1 moderate*	**Low** *Downgraded 1 for inconsistency; upgraded for large effect*
Present moment awareness	30	**Very serious** *Heterogeneity is 92.5%*	**Not serious** *Narrow CI width*	**Serious** *Substantial asymmetry*	**Not serious** *25 low and 5 moderate*	**Very low** *Downgraded 1 for inconsistency and publication bias*
Self-compassion	20	**Serious** *Heterogeneity is 65.7%*	**Not serious** *Narrow CI width*	**Moderate** *Moderate asymmetry*	**Moderate** *12 low, 6 moderate and 2 high*	**Very low** *Downgraded 1 for inconsistency*

Funnel plots to assess potential publication bias for GRADE assessment

Funnel Plot for Experiential avoidance and distress

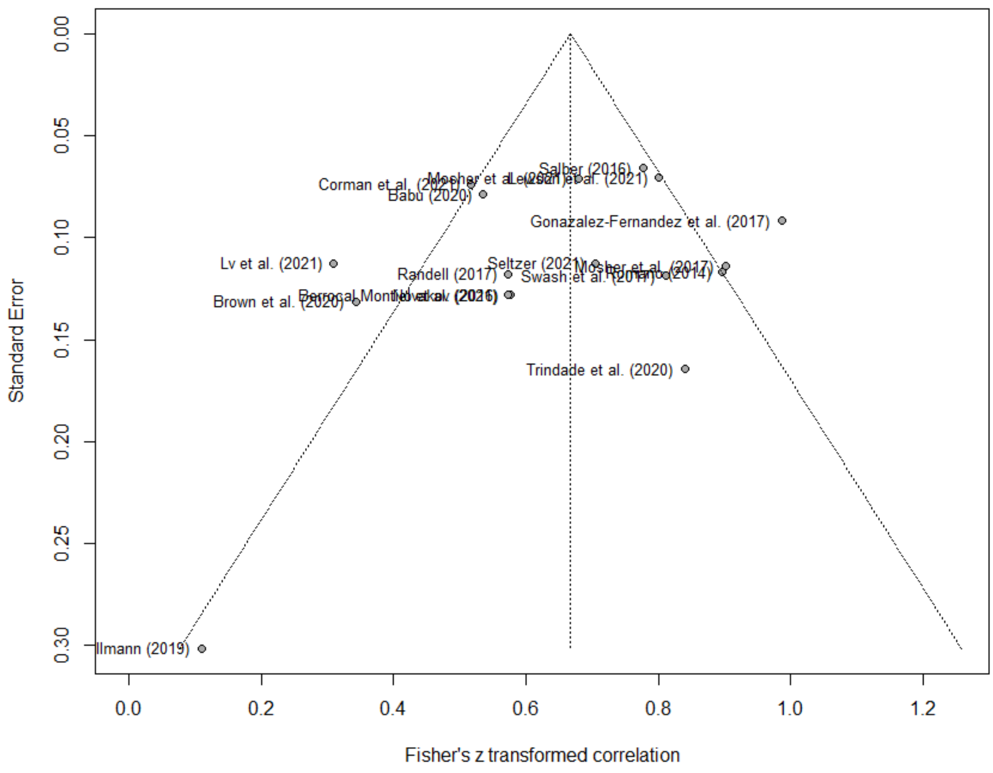

Funnel Plot for Acceptance and distress

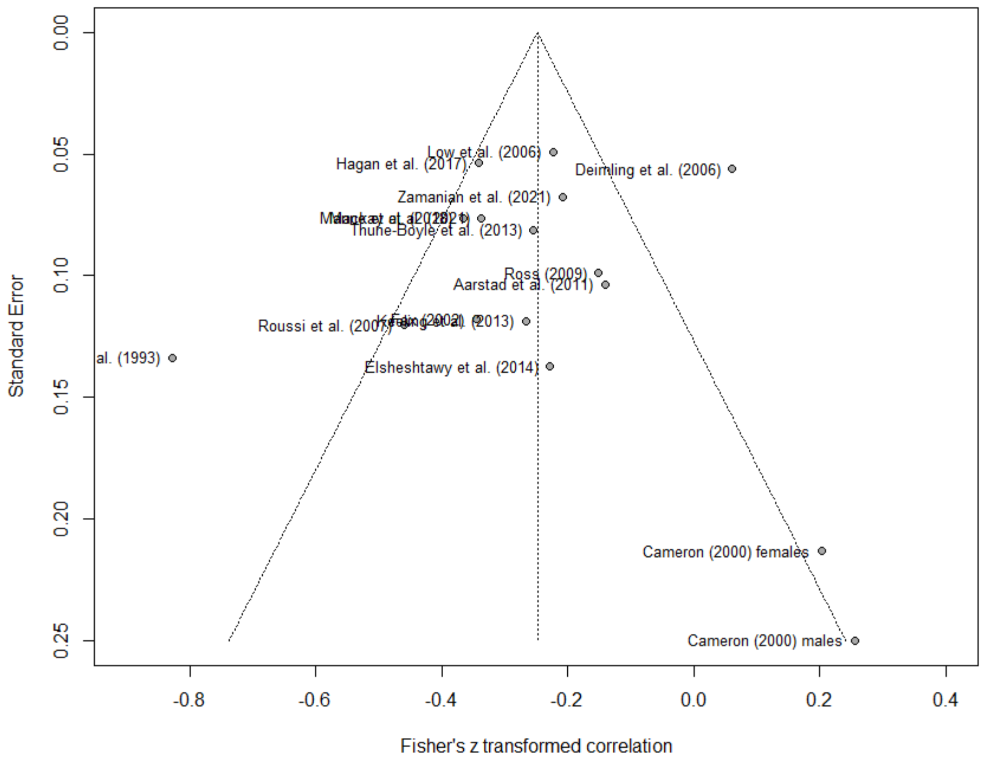

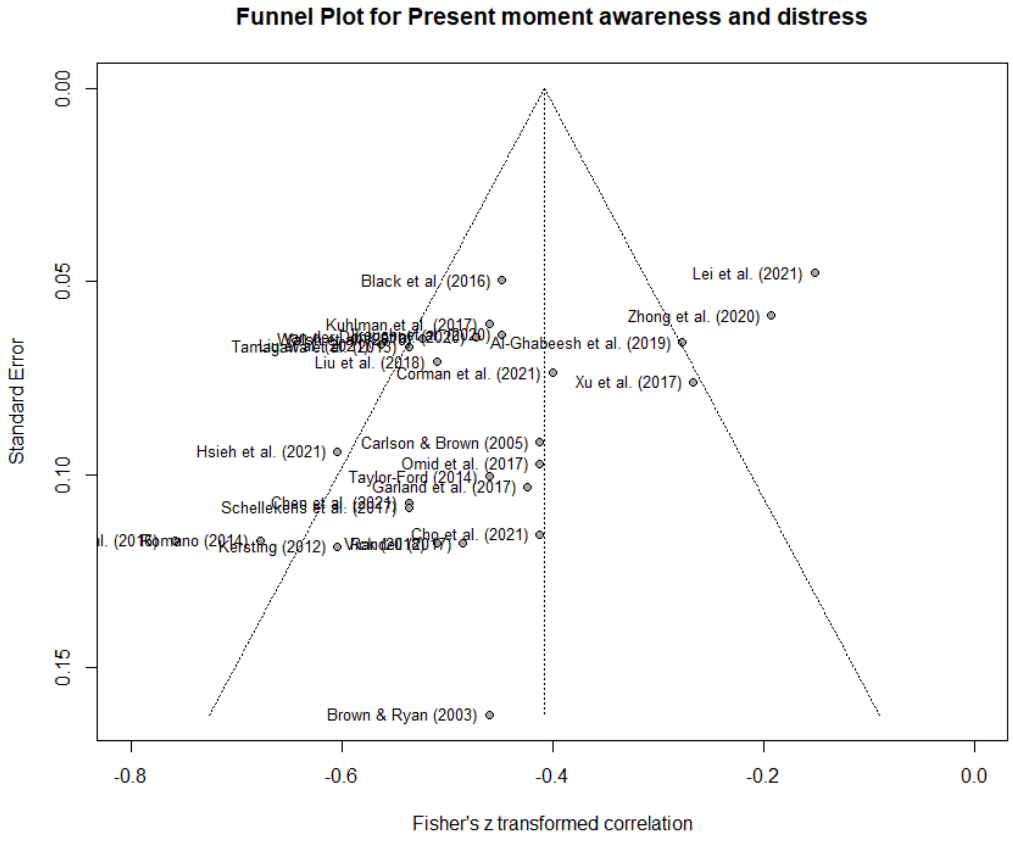

Funnel Plot for Present moment awareness and distress
Standard Error
0.00
0.05
0.10
0.15
-0.8
-0.6
-0.4
-0.2
0.0
Fisher's z transformed correlation
Black et al. (2016)
Lei et al. (2021)
Zhong et al. (2020)
Kuhlman et al. (2017)
Al-Ghabeesh et al. (2019)
Liu et al. (2018)
Corman et al. (2021)
Xu et al. (2017)
Hsieh et al. (2021)
Carlson & Brown (2005)
Omid et al. (2017)
Taylor-Ford (2014)
Garland et al. (2017)
Cho et al. (2021)
Kersting (2012)
Brown & Ryan (2003)

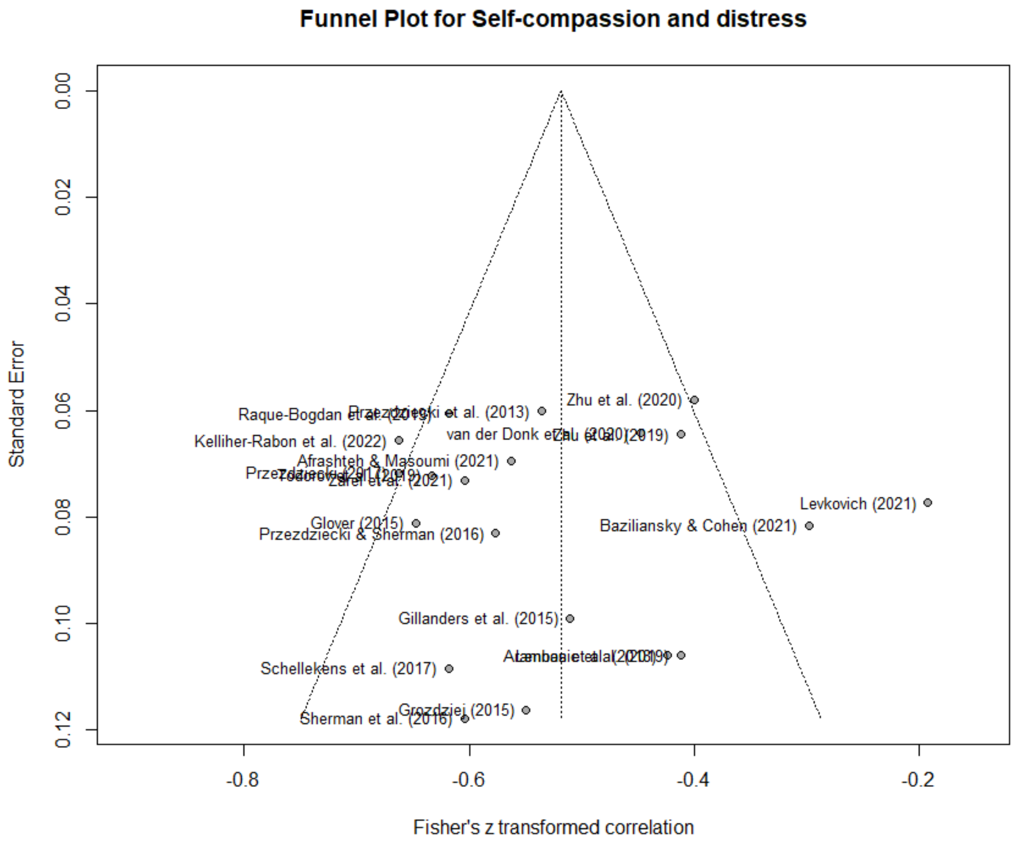

Across study bias assessment for processes included in narrative synthesis

For narrative synthesis, the majority of findings for experiential avoidance, acceptance, present moment awareness and self-compassion were consistent with meta-analyses, with only a few inconsistent findings. Studies for present moment awareness and self-compassion were mostly low risk of bias, however for experiential avoidance and acceptance, a larger proportion of studies had moderate, high or unclear risk of bias. Data for cognitive fusion is likely to have greater heterogeneity as findings for depression were inconsistent to the meta-analysis and with limited studies. Values and psychological flexibility resulted in expected directions with a low risk of bias for all studies. Self-as-context and committed action had mixed results. All four of these processes had a very small number of studies. Fewer studies explored processes with emotional wellbeing, however most studies scored low for risk of bias, findings were generally consistent and in expected directions. Overall narrative synthesis results should be interpreted with caution.
